# Supplementary material for: Understanding why primary care doctors leave direct patient care: a systematic review of qualitative research
Source: BMJ Open. 2020 May 12;10(5):e029846. doi: 10.1136/bmjopen-2019-029846 (PMC7228506; doi:10.1136/bmjopen-2019-029846)
Supplement: Author's manuscript [file bmjopen-2019-029846.draft_revisions.pdf]

BMJ Open is committed to open peer review. As part of this commitment we make the peer review history of every article we publish publicly available.

When an article is published we post the peer reviewers' comments and the authors' responses online. We also post the versions of the paper that were used during peer review. These are the versions that the peer review comments apply to.

The versions of the paper that follow are the versions that were submitted during the peer review process. They are not the versions of record or the final published versions. They should not be cited or distributed as the published version of this manuscript.

BMJ Open is an open access journal and the full, final, typeset and author-corrected version of record of the manuscript is available on our site with no access controls, subscription charges or pay-per-view fees (http://bmjopen.bmj.com).

If you have any questions on BMJ Open's open peer review process please email info.bmjopen@bmj.com

BMJ Open

Understanding why primary care doctors leave direct patient care - A systematic review of qualitative research

Journal:	*BMJ Open*
Manuscript ID	bmjopen-2019-029846
Article Type:	Original research
Date Submitted by the Author:	18-Feb-2019
Complete List of Authors:	Long, Linda; University of Exeter, ESMI<br>Moore, Darren; University of Exeter,<br>Robinson, Sophie; University of Exeter Medical School, Evidence Synthesis & Modelling for Health Improvement, Institute of Health Research<br>Anderson, Rob; University of Exeter, ESMI (Evidence Synthesis & Modelling for Health Improvement)<br>Sansom, Anna; University of Exeter Medical School, Primary Care<br>Aylward, Alex; Patient and Public Involvement Group<br>Fletcher, Emily; University of Exeter Medical School, Primary Care Research Group<br>Welsman, Jo; University of Exeter, Children's Health and Exercise Research centre<br>Dean, Sarah; PenCLAHRC University of Exeter Medical School,<br>Campbell, John; University of Exeter, Primary Care;
Keywords:	general practitioner, systematic review, job satisfaction, leave, flexible working, burnout

SCHOLARONE™
Manuscripts

I, the Submitting Author has the right to grant and does grant on behalf of all authors of the Work (as defined in the below author licence), an exclusive licence and/or a non-exclusive licence for contributions from authors who are: i) UK Crown employees; ii) where BMJ has agreed a CC-BY licence shall apply, and/or iii) in accordance with the terms applicable for US Federal Government officers or employees acting as part of their official duties; on a worldwide, perpetual, irrevocable, royalty-free basis to BMJ Publishing Group Ltd ("BMJ") its licensees and where the relevant Journal is co-owned by BMJ to the co-owners of the Journal, to publish the Work in this journal and any other BMJ products and to exploit all rights, as set out in our licence.

The Submitting Author accepts and understands that any supply made under these terms is made by BMJ to the Submitting Author unless you are acting as an employee on behalf of your employer or a postgraduate student of an affiliated institution which is paying any applicable article publishing charge ("APC") for Open Access articles. Where the Submitting Author wishes to make the Work available on an Open Access basis (and intends to pay the relevant APC), the terms of reuse of such Open Access shall be governed by a Creative Commons licence – details of these licences and which Creative Commons licence will apply to this Work are set out in our licence referred to above.

Other than as permitted in any relevant BMJ Author's Self Archiving Policies, I confirm this Work has not been accepted for publication elsewhere, is not being considered for publication elsewhere and does not duplicate material already published. I confirm all authors consent to publication of this Work and authorise the granting of this licence.

For peer review only

Understanding why primary care doctors leave direct patient care - A systematic review of qualitative research

Linda Long[1], Darren Moore[2], Sophie Robinson[1], Rob Anderson[1], Anna Sansom[3], Alex Aylward, Emily Fletcher[3], Jo Welsman[4], Sarah G Dean[5], John Campbell[3]

[1] Evidence Synthesis & Modelling for Health Improvement, Institute of Health Research, University of Exeter Medical School

[2] Graduate School of Education, University of Exeter

[3] Primary Care Research Group and University of Exeter Collaboration for Academic Primary Care (APEx), Institute of Health Research, University of Exeter Medical School

[4] Centre for Biomedical Modelling and Analysis, Living Systems Institute, University of Exeter

[5] Psychology Applied to Health, Institute of Health Research, University of Exeter Medical School

Correspondence to Dr Linda Long: L.Long@exeter.ac.uk

Total word count (excluding abstract): 2494 words

How this fits in

The British GP workforce is said to be in "crisis" with between a third and two fifths of UK GPs intending to leave practice permanently within the next 5 years.

Given the scale of the problem, it is important to understand GP leaving behaviour in the UK.

This systematic review provides a deeper understanding of the complex interplay of key factors and contexts affecting UK GPs' decisions to leave practice.

This understanding can inform the development of UK GP retention initiatives at national, regional, local area/CCG or practice levels.

For peer review only - http://bmjopen.bmj.com/site/about/guidelines.xhtml

Abstract

Objectives: UK General Practitioners (GPs) are leaving direct patient care in significant numbers. We undertook a synthesis of qualitative research to identify factors affecting GPs' leaving behaviour in the workforce as part of a wider mixed methods study (ReGROUP). Our objectives were to identify factors that affect GPs' decisions to leave direct patient care.

Design/methods: Qualitative interview-based studies were identified and quality assessed. A thematic analysis was performed and an explanatory model constructed providing an overview of factors affecting UK GPs. Non-UK studies were considered separately.

Results: Six UK interview-based studies and one Australian interview-based study were identified. Three central dynamics key to understanding UK GP leaving behaviour were identified - factors associated with low job satisfaction, high job satisfaction, and those linked to the doctor-patient relationship. The importance of contextual influence on job satisfaction emerged. GPs with high job satisfaction described feeling supported by good practice relationships, while GPs with poor job satisfaction described feeling overworked and unsupported with negatively-impacted doctor-patient relationships.

Conclusions: Many GPs report that job satisfaction directly relates to the quality of the doctor-patient relationship. Combined with changing relationships with patients and interfaces with secondary care, and the gradual sense of loss of autonomy within the workplace, many GPs report a reduction in job satisfaction. Once job satisfaction has become negatively impacted, the combined pressures of increased patient demand and workload, together with other stress factors, has left many feeling unsupported and vulnerable to burnout and ill health, and, ultimately, to the decision to leave general practice.

Keywords: general practitioner, systematic review, job satisfaction, leave, flexible working, burnout

PROSPERO protocol CRD42016033876

For peer review only - http://bmjopen.bmj.com/site/about/guidelines.xhtml

Article summary

Strengths and limitations of this study:

- This systematic review offers a deeper understanding of the complex interplay of key factors and contexts affecting UK GPs' decisions to leave practice.
- Relevant stakeholder involvement in the review gives a good basis for transferability; several of the study team are GPs and were involved in developing the review protocol.
- Patients were involved through contributing to a Patient and Public Involvement workshop at which our explanatory model was discussed.
- Only a small number of UK studies were identified; although a single non-UK study was identified, we were not able to translate study findings across countries.
- Synthesis of qualitative evidence presented in this review relates largely to just NHS General Practice in England; however it seems like that many of the factors highlighted are generic within primary care in the rest of the UK.

For peer review only

Introduction

UK GPs are leaving direct patient care in significant numbers (1). We undertook a qualitative synthesis of the evidence to identify factors that affect GPs' retention in the workforce as part of a wider mixed methods study (ReGROUP) focusing on retention of experienced GPs or supporting their return to work following a career break. Through better understanding the factors that lead GPs - especially experienced GPs in the UK NHS - to leave direct patient care, the wider ReGROUP study (2) ultimately aims to inform policies and strategies to support GPs returning to work after a career break or retain the experienced GP workforce. By identifying and analysing rich qualitative data from a variety of GP interview studies, we sought to gain a deeper understanding of why GPs are leaving UK practice and to identify and understand how factors may act individually or collectively to affect such decisions.

Aims

This systematic review of qualitative evidence aimed to answer the following question:

What are the factors in the UK and other high income countries which affect GPs' decisions to leave direct patient care?

For peer review only

Methods

We conducted a systematic review of the qualitative literature in line with our published protocol.

Searches

In January 2016 and March 2016 articles published in English from 1990 onwards were searched in the following databases: Medline, Medline in Process, PsycInfo, HMIC (Healthcare Management Information Consortium), Cochrane, ASSIA (Applied Social Sciences Index of Abstracts) and Web of Science (Supplementary File). We performed grey literature searching including online searching, reference checking of relevant studies and forward and backward citation searching. Further update searches were performed in May 2017. Our search strategy is shown in Figure 1.

Figure 1 - Medline search strategy

Inclusion criteria

We included qualitative or mixed methods studies which either aimed to assess factors associated with GP leaving behaviour, or which are likely to have generated research data about such factors. We included studies with General Practitioners and other primary care-based generalist doctors practising in high-income countries (Supplementary File) where health systems tend to have general/primary care physicians working in non-hospital, community settings. We sought studies which evaluated any reasons for leaving direct patient care (e.g. early retirement, career breaks, moving to hospital specialities, commissioning or public health, working part-time, or never returning to work after paternal/maternal leave).

Exclusion criteria

Sources were excluded if they were not in English language or highly abbreviated source types (e.g. conference abstracts).

Study selection process

Titles and abstracts of search results were screened against the eligibility criteria, with an initial sample being independently screened by two authors (SR and RA) to establish consistent application of the criteria. Titles and abstracts that could not be excluded were sought as full text articles, and the inclusion criteria applied to these (Figure 2).

Figure 2 - PRISMA flow diagram showing process of study selection

Data extraction and quality appraisal

One reviewer (LL) data extracted all studies and 50% were independently checked by a second reviewer (DM), with any discrepancies resolved through discussion. Study quality was assessed using an adapted version of the Wallace checklist (3) by one reviewer (LL) and 50% independently checked by a second reviewer (DM).

Analysis and synthesis

Data analysis and synthesis broadly followed the principles of thematic synthesis (4) and were conducted in three stages which overlapped to some degree: the coding of text “line-by-line”; the organisation of these “free codes” into related areas to construct data-driven “descriptive themes”, and the development of theory-driven “analytical” themes through the application of a higher level theoretical framework. Synthesis methods broadly followed guidelines for thematic analysis of textual data collected in the context of primary research. In this case the textual data were study authors’ descriptions of their findings as well as primary quotations from GPs.

For peer review only - http://bmjopen.bmj.com/site/about/guidelines.xhtml

Of the included studies, two recent data-rich UK papers (5, 6)were coded by one reviewer (LL) and the descriptive themes used to create an overall analytical framework consisting of five categories. The same two key papers were independently coded by a second reviewer (DM) and the analytical framework agreed and modified through discussion. This framework was used to code the remaining studies by one reviewer (LL), with a sample checked by a second reviewer (DM) for consistency. Data, in the form of quotations from the GPs themselves, key concepts or succinct summaries of findings were entered into QSR's NVivo software (version 11)(7) for analysis. Descriptive and analytical themes emerging from the UK studies were white-boarded and associations considered. It was acknowledged that the identified themes could be relevant to more than one category and this was represented in a visual "explanatory model" (Figure 3) in order to answer the review question. The model was created by one reviewer (LL), independently checked by a second reviewer (DM) and modifications incorporated into the model after discussion. The model was presented and assessed in terms of credibility during an involvement workshop (4 patient participants) and through discussion with the wider ReGROUP project research team.

For peer review only

Results

Study Characteristics

Five studies (six publications) based on qualitative semi-structured interviews with practising or retired GPs were found (5, 6, 8-11), all conducted in England. A further qualitative semi-structured interview study conducted in Australia was found (12). The main characteristics of these studies are shown in Table 1.

Two of the papers reporting studies from England report findings from largely the same set of interviews (5, 6) with the later paper including a larger sample of interviewees, after intentionally recruiting more female GPs and more GPs aged 50-55 years (6).

For peer review only

For peer review only - http://bmjopen.bmj.com/site/about/guidelines.xhtml

Table 1 Characteristics of qualitative interview studies and included GPs

Study	Year of survey(s)	Country or Region	Types of GPs responding	Aim of study	No. GPs (interview setting)	Age of GPs	% female
Doran et al 2016(11)	NS	England	Early leavers age <50 years	To explore the reasons why GPs leave general practice early	21 (by phone)	median age-band 32-54 years	66.7%
Hutchins 2005(8)	NS	England (London)	GP principals near retirement age	Considers the reasons why many GPs are wishing to take early retirement, and measures to help retain them.	20 (at surgery)	NS	55%
Newton et al 2004(9)	NS	England (Northern)	Over 45	To describe "Plans, reasons for, and feelings about retirement"	21 (at surgery or GP home, except 2 by phone)	All over 45 years	38%
Sansom et al 2016*(6)	2015	England (South West)	Experienced GPs 50-60 years old (20 still working, 3 retired)	To investigate the reasons behind intentions to quit direct patient care among experienced general practitioners (GPs) aged 50-60 years.	23* (by phone)	Age range 51-60 years	39%
Campbell et al 2015*(5)	2014-15	England (South West)	Experienced GPs 50-60 years old intending to retire in next 5 years (n=14); GPs who took early retirement in last 5 years (n=3); 15 partners, 2 locums	To explore reasons behind GPs' intentions to quit direct patient care	17* (by phone)	Age 51-60 years	23.5%
Ipsos MORI 2015(10)		England	42 GPs seriously considering leaving practice as well as 23 GPs who had left or were in the process of returning to practice	To identify how the experience of appraisal and revalidation might be influencing intentions to leave general practice	42 (by phone) 23 (by phone)	NR	NR

For peer review only

Study	Year of survey(s)	Country or Region	Types of GPs responding	Aim of study	No. GPs (interview setting)	Age of GPs	% female
Dwan et al 2014(12)	2008 - 2009	Australia	GPs working six or fewer clinical sessions per week	To explore the nature and extent of GPs' paid and unpaid work, why some choose to work less than full-time, and whether sessional work reflects a lack of commitment to patient and the profession	26 (at a location determined by GP participant)	Average age: 47 years (females); 58 years (males)	66%

NS = not stated. *these studies were based on largely the same sample of GP interviews. The later study (Sansom et al, 2016) (6) purposively selected more female GPs and more GPs aged 50-55, to increase the variation of age and sex across the sample

For peer review only - http://bmjopen.bmj.com/site/about/guidelines.xhtml

Appraisal and Synthesis

The analysis and synthesis presented below is based on five UK interview-based studies reported in six papers/reports (5, 6, 8-11). The findings of the Australian study (12) are presented separately (Supplementary File) and discussed in relation to UK findings.

Quality Assessment

The quality of the included qualitative research studies and papers, as assessed using the 14 questions of the adapted 'Wallace tool'(3), ranged from low-quality (10), with 4/14 "yes" ratings on quality criteria, through to moderate-quality (8, 9), with 6/14 "yes" ratings on quality criteria, and up to good-quality (5, 6, 11, 12), with 9/14 "yes" ratings on quality criteria or better.

Most studies failed to make explicit the theoretical or ideological perspective of the author (Q2). No studies provided evidence of author reflexivity (Q13). Three UK studies (8-10) and one non-UK study (12) had further limitations in relation to two to four other quality criteria.

All of the themes in the synthesis were informed by at least two studies, and there was at least one good quality study informing every theme. The low to moderate-quality UK studies alone did not determine any of the themes, but did provide support for them.

Categories and themes

The synthesis consisted of a series of linked themes affecting whether GPs leave direct patient care or reduce their time commitment to patient care, each of which belongs to one of five categories summarized in the analytical framework below (Table 2).

Table 2 - Analytic framework showing identified categories and themes around GP's decisions to leave direct patient care

**Undoable /unmanageable**<br><br>- Workload<br>- Pressures<br>- Fear of making mistakes<br>- Training and resources<br>- Patient demands<br>- Practice demands	**Morale**<br><br>- Identity / perceived value<br>- Professional culture<br>- Lack of support<br>-Government/political<br>- Wider community<br>- Negative 'media-bashing'<br>- Job satisfaction<br>- Wellbeing<br>- Work/life balance	**Impact of Organisational Changes**<br><br>- Referrals<br>- Targets and assessments<br>- Doctor-patient relationship<br>- Changing role<br>- Autonomy and control<br>- Reaccreditation
**Projected Future**<br><br>- Viability (of early retirement)<br>- Ageing<br>- Investment and commitment	**Multiple Options and Strategies**<br><br>- Flexible working<br>- Continue and cope<br>- Alternative roles	

For peer review only - http://bmjopen.bmj.com/site/about/guidelines.xhtml

These categories from the qualitative synthesis were, firstly, GPs experiencing working as a GP as 'undoable and unmanageable'. Many GPs are experiencing working as a GP as undoable and unmanageable due, among other reasons, to high/increasing administrative workloads, high/increasing patient demand (both number of patients, and their complexity and higher expectations), together with a perceived lack of training and resources to cope with these pressures.

The second category, 'low morale', was seen to be associated with reductions in the perceived value of GP work (with loss of identity) and changed professional culture (more target- and standards-driven rewards system; multi-disciplinary team-based working (yet for some also lone working / isolating culture); a more aggressive top-down managerial culture within the NHS, and more widespread norms and expectations for early retirement). Low morale was seen as associated with a lack of support from both government and political parties, and negative portrayals of GPs by news media. Morale was also seen to be closely linked with job satisfaction (or dissatisfaction), neglect of personal wellbeing/health and feelings about work-life balance.

The third category was the 'impact of organisational changes'. The perceived key factors under this theme were changes in referrals - both restricted opportunities to refer to secondary care, and higher numbers of (and more complex) referrals from secondary care - as well as a greater focus on targets and assessments, and fears about re-accreditation (including evidence that some GPs might retire early in order to avoid re-accreditation). Some of the organisational changes were considered to have imposed increased clinical and non-clinical responsibilities and work on GPs. Together, such changes were believed to have undermined some of the basic tenets and traditional expectations of being a GP, such as the doctor-patient relationship and having autonomy and control over one's clinical work.

The fourth category was how GPs projected their future, which related to aging, the financial viability of reducing hours or retiring early, and to what extent GPs were personally committed and financially invested in their practices. This included problems linked to whether younger GPs wanted to take on the responsibility of becoming practice partners, and also possible tensions between older and younger GP partners (in the way practices are run, in major investment / refurbishment decisions, or in relation to planning for partner's retiring and needing new partners to buy out their share of a practice).

Finally, the fifth category was called 'multiple options and strategies' and referred to the various ways in which GPs either continue and cope or- perhaps if less committed or less resilient, or if they can simply afford to financially - decide to leave or go part-time. This theme also highlighted the major importance of flexible working i.e. working reduced hours (e.g. by becoming a locum) as a method of coping and regaining work-life balance and job satisfaction. For others, the adoption of alternative work roles outside general practice, often part-time, allowed use and learning of other skills – either as relief and variety from working as a GP, or for some as a potential alternative career. The kinds of alternative roles and options GP interviewees mentioned included becoming complementary therapists, CCG leads, advisory committee members, or working for pharmaceutical consultancies or teaching in medical schools. Like part-time working, for some these might be clear routes for quitting general practice; but for others, such variety of roles and opportunities for job satisfaction may keep them in general practice.

For peer review only - http://bmjopen.bmj.com/site/about/guidelines.xhtml

Explanatory model and narrative summary of key factors influencing UK GPs

Themes were used to construct an explanatory model (Figure 3). This model makes it possible to 'go beyond' the findings of the primary studies and generate additional concepts, understandings and hypotheses relating to factors influencing GPs' decisions to quit general practice. 'Real world' applicability was confirmed following feedback on the model from patients and project stakeholders.

Above the explanatory model (in grey), the changing nature of general practice over time is presented separately, providing a contextual lens from which to view the main model. The career path and expectations of UK GPs has changed considerably over the last forty years. Today's GP is expected to be a member of a wider multi-disciplinary team commissioned to deliver national standards of care and has a role barely recognisable to the one many experienced GPs practising in the 1990's remember, where GP partners tended to stay in one practice for most of their career and there was less regulation and a high expectation of autonomy. In the contemporary career model, GPs said they are expected to give up autonomy in many areas of their job and are expected to accommodate increasing government regulation and bureaucracy, which increases stress related to workload, particularly 'paperwork'/record-keeping.

Factors associated with job satisfaction (shaded orange in Figure 3) are listed; along with factors associated with high job satisfaction on the right (shaded red); and factors associated with low job satisfaction on the left (shaded blue). Job satisfaction appears pivotal to whether a GP will successfully adapt and remain in practice, or will become overwhelmed by external influences and pressures and leave the profession. GPs said job satisfaction directly relates to the quality of the doctor-patient relationship, with more time available for GPs to spend with their patients being associated with better job satisfaction. GPs with high job satisfaction describe feeling supported by good practice relationships, while GPs with low job satisfaction describe low morale and feeling unsupported.

For peer review only

Figure 3 - **Explanatory Model of key factors associated with GP leaving behaviour**

For peer review only

For peer review only - http://bmjopen.bmj.com/site/about/guidelines.xhtml

Some GPs experiencing low job satisfaction report a lack of good practice relationships, and describe working in a 'blame culture' where they fear litigation. Others describe a 'bullying culture', feel undervalued and mistrusted by patients and government, in addition to being inadequately trained in IT, under-resourced, and poorly portrayed in the media. Older GPs or GPs with a more conscientious personality may find it more difficult to adapt, and some GPs describe physical symptoms of fatigue and loss of stamina, e.g. women experiencing sleeplessness due to the menopause. GPs with low job satisfaction appeared more likely to experience reduced feelings of wellbeing, and experience ill- health and burnout. They were also less likely to experience feelings of loyalty to the NHS and more likely to quit (retire, change profession or relocate), exacerbated by a cultural norm of early retirement in the profession. Financial incentives and pension arrangements appeared to be more important to GPs with low job satisfaction and, for some GPs, financial incentives (intended to help retain GPs) may cause them to retire earlier rather than stay in practice longer.

GP shortages (through poor recruitment and retention) and patient demand are creating pressure on full-time GPs, leading some to consider retiring. Patient demands may be higher in areas of higher deprivation and with populations with multiple health and social problems. The impact of GP shortages are most keenly felt in smaller practices, with some GPs feeling trapped between continuing to work full-time under extreme pressure or to retire completely as they fear working part-time would shift the burden of responsibility onto colleagues. The explanatory model shows how this situation is compounded by pressures from increased workload, particularly from increased administration, as well as from secondary care (Figure 3, shaded green). Increased complexity in referral pathways e.g. hospitals providing increasingly specialised services (i.e. shifting more care to primary care) and delays in communication, contribute to GPs' experiencing a depersonalised, fragmented healthcare system. Feelings of uncertainty over the future of general practice are prevalent, with GPs less likely to invest in buildings and make long-term commitments. Younger GPs may be more reluctant to take on partnerships because of the added responsibilities and risks involved. For some, poor relationships between older and younger doctors and/or opposing views about how a practice should be run result in older GPs feeling unsupported, less loyal to the NHS and more likely to leave.

In summary, UK GPs with poor job satisfaction report feeling overworked and unsupported. Combined with changing relationships with patients and interfaces with secondary care, and the gradual sense of loss of control over large parts of the job, many GPs report a reduction in job satisfaction. Lack of time with patients is perceived to compromise the ability to practise patient-centred care and undermines GPs' professional autonomy and values, resulting in further diminished job satisfaction. Once job satisfaction has become negatively impacted, the combined pressures of increased patient demand and workload, together with other stress factors such as poor IT resources, negative media portrayal, poor practice relationships and a "bullying" or "blame" culture, has left many feeling unsupported and vulnerable to burnout and ill health, and, ultimately, to the decision to leave general practice.

For peer review only - http://bmjopen.bmj.com/site/about/guidelines.xhtml

Discussion

The thematic analysis of four qualitative interview studies with UK GPs, two from 2015 and 2016, and two older ones from 2004 and 2005, yielded five overarching types of factors related to GPs leaving or intending to leave direct patient care or reduce their hours, together with more specific sub-themes underlying or linked to these five factors. These themes were categorised into a framework and relationships between identified factors summarised in a visual explanatory model that was developed from them (figure 3). All of these qualitative studies were judged to be of reasonable to good quality.

Overall, the rather negative picture portrayed by the four qualitative interview studies was that UK GPs with poor job satisfaction are also those who feel overworked and unsupported. Many feel part of an over-bureaucratised system, and describe being at the front-end of a service unable to deliver what it promises. Combined with changing relationships with patients and changing interfaces with secondary care, and the gradual sense of loss of control over large parts of the job, many GPs report a reduction in job satisfaction over time. Lack of time with patients is perceived to compromise the ability to practice patient-centred care and continuity of care and, with it, the GPs professional autonomy and values resulting in diminished job satisfaction. Once job satisfaction has become negatively impacted, the combined pressures of increased patient demand and workload together with other stress factors such as poor IT resources, negative media portrayal, poor practice relationships and a perceived “bullying” or “blame” culture has left many feeling unsupported and vulnerable to burnout and ill health. Ultimately, for some this leads to their decision to leave general practice altogether or to substantially reduce their clinical hours.

Our explanatory model (Figure 3) highlights the pivotal role of administrative support in enabling GP flexible working. Both Hutchins et al (8) and Doran et al (11) support this finding, suggesting that additional administrative assistance could enable more time to see patients. Our explanatory model also highlights the complexity of the problem and suggests solutions for retention will not be simple. This is supported by Ipsos MORI (10) who state there can be no ‘silver bullet’ approach to the complex multifactorial issues underlying current disaffection among UK GPs.

Strengths and weaknesses

This systematic review has been conducted and written up with reference to PRISMA guidelines. Potential for transferability is based on stakeholder engagement during the project. Relevant stakeholders were involved in the review; several GPs on the team of co-investigators were involved in the development of the review protocol. Patients were involved through contributing to a Patient and Public Involvement (PPI) workshop where the explanatory model was discussed (Supplementary File).

Limitations include identification of a small number of UK studies. Although a single non-UK study was identified (not reported here), we were not able to translate study findings across countries. In addition, the synthesis of qualitative evidence presented here relates more or less only to NHS General Practice in England. However, it seems likely that many of these factors are generic within primary care in the rest of the UK.

For peer review only - http://bmjopen.bmj.com/site/about/guidelines.xhtml

Conclusions

While recognising the complexity of the current situation, and acknowledging there is unlikely to be a "silver bullet" solution, the synthesis shows an association between flexible working and improved job satisfaction, potentially delaying retirement. GP's views suggest that stress associated with seeing more patients, including more complex patients, but with the same traditional constraints on appointment times, needs to be addressed. Solutions involving alleviating non-clinical administrative burden, e.g. through additional staff resources resulting in more patient-centred care, may be motivating to many GPs.

Funding

The project was funded by the National Institute for Health Research HS&DR programme (project 253 14/196/02). The views and opinions expressed are those of the authors and do not necessarily reflect those of HS&DR programme, the NIHR, the NHS, or the Department of Health.

Competing interests

None, except that two of the included studies were conducted by two of the co-authors of this systematic review (JC and AS) and the principal investigator of the wider ReGROUP study of which this systematic review is a part (JC). Neither AS or JC had any involvement in the detailed data extraction or quality assessment of their studies or any of the other studies. Also, AA has received personal fees from Northern Eastern Western Devon CCG, Devon Local Medical Committee, British Medical Association, University of Exeter, CLAHRC South West Peninsula, and NHS England Medical Directorate (South), outside of this work.

Acknowledgments

We are very grateful to Simon Briscoe and Chris Cooper for their generous support at the earlier stages of planning this review and for supporting the project's information specialist, Sophie Robinson. We thank the rest of the ReGROUP project co-investigators and researchers for their support and useful comments in progress meetings. We would also like to thank all the PPI group members who attended the mid-review PPI workshop and provided valuable feedback and comments on our emerging review findings and explanatory model. SD is also supported by the National Institute for Health Research (NIHR) Collaboration for Leadership in Applied Health.

For peer review only

For peer review only - http://bmjopen.bmj.com/site/about/guidelines.xhtml

References

1. workforce HEEHCoG. Securing the Future GP Workforce - Delivering the Mandate on GP Expansion.; 2014.
2. UoEMS. PCRG. The changing general practitioner workforce: the development of policies and strategies aimed at retaining experienced GPs and those taking a career break in direct patient care: ReGROUP project.; 2016.
3. Wallace A, Croucher K, Quilgars D, Baldwin S. Meeting the challenge: developing systematic reviewing in social policy. Policy Polit. 2004;32(4):455-70.
4. Thomas J, Harden A. Methods for the thematic synthesis of qualitative research in systematic reviews. BMC Med Res Methodol. 2008;8:45.
5. Campbell J, Calitri R, Sansom A. Retaining the experienced GP workforce in Direct Patient Care (ReGROUP) - Final Report for the South West AHSN. Exeter; 2015
6. Sansom A, Calitri R, Carter M, Campbell J. Understanding quit decisions in primary care: a qualitative study of older GPs. BMJ Open. 2016;6(2):e010592.
7. NVivo qualitative data analysis Software. 11 ed: QSR International Pty Ltd. ; 2015.
8. Hutchins A. Influences on GPs' early retirement, and how to keep them. 2005.
9. Newton J. Job dissatisfaction and early retirement : a qualitative study of general practitioners in the Northern Deanery. 2004.
10. Ipsos M. Looking to the future: the recruitment, retention and return of GPs (Summart and next steps report for NHS England). London: Ipsos MORI Social Research Institute; 2015.
11. Doran N, Fox F, Rodham K, Taylor G, Harris M. Lost to the NHS: a mixed methods study of why GPs leave practice early in England. British Journal of General Practice. 2016;66(643):E128-E35.
12. Dwan KM, Douglas KA, Forrest LE. Are "part-time" general practitioners workforce idlers or committed professionals? BMC family practice. 2014;15:154.

For peer review only

For peer review only - http://bmjopen.bmj.com/site/about/guidelines.xhtml

1. Family Practice/ or General Practice/
2. physicians, family/ or physicians, primary care/
3. General Practitioners/
4. Primary Health Care/
5. “primary care”.tw.
6. “general practi$”.tw
7. “family doctor$”.tw.
8. “family physician$”.tw.
9. “family medic$”.tw.
10. (GP or GPs).tw.
11. or/1-10
12. (career$ adj3 (interrupt$ or chang$ or pattern$ or decision$ or leav$ or break$)).tw.
13. (retire$ adj3 (decision$ or medical$ or option$ or choice$ or pattern$ or determin$)).tw.
14. (job$ adj3 (chang$ or leav$)).tw.
15. (work$ adj3 (retention or retain$)).tw.
16. (long adj3 (sick$ or absen$ or ill$)).tw.
17. (burnout or “burn out”).tw.
18. Job Satisfaction/
19. Personnel Turnover/
20. Career Choice/
21. Retirement/
22. or/12-21
23. 11 and 22
24. limit 23 to yr="1990 -Current"

Figure 1 - Medline search strategy

212x151mm (150 x 150 DPI)

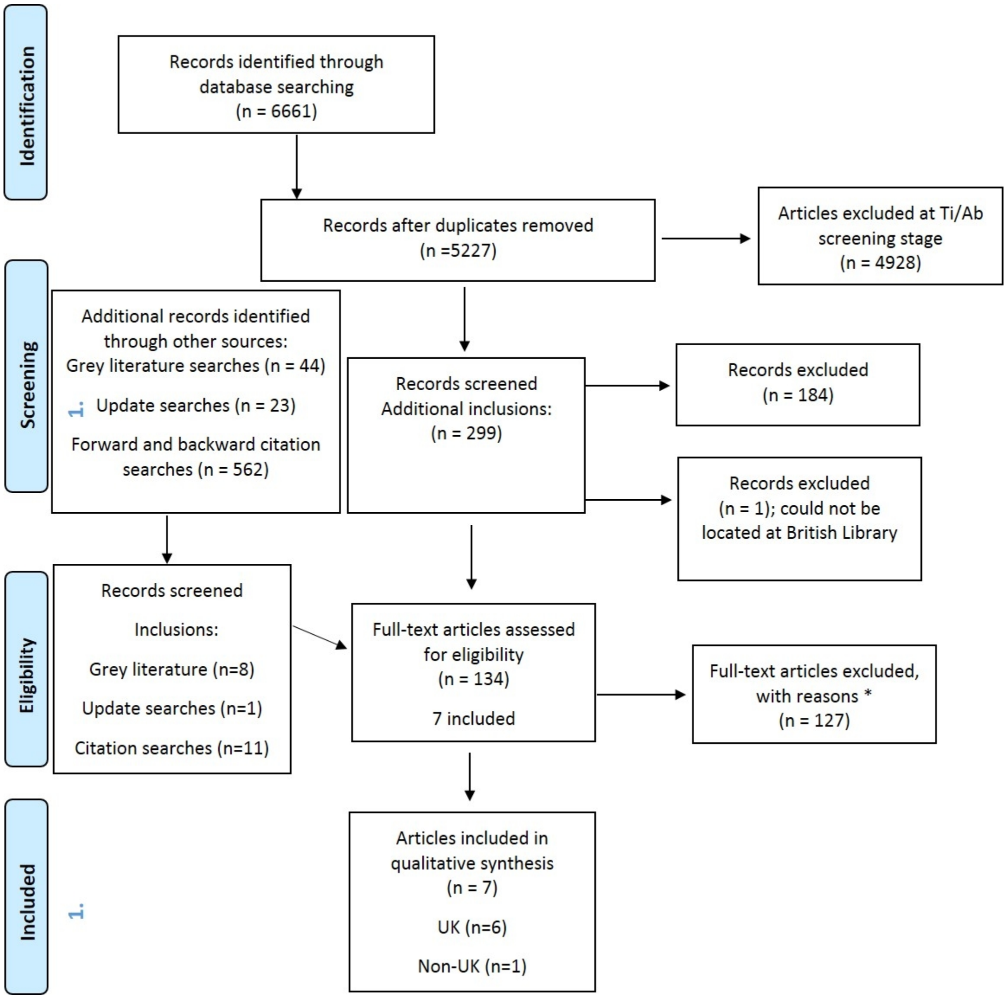

Figure 2 - PRISMA flow diagram showing process of study selection

195x193mm (150 x 150 DPI)

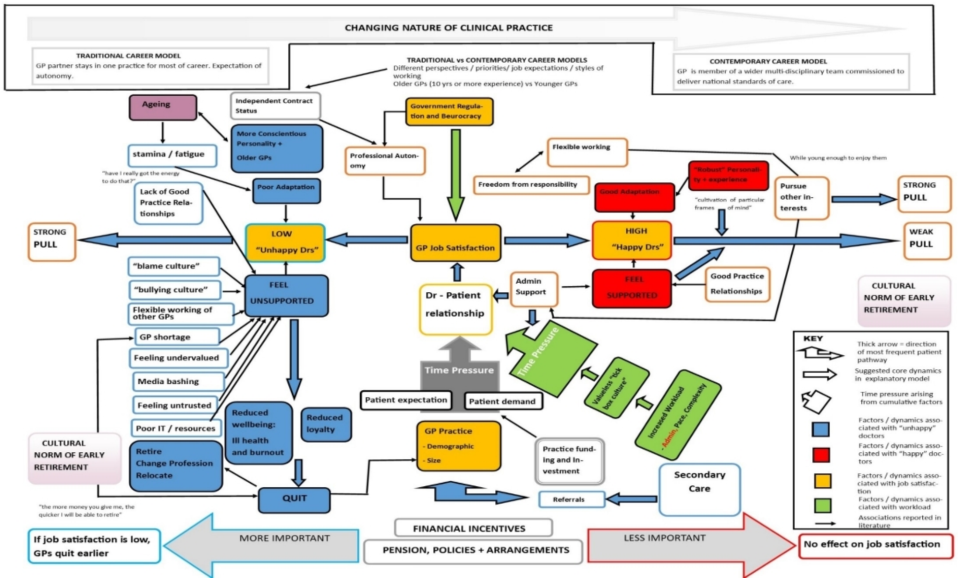

Figure 3 - Explanatory Model of key factors associated with GP leaving behaviour

260x150mm (220 x 220 DPI)

Online Supplementary File

Appendix 1 - Literature search strategies

Database: MEDLINE
Host: Ovid
Data Parameters: 1946 to January Week 3 2016
Date Searched: 29/01/2016
Searcher: SR
Hits: 3655
Strategy:
1. Family Practice/ or General Practice/
2. physicians, family/ or physicians, primary care/
3. General Practitioners/
4. Primary Health Care/
5. "primary care".tw.
6. "general practi$".tw.
7. "family doctor$".tw.
8. "family physician$".tw.
9. "family medic$".tw.
10. (GP or GPs).tw.
11. or/1-10
12. (career$ adj3 (interrupt$ or chang$ or pattern$ or decision$ or leav$ or break$)).tw.
13. (retire$ adj3 (decision$ or medical$ or option$ or choice$ or pattern$ or determin$)).tw.
14. (job$ adj3 (chang$ or leav$)).tw.
15. (work$ adj3 (retention or retain$)).tw.
16. (long adj3 (sick$ or absen$ or ill$)).tw.
17. (burnout or "burn out").tw.
18. Job Satisfaction/
19. Personnel Turnover/
20. Career Choice/
21. Retirement/
22. or/12-21
23. 11 and 22
24. limit 23 to yr="1990 -Current"

For peer review only

Database: MEDLINE(R) In-Process & Other Non-Indexed Citations
Host: Ovid
Data Parameters: 28 January 2016
Date Searched: 28/01/2016
Searcher: SR
Hits: 87
Strategy:
1. "primary care".tw.
2. "general practi$".tw.
3. "family doctor$".tw.
4. "family physician$".tw.
5. "family medic$".tw.
6. (GP or GPs).tw.
7. or/1-6
8. (career$ adj3 (interrupt$ or chang$ or pattern$ or decision$ or leav$ or break$)).tw.
9. (retire$ adj3 (decision$ or medical$ or option$ or choice$ or pattern$ or determin$)).tw.

For peer review only - http://bmjopen.bmj.com/site/about/guidelines.xhtml

10. (job$ adj3 (chang$ or leav$)).tw.
11. (work$ adj3 (retention or retain$)).tw.
12. (long adj3 (sick$ or absen$ or ill$)).tw.
13. (burnout or "burn out").tw.
14. or/8-13
15. 7 and 14

Database: PsycINFO

Host: Ovid
Data Parameters: 1806 to January Week 4 2016
Date Searched: 29/01/2016
Searcher: SR
Hits: 511
Strategy:
1. family medicine/
2. family physicians/
3. general practitioners/
4. primary health care/
5. "primary care".tw.
6. "general practi$".tw.
7. "family doctor$".tw.
8. "family physician$".tw.
9. "family medic$".tw.
10. (GP or GPs).tw.
11. or/1-10
12. (career$ adj3 (interrupt$ or chang$ or pattern$ or decision$ or leav$ or break$)).tw.
13. (retire$ adj3 (decision$ or medical$ or option$ or choice$ or pattern$ or determin$)).tw.
14. (job$ adj3 (chang$ or leav$)).tw.
15. (work$ adj3 (retention or retain$)).tw.
16. (long adj3 (sick$ or absen$ or ill$)).tw.
17. (burnout or "burn out").tw.
18. job satisfaction/
19. employee turnover/
20. occupational choice/
21. retirement/
22. or/12-21
23. 11 and 22
24. limit 23 to yr="1990 -Current"

For peer review only

Database: HMIC (Health Management Information Consortium)

Host: Ovid
Data Parameters: 1979 to November 2015
Date Searched:
Searcher: SR
Hits: 417
Strategy:
1. exp general practice/
2. exp general practitioners/
3. primary care/
4. "primary care".tw.
5. "general practi$".tw.
6. "family doctor$".tw.

For peer review only - http://bmjopen.bmj.com/site/about/guidelines.xhtml

7. "family physician$".tw.
8. "family medic$".tw.
9. (GP or GPs).tw.
10. or/1-9
11. (career$ adj3 (interrupt$ or chang$ or pattern$ or decision$ or leav$ or break$)).tw.
12. (retire$ adj3 (decision$ or medical$ or option$ or choice$ or pattern$ or determin$)).tw.
13. (job$ adj3 (chang$ or leav$)).tw.
14. (work$ adj3 (retention or retain$)).tw.
15. (long adj3 (sick$ or absen$ or ill$)).tw.
16. (burnout or "burn out").tw.
17. job satisfaction/
18. occupational choice/
19. exp retirement/
20. or/11-19
21. 10 and 20
22. limit 21 to yr="1990 -Current"

Database: ASSIA

Host: ProQuest

Data Parameters: n/a

Date Searched: 29/01/2016

Searcher: SR

Hits: 214

Strategy:

1. TI,AB("primary care" OR "general practi*" OR "family doctor*" OR "family physician*" OR "family medic*" OR GP OR GPs) OR SU.EXACT("General practice" OR "General practitioners" OR "Primary health care")
2. TI,AB((career* NEAR/2 (interrupt* OR chang* OR pattern* OR decision* OR leav* OR break*)) OR (retire* NEAR/2 (decision* OR medical* OR option* OR choice* OR pattern* OR determin*)) OR (job* NEAR/2 (chang* OR leav*)) OR (work* NEAR/2 (retention OR retain*)) OR (long NEAR/2 (sick* OR absen* OR ill*) OR (burnout OR "burn out"))) OR SU.EXACT(("Job satisfaction") OR ("Career choice")) OR SU.EXACT.EXPLODE("Early retirement" OR "Mandatory retirement" OR "Retirement")
3. 1 AND 2

Database: Cochrane

Host: Cochrane Collaboration

Data Parameters: CENTRAL: Issue 12 of 12, December 2015; CDSR: Issue 1 of 12, January 2016

Date Searched: 29/01/2016

Searcher: SR

Hits: 75

Strategy:

1 MeSH descriptor: [General Practice] this term only
2 MeSH descriptor: [Family Practice] this term only
3 MeSH descriptor: [Physicians, Family] this term only
4 MeSH descriptor: [Physicians, Primary Care] this term only
5 MeSH descriptor: [General Practitioners] this term only
6 MeSH descriptor: [Primary Health Care] this term only

For peer review only - http://bmjopen.bmj.com/site/about/guidelines.xhtml

7 "primary care":ti or "primary care":ab
8 "general practi*":ti or "general practi*":ab
9 "family doctor*":ti or "family doctor*":ab
10 "family physician*":ti or "family physician*":ab
11 "family medic*":ti or "family medic*":ab
12 (GP or GPs):ti or (GP or GPs):ab
13 (13-#12)
14 (career* near/3 (interrupt* or chang* or pattern* or decision* or leav* or break*)):ti
15 (career* near/3 (interrupt* or chang* or pattern* or decision* or leav* or break*)):ab
16 (retire* near/3 (decision* or medical* or option* or choice* or pattern* or determin*)):ti
17 (retire* near/3 (decision* or medical* or option* or choice* or pattern* or determin*)):ab
18 (job* near/3 (chang* or leav*)):ti
19 (job* near/3 (chang* or leav*)):ab
20 work* near/3 (retention or retain*):ti
21 work* near/3 (retention or retain*):ab
22 long near/3 (sick* or absen* or ill*):ti
23 long near/3 (sick* or absen* or ill*):ab
24 (burnout or "burn out"):ti
25 (burnout or "burn out"):ab
26 MeSH descriptor: [Job Satisfaction] this term only
27 MeSH descriptor: [Personnel Turnover] this term only
28 MeSH descriptor: [Career Choice] this term only
29 MeSH descriptor: [Retirement] this term only
30 (9-#29)
31 #13 and #30

Database: Web of Science
Host: Thomson Reuters
Data Parameters: SCI-EXPANDED and SSCI
Date Searched: 29/01/2016
Searcher: SR
Hits: 1702
Strategy:

1. **TOPIC:** (family (practic* or doctor* or physician* or medic*))
2. **TOPIC:** ("general practi*")
3. **TOPIC:** ("primary care")
4. **TOPIC:** (GP or GPs)
5. 1 OR 2 OR 3 OR 4
6. **TOPIC:** (career near/2 (interrupt* or chang* or pattern* or decision* or leav* or break*))
7. **TOPIC:** (retire* near/2 (decision* or medical* or option* or choice* or pattern* or determin*))
8. **TOPIC:** (job* near/2 (chang* or leav*))
9. **TOPIC:** (work* near/2 (retention or retain*))
10. **TOPIC:** (long near/2 (sick* or absen* or ill*))
11. **TOPIC:** ((burnout or "burn out"))
12. **6 OR 7 OR 8 OR 9 OR 10 OR 11**
13. 5 AND 12
14. Limit to 1990-

For peer review only - http://bmjopen.bmj.com/site/about/guidelines.xhtml

Appendix 2 - List of high-income OECD countries, defined by the World Bank as a country with a gross national income per capita US$12,236 or more in 2016

Australia
Austria
Belgium
Canada
Chile
Czech Republic
Denmark
Estonia
Finland
France
Germany
Greece
Hungary
Iceland
Ireland
Israel
Italy
Japan
Korea, Rep.
Luxembourg
Netherlands
New Zealand
Norway
Poland
Portugal
Slovak Republic
Slovenia
Spain
Sweden
Switzerland
United Kingdom
United States

For peer review only

Appendix 3 - Excluded studies with reasons

	Paper	Reason for exclusion
1	Aseltine RH, Jr., Katz MC. Connecticut physic workforce survey 2008: initial findings on physician perceptions and potential impact on access to medical care. Conn Med. 2008;72(9):539-46.	Not clear whether participants are GPs/PCPs. No examination of factors/associations with/determinants of quitting/intention to quit profession.
2	Aseltine RH, Jr., Katz MC, Geragosian AH. Connecticut physician workforce survey 2009: physician satisfaction, physician supply and patient access to medical care. Conn Med. 2010;74(5):281-91.	No examination of factors/associations with/determinants of quitting/intention to quit profession.
3	Ashworth M., Armstrong D. Sources and implications of dissatisfaction among new GPs the inner city. Family Practice 1999;16(1):18-22.	No examination of factors/associations with/determinants of quitting/intention to quit profession. Career decisions and progression.
4	Baker, M., J. Williams, and R. Petchey, GPs in principle but not in practice: a study of vocationally trained doctors not currently working as principals. BMJ, 1995. 310(6990): 1301-4.	No qualitative data
5	Baker, M., The work commitments of general practitioners: a study of 1986, 1991 and 1996 cohort JCPTGP qualifiers. Monograph series, Nottingham Primary Care Research Unit. 2000 Nottingham: University of Nottingham Division of General Practice. iii,45.	No qualitative data
6	Barnett RC, Gareis KC, Carr PL. Career satisfaction and retention of a sample of wome physicians who work reduced hours. Journal of Womens Health. 2005;14(2):146-53.	Not clear whether are GPs/PCPs.
7	Beasley JW, Karsh BT, Sainfort F, Hagenauer Marchand L. Quality of work life of family [illegible] organizations: a WReN study. Wisconsin Med Jnl. 2004;103(7):51-5.	No examination of factors/associations with/determinants of quitting/intention to quit profession. Turnover between different employers.
8	Beasley JW, Karsh BT, Hagenauer ME, March L, Sainfort F. Quality of work life of independe vs employed family physicians in Wisconsin: a WreN study. Ann Fam Med. 2005;3(6):500	No examination of factors/associations with/determinants of quitting/intention to quit profession. Turnover between different employers.
9	Brett TD, Arnold-Reed DE, Phan CT, Moorhead RG, Hince DA. Work intentions and opinions of general practice registrars. Medical Journal of Australia, 2009; 191 (2):74-7.	No qualitative data
10	British Medical Association. National survey of GPs: the future of General Practice 2015. BMA 2015.	No examination of factors/associations with/determinants of quitting/intention to quit profession.
11	Buchbinder SB, Wilson M, Melick CF, Powe N Primary care physician job satisfaction and turnover. Am J Manag Care. 2001;7(7):701-13	<90% are GPs/PCPs and results for GPs not reported separately.

For peer review only - http://bmjopen.bmj.com/site/about/guidelines.xhtml

		No examination of factors/associations with/determinants of quitting/intention to quit profession.<br>Turnover between different employers.
12	BuddebergFischer B, Stamm M, Buddeberg C Bauer G, Haemmig O, Knecht M, et al. The impact of gender and parenthood on physiciar careers- professional and personal situation seven years after graduation. BMC Health Se Res. 2010;10:10.	<90% are GPs/PCPs and results for GPs not reported separately.<br>No examination of factors/associations with/determinants of quitting/intention to quit profession.
13	Calitri R, Adams A, Atherton H, Reeve J, Hill N Investigating the sustainability of careers in academic primary care: a UK survey. BMC Fa Pract. 2014;15:205.	Not clear whether are GPs/PCPs.<br>No examination of factors/associations with/determinants of quitting/intention to quit profession.
14	Cameron R, Redman S, Burrow S, Young B. Comparison of career patterns of male and female graduates of one Australian medical school. Teaching and Learning in Medicine. 1995;7(4):218-24.	<90% are GPs/PCPs and results for GPs not reported separately.<br>No examination of factors/associations with/determinants of quitting/intention to quit profession.<br>Career decisions and progression.
15	CarrPL, Gareis KC, Barnett RC. Characteristic and outcomes for women physicians who wor reduced hours. Journal of Womens Health & GenderBased Medicine. 2003;12(4):399-405.	Not clear whether are GPs/PCPs.<br>No examination of factors/associations with/determinants of quitting/intention to quit profession.
16	Chambers M, ColthartI andMcKinstryB. Scottish general practitioners' willingness to take part in a post-retirement retention scheme: questionnaire survey. British Medical Journal, 2004. 328(7435): p. 329	No qualitative data
17	CheraghiSohi S, McDonald R, Harrison S, Sanders C. Experience of contractual change UK general practice: a qualitative study of salaried GPs. British Journal of General Pract 2012;62(597):e282-7.	No examination of factors/associations with/determinants of quitting/intention to quit profession.
18	}uu}vÁ ošZ &µv X WŒ]u Œ(views of recent trends in health care delivery and payment: findings from the Commonwealt Fund/Kaiser Family Foundation 2015 national survey of primary care providers. Issue Brief. 2015;24.	<90% are GPs/PCPs and results for GPs not reported separately.
19	Cossman JS. Mississippi's physician labor for current status and future challenges. J Miss State Med Assoc. 2004;45(1):3-31.	Not clear whether are GPs/PCPs.<br>No examination of factors/associations with/determinants of quitting/intention to quit profession.
20	Crouse BJ. Recruitment and retention of famil physicians. Minn Med. 1995;78(10):29-32.	Uses pre1990 data (from 1982 and 1984).
21	Dale J et al. Retaining the general practitioner workforce in England: what matters to GPs? A crosssectional study. BMC Family Practice, 2015. 16(1): p. 140.	No qualitative data

For peer review only - http://bmjopen.bmj.com/site/about/guidelines.xhtml

22	Davidson JM, Lambert TW, Parkhouse J, Eva Goldacre MJ. Retirement intentions of doctors who qualified in the United Kingdom in 1974: Postal questionnaire survey. Journal of Public Health Medicine. 2001;23(4):323	Not clear whether are GPs/PCPs.
23	Degen C, Li J, Angerer P. Physicians' intentio leave direct patient care: An integrative review. Human Resources for Health. 2015;13(1).	Not clear whether are GPs/PCPs.
24	DesRoches CM, Buerhaus P, Dittus RS, Done K. Primary care workforce shortages and care recommendations from practicing clinicians. AcadMed. 2015;90(5):671-7.	No examination of factors/associations with/determinants of quitting/intention to quit profession.<br>Career decisions.
25	Dewa CS, Loong D, Bonato S, Thanh NX, Jac P. How does burnout affect physician productivity? A systematic literature review. BMC Health Services Research. 2014;14(1).	Not clear whether are GPs/PCPs.<br>Burnout but not associated with absence from work.
26	DewaCS, Jacobs P, Xuan Thanh N, Loong D. A estimate of the cost of burnout on early retirement and reduction in clinical hours of practicing physicians in Canada BMC Health Services Research. 2014; 14: 254	No qualitative data
27	Dowell AC, Hamilton S, McLeod DK. Job satisfaction, psychological morbidity and job stress among New Zealand general practitioners. N Z Med J. 2000;113(1113):269-72.	No examination of factors/associations with/determinants of quitting/intention to quit profession.
28	Evans J, Lambert T, and Goldacre M, GP recruitment and retention: a qualitative analys of doctors' comments about training for and working in general practice. Occasional Paper Royal College of General Practitioners, 2002(p. iii-vi, 1-33.	No qualitative data
29	Farber NJ, Bryson C, Collier VU, Weiner JL, E EG. Work enjoyment, intention to discontinue practice,and burnout in primary care physicians. J Gen Intern Med. 2003;18(Supplement 1):240.	Conference abstract only.
30	French F General practitioner non-principals benefit from flexible working. 2005.	No qualitative data
31	French F Why do work patterns differ between men and women GPs? 2006.	No qualitative data
32	Gibson J et al Eighth National GP Worklife Survey UK. 2015.	No qualitative data
33	Gregory ST, Menser T. Burnout Among Prima Care Physicians: A Test of the Areas of Workl Model. J Health Manag. 2015;60(2):133-48.	No examination of factors/associations with/determinants of quitting/intention to quit profession.<br>Burnout but not associated with absence from work.

For peer review only - http://bmjopen.bmj.com/site/about/guidelines.xhtml

34	Hall CB, Brazil K, Wakefield D, Lerer T, Tenne Organizational culture, job satisfaction, and clinician turnover in primary care. J. 2010;1(1):29-36.	No examination of factors/associations with/determinants of quitting/intention to quit profession.<br>Turnover between different employers.
35	Hann M, Reeves D, and Sibbald B. Relationships between job satisfaction, intentions to leave family practice and actually leaving among family physicians in England. European Journ of Public Health, 2011. 21(4): p. 499-503.	No qualitative data
36	Heponiemi T, Kouvonen A, Vänskä J, Halila H, Sinervo T, Kivimäki M, et al. Health, psychoso factors and retirement intentions among Finnis physicians. Occupational Medicine. 2008;58(6):406-12.	Not clear whether are GPs/PCPs.
37	Heponiemi T, Kouvonen A, Vanska J, Halila H Sinervo T, Kivimäki M, et al. Effects of active on call hours on physicians' turnover intentions a well-being. Scandinavian Journal of Work Environment & Health. 2008;34(5):356-63.	<90% are GPs/PCPs and results for GPs not reported separately.<br>No examination of factors/associations with/determinants of quitting/intention to quit profession.<br>Turnover between different employers.
38	Heponiemi T, Kouvonen A, Vänskä J, Halila H Sinervo T, Kivimäki M, et al. The Association o Distress and Sleeping Problems With Physicia Intentions To Change Profession: The Moderating Effect of Job Control. Journal of Occupational Health Psychology. 2009;14(4):365-73.	Not clear whether are GPs/PCPs.
39	Heponiemi T, Kouvonen A, Aalto AM, Elovaini M. Psychosocial factors in GP work: the effects of taking a GP position or leaving GP work. Eu Public Health. 2013;23(3):361-6	Employment change either *from* or *to* general practice.<br>Turnover between different employers.
40	Heponiemi T, Manderbacka K, Vanska J, Elovainio M. Can organizational justice help the retention of general practitioners? Health Policy. 2013;110(1):22-8.	No examination of factors/associations with/determinants of quitting/intention to quit profession.<br>Turnover between different employers.
41	Heponiemi T, Elovainio M, Presseau J, Eccles MP. General practitioners' psychosocial resources, distress, and sickness absence: a study comparing the UK and Finland. Family Practice. 2014;31(3):319-24.	No examination of factors/associations with/determinants of quitting/intention toquit profession.<br>*All* sickness absence included, not necessarily-lo term sickness absence.
42	Hockly A. Could health service reforms make general practitioners ill? Journal of Public Mental Health. 2012;11(2):53-[illegible].	<90% are GPs/PCPs and results for GPs n reported separately.<br>No examination of factors/associations with/determinants of quitting/intention to quit profession.
43	Hojat M, Gonnella JS, Erdmann JB, Veloski J. G. Primary care and non-primary care physicians: a longitudinal study of their similarities, differences, and correlates before, during, and after medical school. Acad Med. 1995;70(1 Suppl):S17-28.	No examination of factors/associations with/determinants of quitting/intention to quit profession.<br>Career decisions.

For peer review only

For peer review only - http://bmjopen.bmj.com/site/about/guidelines.xhtml

44	Hung DY, Rundall TG, Cohen DJ, Tallia AF, Crabtree BF. Productivity and turnover in PCP the role of staff participation in decision-making. Med Care. 2006;44(10):946-51.	<90% are GPs/PCPs and results for GPs not reported separately.<br>No examination of factors/associations with/determinants of quitting/intention to quit profession.<br>Turnover between different employers.
45	Hutchins A. An investigation into the benefits o prolonged study leave undertaken by general practitioners. 2005<br>Hutchins, A., An investigation into the benefits of prolonged study leave undertaken by gener practitioners. 2005.	No qualitative data
46	Jamieson JL, Webber EM, Sivertz KS. Re-entry residency training: opportunities and obstacles Can Fam Physician. 2010;56(6):e222-6	Career decisions and progression.<br>Retraining programmes to change speciality and/or retraining as a GP. Balance of focus unclear.
47	Jewett EA, Brotherton SE, Ruch-Ross H. A national survey of 'inactive' physicians in the United States of America: enticements to reentry. Hum Resour Health. 2011;9:7.	<90% are GPs/PCPs and results for GPs not reported separately.
48	Johnson N. General practice careers: changin experience of men and women vocational trainees between 1974 and 1989. British Jour of General Practice. 1993;43(369):141-5.	No examination of factors/associations with/determinants of quitting/intention to quit profession.
49	Jones L, Fisher T. Workforce trends in genera practice in the UK: results from a longitudinal study of doctors' careers. British Journal of General Practice. 2006;56(523):163-4	No examination of factors/associations with/determinants of quitting/intention to quit profession.<br>Career decisions and progression.
50	Joyce CM, Scott A, Jeon SH, Humphreys J, K G, Witt J, et al. The "medicine in Australia: balancing employment and life (MABEL)" longitudinal survey--protocol and baseline data for a prospective cohort study of Australian doctors' workforce participation. BMC Health Serv Res. 2010;10:50.	No examination of factors/associations with/ determinants of quitting/intention to quit profession.
51	Joyce CM, Wang WC, McDonald HM. Retirem patterns of Australian doctors aged 65 years a older. Australian Health Review. 2015;39(5):582-7.	<90% are GPs/PCPs and results for GPs not reported separately.
52	Karsh BT, Beasley JW, Brown RL. Employed family physician satisfaction and commitment their practice, work group, and health care organization. Health Serv Res. 2010;45(2):457-75.	No examination of factors/associations with/determinants of quitting/intention to quit profession.
53	Kelley ML, Kuluski K, Brownlee K, Snow S. Physician satisfaction and practice intentions i Northwestern Ontario. Can J Rural Med. 2008;13(3):129-35.	Not clear whether are GPs/PCPs.<br>Focus on remote rural retention.
54	Kerstein J, Pauly MV, Hillman A. Primary care physician turnover in HMOs. Health Serv Res. 1994;29(1):17-37.	No examination of factors/associations with/determinants of quitting/intention to quit profession.

For peer review only

		Turnover between different employers.
55	Kilmartin MR, Newell CJ, Line MA. The balanc act: key issues in the lives of women general practitioners in Australia. Med J Aust. 2002;177(2):87-9.	No examination of factors/associations with/determinants of quitting/intention to quit profession.
56	Kirwan M, Armstrong D. Investigation of burnout in a sample of British general practitioners. British Journal of General Practi 1995;45(394):259-60.	No examination of factors/associations with/determinants of quitting/intention to quit profession.<br>Burnout but not associated with absence from work.
57	Kuusio H, Heponiemi T, Sinervo T, Elovainio M Organizational commitment among general practitioners: a cross-sectional study of the role of psychosocial factors. Scand J Prim Health Care. 2010;28(2):108-14.	No examination of factors/associations with/determinants of quitting/intention to quit profession.<br>Turnover between different employers.
58	Kuusio H, Heponiemi T, Vanska J, Aalto AM, Ruskoaho J, Elovainio M. Psychosocial stress factors and intention to leave job: differences between foreign-born and Finnish-born general practitioners. Scand J Public Health. 2013;41(4):405-11.	No examination of factors/associations with/determinants of quitting/intention to quit profession.<br>Turnover between different employers.
59	Langballe EM, Innstrand ST, Aasland OG, Fa E. The Predictive Value of Individual Factors, Work-Related Factors, and Work-Home Interaction on Burnout in Female and Male Physicians: A Longitudinal Study. Stress and Health. 2011;27(1):73-87.	Not clear whether are GPs/PCPs.<br>No examination of factors/associations with/determinants of quitting/intention to quit profession.<br>Burnout but not associated with absence from work.
60	Lawrence J, Poole P. Career and life experien of New Zealand women medical graduates. N Z Med J. 2001;114(1145):537-40.	<90% are GPs/PCPs and results for GPs not reported separately.<br>Career decisions and progression.
61	Leese B, Young R, Sibbald B. GP principals leaving practice in the UK. European Jnl Gen Practice. 2002;8(2):62-8.	No examination of factors/associations with/determinants of quitting/intention to quit profession.<br>Examines leaving GP principal job for another G job, factors for returning.
62	Linzer M, Manwell LB, Williams ES, Bobula JA Brown RL, Varkey AB, et al. Working conditions in primary care: physician reactions and care quality. Ann Intern Med. 2009;151(1):28-36, W6-9.	<90% are GPs/PCPs and results for GPs not reported separately.<br>No examination of factors/associations with/determinants of quitting/intention to quit profession.
63	Lloyd JR, Leese B. Career intentions and preferences of GP registrars in Yorkshire. Br J GP. April 2006:280-2.	No examination of factors/associations with/determinants of quitting/intention to quit profession.<br>Career decisions and progression.
64	Landon BE, Reschovsky JD, Pham HH, Blumenthal D. Leaving medicine: the consequences of physician dissatisfaction. Me Care. 2006;44(3):234-42.	<90% are GPs/PCPs and results for GPs not reported separately.
65	Lorant V, Geerts C, Duchesnes C, Goedhuys J Ryssaert L, Remmen R, et al. Attracting and retaining GPs: a stakeholder survey of prioritie	<90% are GPs/PCPs and results for GPs not reported separately.

For peer review only - http://bmjopen.bmj.com/site/about/guidelines.xhtml

	British Journal of General Practice. 2011;61(588):e411-8.	No examination of factors/associations with/determinants of quitting/intention to quit profession.<br>Retention and recruitment.
66	Luce A et al. What might encourage later retirement among general practitioners? Journ of Management in Medicine, 2002. 16(4/5): p. 303-310.	No qualitative data
	Martin, S., E. Davies, and B. Gershlick, Under ‰ Œ • • µ Œ W t Z š š Z } u u } v Á international survey of general practitioners means for the UK. 2016, The Health Foundati London. p. 37.	No qualitative data
67	Mayorova T, Stevens F, Scherpbier A, van der Velden L, van der Zee J. Gender-related differences in general practice preferences: longitudinal evidence from the Netherlands 1982-2001. Health Policy. 2005;72(1):73-80.	No examination of factors/associations with/determinants of quitting/intention to quit profession.<br>Career decisions.
68	McComb ED. Which psycho-demographic factors predict a doctor's intention to leave Ne Zealand general practice? New Zealand Medi Journal, 2008.121 (1273): p.25-36	No qualitative data
69	McKinstry B et al. The feminization of the medical work force, implications for Scottish primary care: A survey of Scottish general practitioners. BMC Health Services Research, 2006. 6.	No qualitative data
70	Misra-Hebert AD, Kay R, Stoller JK. A review of physician turnover: Rates, causes, and consequences. American Journal of Medical Quality. 2004;19(2):56-66.	Not clear whether are GPs/PCPs.<br>No examination of factors/associations with/determinants of quitting/intention to quit profession.<br>Turnover between different employers.
71	Miedema B, Easley J, Fortin P, Hamilton R, Tatemichi S. Crossing boundaries: family physicians' struggles to protect their private lives. Can Fam Physician. 2009;55(3):286-7.	No examination of factors/associations with/determinants of quitting/intention to quit profession.
72	Miedema B, Hamilton R, Fortin P, Easley J, Tatemichi S. The challenges and rewards of ru family practice in New Brunswick, Canada: lessons for retention. Rural Remote Health. 2009;9(2):1141.	No examination of factors/associations with/determinants of quitting/intention to quit profession.<br>Focus on remote rural retention.
73	Moreno-Jiménez B, Gálvez-Herrer M, Rodríguez Carvajal R, Vergel AIS. A study of physicians' intention to quit: The role of burnout, commitment and difficult doctor-patient interactions. Psicothema. 2012;24(2):263-70.	Not clear whether are GPs/PCPs.
74	Myhre DL, Konkin J, Woloschuk W, Szafran O Hansen C, Crutcher R. Locum practice by rec family medicine graduates. Can Fam Physician. 2010;56(5):e183-90.	No examination of factors/associations with/determinants of quitting/intention to quit profession.<br>Turnover between different employers.

For peer review only - http://bmjopen.bmj.com/site/about/guidelines.xhtml

75	NormanP, Fitter M, Wall TGeneral ‰Œ š]š]}v Œ•[•µ i šsurgery Æ workload. Social Science and Medicine. 1991 33(2). P.16-16	No qualitative data
76	Nugent A, Black N, Parsons B, Smith S, Murp AW. A national census of Irish general practice training programme graduates 1990-1996. Irish Medical Journal. 2003. 96(1) p 10-12	No qualitative data
77	Odom Walker K, Ryan G, Ramey R, Nunez FI Beltran R, Splawn RG, et al. Recruiting and retaining primary care physicians in urban underserved communities: the importance of having a mission to serve. Am J Public Health 2010;100(11):2168-75.	<90% are GPs/PCPs and results for GPs not reported separately.<br>No examination of factors/associations with/determinants of quitting/intention to quit profession.<br>Career decisions and progression.
78	K[< o o Ç &U K[< o o Ç]DvU ŒE]K^ T. A National Census of irish General Practice Training Programme Graduates 1997-2003	No qualitative data
79	Pathman DE, Konrad TR, Williams ES, Schec WE, Linzer M, Douglas J, et al. Physician job satisfaction, dissatisfaction, and turnover. J. Fam Practice. 2002;51(7):593.	Not clear whether are GPs/PCPs.<br>Turnover between different employers.
80	Pedersen AF, Andersen CM, Olesen F, Vedst P. Risk of Burnout in Danish GPs and Explora of Factors Associated with Development of Burnout: A Two-Wave Panel Study. Int Jnl Fam Med. 2013;2013:603713.	No examination of factors/associations with/determinants of quitting/intention to quit profession.<br>Burnout but not associated with absence from work.
81	Plomondon ME, Magid DJ, Steiner JF, MaWhinney S, Gifford BD, Shih SC, et al. Prim care provider turnover and quality in managed care organizations. Am J Manag Care. 2007;13(8):465-72.	Not clear whether are GPs/PCPs.<br>No examination of factors/associations with/determinants of quitting/intentionto quit profession.<br>Turnover between different employers.
82	Pit S and Hansen V. Factors influencing early retirement intentions in Australian rural genera practitioners. Occupational Medicine, 2014; 64, 297-304.	No qualitative data
83	Presseau J, Johnston M, Johnston DW, Elovainic M, Hrisos S, Steen N, et al. Environmental and individual correlates of distress: Testing Karasek's Demand-Control model in 99 primary care clinical environments. British Journal of Health Psychology. 2014;19(2):292-310.	<90% are GPs/PCPs and results for GPs not reported separately.
84	Putnik K, Houkes I. Work related characteristic work-home and home-work interference and burnout among primary healthcare physicians gender perspective in a Serbian context. BMC Public Health. 2011;11:716.	No examination of factors/associations with/determinants of quitting/intention to quit<br>Burnout but not associated with absence from work.
85	Qidwai W, Beasley JW, Gomez-Clavelina FJ. The present status and future role of family doctors a perspective from the International Federatio of Primary Care Research Networks. 2008.	No examination of factors/associations with/determinants of quitting/intention to quit profession.

For peer review only - http://bmjopen.bmj.com/site/about/guidelines.xhtml

86	Rabatin J, Williams E, Baier Manwell L, Schwa MD, Brown RL, Linzer M. Predictors and Outcomes of Burnout in Primary Care Physicians. J Primary Care Community Health 2016;7(1):41-3.	No examination of factors/associations with/determinants of quitting/intention to quit profession. Burnout but not associated with absence from work.
87	Rittenhouse DR, Mertz E, Keane D, Grumbac No exit: An evaluation of measures of physicia attrition. Health Services Research. 2004;39(5):1571-88.	Not clear whether are GPs/PCPs.
88	Ruhe M, Gotler RS, Goodwin MA, Stange KC. Physician and staff turnover in community primary care practice. J Ambulatory Care Manage. 2004;27(3):242.	No examination of factors/associations with/determinants of quitting/intention to quit profession. Turnover between different employers.
89	Savageau JA, Ferguson WJ, Bohlke JL, Cragin O'Connell E. Recruitment and retention of primary care physicians at community health centers: a survey of Massachusetts physicians Health Care Poor Underserved. 2011;22(3):81 35.	<90% are GPs/PCPs and results for GPs not reported separately. No examination of factors/associations with/determinants of quitting/intention to quit profession. Turnover between different employers.
90	Schattner PL, Coman GJ. The stress of metropolitan general practice. Med J Aust. 1998;169(3):133-7.	No examination of factors/associations with/determinants of quitting/intention to quit profession.
91	Schofield DJ, Beard JR. Baby boomer doctors and nurses: demographic change and transitio to retirement. Med J Aust. 2005;183(2):80	Not clear whether are GPs/PCPs. No examination of factors/associations with/determinants of quitting/intention to quit profession.
92	Schofield DJ, Fletcher SL, Callander EJ. Agei medical workforce in Australia-where will the medical educators come from? Hum Resour Health. 2009;7:82.	No examination of factors/associations with/determinants of quitting/intention to quit profession. Workforce planning data.
93	Scott A, Gravelle H, Simoens S, Bojke C, Sibb B. Job satisfaction and quitting intentions: A structural model of British general practitioners British Journal of Industrial Relations. Vol 44, Issue 3, p.519-540	No qualitative data
94	Shaw S, Goplen G, Houston DS. Career chan among Saskatchewan physicians. Can Med Assoc Jnl. 1996;154(7):1085	Not clear whether are GPs/PCPs. No examination of factors/associations with/determinants of quitting/intention to quit profession. Career decisions and progression.
95	Shorer Y, Biderman A, Rabin S, Karni A, Levi Matalon A. Voluntary departure of family physicians from their workplace: A reflective outlook. Israel Journal of Psychiatry and Relat Sciences. 2015;52(2):134-7.	Not clear whether each of four cases described involved leaving general practice. One is about returning to direct patient care. PC emotions around leaving examined not determinants for quitting.
96	Sibbald, B., C. Bojke, and H. Gravelle, Nationa survey of job satisfaction and retirement intentions among general practitioners in England. BMJ, 2003. 326(7379): p. 22.	No qualitative data
97	Shrestha D, Joyce CM. Aspects of work-life balance of Australian general practitioners:	No qualitative data

For peer review only - http://bmjopen.bmj.com/site/about/guidelines.xhtml

	determinants and possible consequences. Australian Journal of primary Health. Vol 17, Issue 1, p.40-47	
98	Simoens, S., A. Scott, and B. Sibbald, Job satisfaction, work-related stress and intentions to quit of Scottish GPS. Scottish Medical Jour 2002. 47(4): p. 80-6.	No qualitative data
99	Simoens S. Job satisfaction, intentions to quit, and the retention of GPs in England and Scotland 2002.	No qualitative data
100	Simon AB, Alonzo AA. The demography, care pattern, and motivation of locum tenens physicians in the United States. J Healthc Manag. 2004;49(6):363-75; discussion 75-6.	Not clear whether are GPs/PCPs.<br>No examination of factors/associations with/determinants of quitting/intention to quit profession.<br>Career decisions and progression.
101	Solberg IB, Ro KI, Aasland O, Gude T, Moum Vaglum P, et al. The impact of change in a doctor's job position: a five-year cohort study of job satisfaction among Norwegian doctors. BM Health Serv Res. 2012;12:41.	<90% are GPs/PCPs and results for GPs not reported separately.<br>No examination of factors/associations with/determinants of quitting/intention to quit profession.<br>Career decisions and progression.
102	Solberg IB, Tómasson K, Aasland O, Tyssen The impact of economic factors on migration considerations among Icelandic specialist doctors: A cross-sectional study. BMC Health Services Research. 2013;13(1).	<90% are GPs/PCPs and results for GPs not reported separately.<br>No examination of factors/associations with/determinants of quitting/intention to quit profession.
103	Soler JK, Yaman H, Esteva M, Dobbs F, Asen RS, Katic M, et al. Burnout in European family doctors: the EGPRN study. Family Practice. 2008;25(4):245-65.	No examination of factors/associations with/determinants of quitting/intention to quit profession.<br>Burnout but not associated with absence from work.
104	Statistical Bulletin. Statistics for general medic practitioners in England: 1994-2004. Department of Health Publications. 2005/02.	No examination of factors/associations with/determinants of quitting/intention to quit profession.
105	Stearns J, Everard KM, Gjerde CL, Stearns M Shore W. Understanding the needs and concerns of senior faculty in academic medici building strategies to maintain this critical resource. Acad Med. 2013;88(12):1927-33.	Not clear whether are GPs/PCPs.<br>Academic medicine.
106	Stevenson AD, Phillips CB, Anderson KJ. Resilience among doctors who work in challenging areas: a qualitative study. British Journal of General Practice. 2011;61(588):e40 10.	No examination of factors/associations with/determinants of quitting/intention to quit profession.
107	Sumanen M, Aine T, Halila H, Heikkila T, Hyppola H, Kujala S, Vanska J, Virjo I, Mattila K. Where have all the good GPs gone - where will they go? Study of Finnish GPs. BMC Family Practi Vol 13, pp 121	No qualitative data
108	Taylor DH, Quayle JA, and Roberts C. Retention of young general practitioners entering the NHS	No qualitative data

For peer review only - http://bmjopen.bmj.com/site/about/guidelines.xhtml

	from 1991-1992. British Journal of General Practice, 1999. 49(441): p. 277-280.	
109	Taylor DH, Jr., Leese B. Recruitment, retentio and time commitment change of general practitioners in England and Wales, 1990-4: a retrospective study. BMJ. 1997;314(7097):180 10.	No examination of factors/associations with/determinants of quitting/intention to quit profession.
110	Taylor DH, Jr., Leese B. General practitioner turnover and migration in England 1990-94. British Journal of General Practice. 1998;48(428):1070-2.	No examination of factors/associations with/determinants of quitting/intention to quit profession.<br>Turnover between different employers.
111	Taylor DH, Esmail A. Retrospective analysis o census data on general practitioners who qualified in South Asia: who will replace them they retire? BMJ. 1999;318:306-10.	No examination of factors/associations with/determinants of quitting/intention to quit profession.<br>Workforce planning.
112	Taylor K, Lambert T, and Goldacre M, Future career plans of a cohort of senior doctors working in the National Health Service. Journa of the Royal Society of Medicine, 2008. 101(4) p. 182-190.	No qualitative data
113	Taylor K, Lambert T, Goldacre M. Future care plans of a cohort of senior doctors working in the National Health Service. Journal of the Ro Society of Medicine. 2008;101(4):182-90.	Not clear whether are GPs/PCPs.<br>Career decisions and progression.
114	Taylor KS, Lambert TW, Goldacre MJ. Career progression and destinations, comparing men and women in the NHS: postal questionnaire surveys. BMJ. 2009;338:b1735.	No examination of factors/associations with/determinants of quitting/intention to quit profession.<br>Career decisions and progression.
115	Taylor K, Lambert T, Goldacre M. Career destinations, views and future plans of the UK medical qualifiers of 1988. Journal of the Roya Society of Medicine. 2010;103(1):21-30.	<90% are GPs/PCPs and results for GPs not reported separately.<br>No examination of factors/associations with/determinants of quitting/intention to quit profession.<br>Career decisions and progression.
116	The Royal New Zealand College of General Practitioners. 2015 Workforce Survey	No qualitative data
117	Thommasen HV, Lavanchy M, Connelly I, Berkowitz J, Grzybowski S. Mental health, job satisfaction, and intention to relocate. Opinion of physicians in rural British Columbia. Can Fa Physician. 2001;47:737-44.	Not clear whether are GPs/PCPs.<br>Focus on remote rural retention.<br>Burnout but not associated with absence from work.
118	Thornett A, Cobb S, Chambers R, Mohanna K Accessing careers support in primary care. Education for Primary Care. 2005;16(1):63-6	Not clear whether are GPs/PCPs.<br>Career decisions and progression.
119	Toyry S, Kalimo R, Aarimaa M, Juntunen J, Seu M, Rasanen K. Children and work-related stress among physicians. Stress and Health. 2004;20(4):213-21.	Not clear whether are GPs/PCPs.<br>No examination of factors/associations with/determinants of quitting/intention to quit profession.
120	Van Greuningen M, Heiligers PJ, Van der Vel LF. Motives for early retirement of self-employed GPs in the Netherlands: a comparis	No qualitative data

16

For peer review only - http://bmjopen.bmj.com/site/about/guidelines.xhtml

	of two time periods. BMC Health Services Research. Vol 12, p.467	
121	Virtanen P, Oksanen T, Kivimaki M, Virtanen M, Pentti J, Vahtera J. Work stress and health in primary health care physicians and hospital physicians. Occup Environ Med. 2008;65(5):3 6.	No examination of factors/associations with/determinants of quitting/intention to quit profession.<br>Examines differences between GPs and consultants not factors leading to long term sickness.
122	Wainer J. Work of female rural doctors. Aust J Rural Health. 2004;12(2):49-53.	No examination of factors/associations with/determinants of quitting/intention to quit profession.
123	Woodward CA, Ferrier B, Cohen M, Brown J. W Œ } (• •] } v o š] À] š Ç X , } Á] work time changing? Canadian Family Physic Volume 47, p.1414-21	No qualitative data
124	Wordsworth S, Skatun D, Scott A, Firth F. Preferences for general practice jobs: a survey of principals and sessional GPs. British Journa General Practice. 2004;54(507):740	No examination of factors/associations with/determinants of quitting/intention to quit profession.<br>Career decisions and progression.
125	Xu G, Veloski JJ, Hojat M, Fields SK. Physicia intention to stay in or leave primary care specialties and variables associated with such intention. Eval Health Prof. 1995;18(1):92-102.	<90% are GPs/PCPs and results for GPs not reported separately.<br>No examination of factors/associations with/determinants of quitting/intention to quit profession.
126	Young, R., B. Leese, and B. Sibbald, Imbalanc in the GP labour market in the UK: Evidence from a postal survey and interviews with GP leavers. Work, Employment and Society, 2001 15(4): p. 699-719.	No qualitative data
127	Croft, M. (2016). "First 5's in Cornwall - what are their intentions and what influences their career choices?" (Unpublished)	Unpublished (Survey conducted by a GP Academic Trainee research project)

For peer review only

Appendix 4 - Results of quality assessment

	Newton 2004	Hutchins 2005	Campbell 2015	Sansom 201	Doran 2016	Dwan 2014	Ipsos MORI, 2015
1) Is the research question clear?	Y	Y	Y	Y	Y	Y	Y
2) Is the theoretical or ideological perspective of the author (or funder) explicit?	N	N	N	N	N	Y	N
2b) Has this influenced the study design, methods or research findings?	CT	CT	CT	CT	CT	N	CT
3) Is the study design appropriate to answer the question?	Y	Y	Y	Y	Y	Y	Y
4) Is the context or setting adequately described?	N	N	Y	Y	Y	Y	N
5) Is the sample adequate to explore the range of subjects and settings, and ha it been drawn from an appropriate population?	CT	Y	Y	Y	Y	Y	Y
6) Was the data collection adequately described?	Y	N	Y	Y	Y	N	N
7) Was data collection rigorously conducted to ensure confidence in the findings?	CT	CT	Y	Y	Y	Y	CT
8) Was there evidence that the data analysis was rigorously conducted to ensure confidence in the findings?	Y	Y	Y	Y	Y	Y	N
9) Are the findings substantiated by the data?	Y	Y	Y	Y	Y	Y	CT
10) Has consideration been given to any limitations of the methods or data tha may have affected the results?	N	Y	Y	Y	Y	Y	N
11) Do any claims to generalisability follov logically and theoretically from the data?	Y	N	Y	Y	Y	Y	CT
12) Have ethical issues been addressed a confidentiality respected?	CT	Y	Y	Y	Y	Y	Y
13) Is/are the author/s reflexive?	N	N	N	N	N	N	N

[illegible] ublished by Wallace et al (3)

For peer review only - http://bmjopen.bmj.com/site/about/guidelines.xhtml

Appendix 5 - Textual thematic analysis

Undoable / Unmanageable

Workload (administration)

All six UK semi- [illegible]

GPs in one study describe often working 12 or more hours per day, and that this was having a significant impact on their ability to do their role and live their lives (10). GPs describe increased administration, both non-clinical and associated with secondary care, preparing for Care Quality Commission (CQC) visits, management targets, regulations and guidelines (5). This caused stress and reduced job satisfaction and was a factor in GPs decisions to leave practice early. Many GPs who had continued in practice beyond the age of sixty had done so because they had been able to delegate paperwork. Alleviation of administration emerged as a high priority for GPs (8).

Pressures

All six UK semi-struct [illegible]

Fear of making mistakes

Time pressure and conflicting priorities meant that some interviewed GPs felt that the care they were giving was sub-standard, leading to disillusionment and a raised anxiety about the risk of making a mistake.

Patient demands

In one study, GPs said demand for patient care was outstripping supply. Contributing factors cited included unrealistic patient expectations arising from patient access to online information about their symptoms while simultaneously being less willing to treat themselves (10). Others describe an increase in the number of patient contacts without a corresponding increase in the number of GPs; and additional workload from secondary care (6).

The pace and complexity of work was felt to be difficult to maintain. GPs felt patient demands may be higher if GP practices were situated in areas of higher deprivation where populations may have many have multiple health and social problems, or in areas with elderly populations with multiple morbidities and social care needs (5) or in areas with high numbers of asylum seekers (8).

[illegible] not so enjoyable now is that actu [illegible] (Hutchinson 2005) [illegible] (8).

Practice demands (GP shortages and others working reduced hours)

GPs in smaller practices were reported to be more likely to feel trapped between continuing to work full-time under extreme pressure in order to support colleagues, or to retire completely. However, difficulty in recruiting locums precluded many from working part-time. In an unsupportive practice [illegible] health, or early retirement contributed to feelings of burden and stress. In contrast, in more supportive practices, it was felt that such scenarios are better managed by the team (5).

Training and resources

For peer review only - http://bmjopen.bmj.com/site/about/guidelines.xhtml

GPs report feeling placed in a stressful situation of trying to meet raised patient expectations with insufficient resources and with increased workload being compounded by inadequate training and information technology resources, and thought this may particularly impact older GPs experiencing reductions in stamina and physical limitations. Deteriorating eyesight was noted by three GPs in one study (5), however, computer systems seemed unable to accommodate accessibility issues such as the need for a larger font or fewer icons on the screen.

Morale

Identity / Perceived value of GP work

Five UK semi-structured interview studies (5, 6, 8, 10, 11) [illegible]

GPs describe feeling unvalued by both patients and politicians. One GP described feeling how increased patient demand coupled with GP shortages resulted in the perception of the NHS as a [illegible] (Campbell et al 2015) (5).

Professional Culture

Five UK semi-structured interview studies (5, 6, 8, 10, 11) [illegible]

Acceptability of early retirement

GPs report feeling that it is common and acceptable amongst their peers to consider and financially plan to take early retirement and, with this in mind, many GPs have made long-term financial plans to make this happen.

Cultural shift

Authors of one study (9) describe GPs trained for a traditional model of general practice who may struggle to adapt to the current one which sees the GP as one member of a multidisciplinary team commissioned to deliver national standards of care. The introduction of payment-related [illegible] general [illegible] The government has bred a conniving species of GP ... To an extent you do [illegible] longer got any incentive to do anything more than that [illegible] (Hutchins 2005) (8).

Bullying top-down culture

[illegible]

[illegible] then flows all the way down to whoever your locality middle-[illegible] (Doran et al, 2016) (11).

Lone working

GPs said that an unintended consequence of having longer and more intense working days was the limited contact with colleagues and sense of isolation that this could cause. This impacted on practice culture of family practices that had traditionally generated positive and supportive work environments (10). [illegible] leave are based more on self-survival than what is best for the practice.

For peer review only - http://bmjopen.bmj.com/site/about/guidelines.xhtml

Lack of support

Five UK semi-structured interview studies (5, 6, 9-11) }v šŒ] µš š} šZ šZ u ^o l }(•µ‰

Government / political

GPs thought more is expected of GPs with lower financial resources and less support in place. Some 'W• • Œ]]vP ^ š šZ (Œ}vš v }(• ŒÀ] µ (Newton, š} o]À Œ Á 2004) (9). GPs describe organisational changes resulting in a clash of values and diminishing professional autonomy, as health care became more centralised, standardised, and depersonalised.

Negative media portrayal

Some GPs felt misrepresented by the media and felt frustrated that the more positive aspects of their hard work and professionalism went largely unreported. Being the subject of an ongoing and negative media campaign left many feeling undermined and demoralised:

Zt Á Œ š ŒP š]v }u‰o š oÇ µv•Çu‰ šZ š] o]PZš €Y• Á]šZ}µ profession we gave to the public really and it did, over time, become very wearing (Doran et al., 2016) (11).

Job Satisfaction

Five UK semi-structured interview Œ š] o • }všŒ] µš š} šZ šZ u (5, 6, 8, 9, }i • š]•(11).

Job satisfaction was stated to be a major factor in determining the retirement plans of GPs.

Doran et al report GPs in their study, particularly those with 10 years or more practice experience, feeling their job was not meeting their expectations and there was a loss of intellectual challenge. Many GPs felt the level of satisfaction they were able to derive from general practice had declined considerably as a result of increased government regulation and bureaucratic pressure.

/v •}u • •U 'W• • Œ] Z}Á šZ Ç PŒ Á š} Z š šZ]Œ i} U }Œ Z š One former GP described:

ZW ••]}v š oÇ }Œ]vP uÇ Á}Œl v uÇ ‰ š] vš•U / u vU Œ o oÇ / Absolutely loved the actual job, but everything around the (Doran et al., 2016) (11).

Wellbeing

All six UK semi-structured interview articles }vš Œ] µš š} šZ šZ u }(^Á o]vP_X

Many GPs describe themselves as being near burnout (10). Feelings of being overwhelmed, stressed, and losing confidence were also mentioned. One GP described the vicious circle of doctors getting sick, this placing increased pressure on the remaining doctors, who then themselves get sick (5). Time pressure was cited as a factor for GPs not addressing their own health needs:

Looking after their own well-]vP Á • Zi µ•š }v u}Œ šZ]vP š} .š]v[U v 'W• Á šZ]Œ }Áv } š}Œ µ š} v}š Á všš]vP š} Zvµ]• v ‰ š] vš[v v through šZ • u •µ((Œ]vP • Ç}µ, 2016) (6). ^ v•}u š oX

GP burnout also has implications for the quality of patient care, as described by a GP appraiser:

For peer review only - http://bmjopen.bmj.com/site/about/guidelines.xhtml

[illegible] was this disassociation, there was this lack of will to fight to get what patients needed [illegible] (Doran et al., 2016) (11).

Such impacts on the quality of care and the experience of providing care may in turn reinforce patient dissatisfaction and further lower job satisfaction.

Work-life balance

[illegible] (5, 6, 8, 9, 11). [illegible]

Issues relating to quality of working life, rather than increased remuneration, emerged as one of the most important factors influencing retention. GPs of both genders wished to adjust their working hours and planned retirement to spend more time with partners and family in the UK. Many stated that the provision of part-time work within their practices was important to enable retention beyond retirement to reduce the pressure of work for that individual, and to enable them to pursue interests they enjoyed. GPs with high job satisfaction said that although they like their job, they felt it encroached on their lives outside work and that they wanted to enjoy hobbies and other interests whilst they were young enough to do so.

Impact of Organisational Changes

Referral volume and complexity

Five UK semi-structured interview studies (5, 6, 8, 10, 11) [illegible]

GPs report changes to referral systems resulting in a shift in work load from hospital to primary care, combined with changes in patient demographics and demand. Patient pathways are perceived to be more complex and time-[illegible] doctors lacking [illegible] fragmentation and a depersonalised healthcare system (5).

Targets and assessments

Five UK semi-structured interview studies (5, 6, 8, 10, 11) [illegible]

GPs report feeling that management targets, regulations and guidelines increased workload burden (paperwork and bureaucracy) and contribute to stress and loss of job satisfaction. Introduction of [illegible] impacted adversely on the doctor-patient relationship.

[illegible] You spent more time ticking boxes than you did talking to the patient [illegible] stress on me and I felt it affected my rapport with the patient [illegible] (Doran et al., 2016) (11).

Such monitoring and targets were reported by some older GPs as reflecting a lack of trust and [illegible]

Doctor-patient relationship

All six UK semi-[illegible]-patient [illegible]

For peer review only - http://bmjopen.bmj.com/site/about/guidelines.xhtml

[illegible] patient [illegible] relationship. Lack of time with patients meant the ability to practise patient-centred care and continuity of care was perceived to be com[illegible] values were felt to be undermined, resulting in diminished job satisfaction for GPs and diminished satisfaction for patients.

Changing role

All six UK semi-structured interview studies contributed to [illegible]

Responsibility

GPs reported feeling that an increase in responsibility alongside organisational changes had [illegible] cases were getting more complicated, more was being transferred from the responsibility of the hospital to [illegible] administrative things and less and less time being able to devote my mental attention to the patients in front of me[(Doran et al., 2016) (11).

Non-clinical work

[illegible] (11). The GMS contract (2004) was seen to have exacerbated this diminution in role. GPs who continued to practice beyond retirement age had often done so because they had been able to delegate their paperwork, leaving more time for patient consultation, the aspect of general practice they enjoyed.

Rate of change

[illegible]

Many GPs describe becoming progressively worn down by change over a time period, which several of them said had started in 1990 (9) and that this contributed to low morale. Moreover, difficulties were experienced with perceiving the value of changes, many of which were felt to have been made with no long term visio[illegible] and older GPs might be less able to adapt and cope with change, and that tolerance to change diminished the longer a GP has been in practice.

Autonomy and Control

Five UK semi-[illegible]

GPs described how increased government regulation and bureaucratic pressure has led many GPs to feel an erosion of autonomy and professional control, impacting job satisfaction.

Reaccreditation

Two UK semi-structured interview studies (one ten years older than the other) (6, 8) contributed to [illegible] system. Some found appraisals valuable and helpful and highlighted areas to strengthen through professional development, while others felt they were an additional burden and ineffective (6) Some GPs felt strongly that they should not be exempt from re-accreditation if they continue to work beyond retirement age to ensure competence. However, other GPs mentioned that they would schedule their retirement earlier to avoid their next revalidation.

For peer review only - http://bmjopen.bmj.com/site/about/guidelines.xhtml

Projected Future

Viability of early retirement

Three UK semi-structured interview studies (5, 6, 9) v š Œ] µ š š} šZ šZ u ^À]]o]šÇ }(Œ š]Œ u vš_ X

Cultural norms of early retirement coupled with good pension provision appear to encourage part-time working and early retirement for GPs in the UK. The 1995 section of the NHS Pension Scheme and so- o o - Z}µ Œ Œ š]Œ u vš[Á Œ]š achieve early retirement (and/or reducing hours) whilst still receiving an adequate income.

GPs with low job satisfaction reported being more likely to plan to leave as soon as they were financially able. For this dissatisfied group, no manner of practical incentives or inducements would keep them at work:

Z the more money you gave me the quI Œ / Á}µo (Newton 2004) (9). [

Ageing

Four UK semi-structured interview studies (5, 6, 8, 11) v š Œ] µ š š} šZ šZ u }(^ P]vP_ X

Cognitive deterioration and fear of incompetency

Some GPs described how cognitive and physical limitations (e.g. deteriorating eye sight) experienced • šZ Ç P}š }o Œ P À Œ]• š} (o]vP• }(vÆ] šÇ v o I }(}v(]]v }u‰ š v _X ^}u Á Œ }v Œv šZ š šZ]Œ ‰}}Œ Œ u u}ŒÇ }µo to keep up to date. Some GPs recognised their memory and capacity for learning was declining, and said that they would not want to continue in practice if their capacities were inadequate (8).

Resilience

GPs describe feeling that as you get older and stamina decreases, the length of the day is very exhausting and this ca]u‰ š }v 'W•[}v(] v v]o]šÇ v U }v• ‹µ všoÇ capacity to continue working in direct patient care.

Z There seems to be something that happens when you reach about 55: you start to get feelings of struggling with the work and 60 o• v Á(µo o}v P[(GP interviewee) X [Campbell et al., 2015) (5).

Feelings of tiredness may be compounded for some female GPs who may experience sleep disturbance during the menopause (6).

Investment and commitment

Five UK semi-šŒµ šµŒ]vš ŒÀ] Á •šµ] • }všŒ] µš š} šZ šZ u }(^]v }uu]šu v (5, 6, 8, 9, 11).

Partnership issues

GPs reported that poor relationships between older and younger partners arising from differences in values or perspectives could lead to opposing views about how the practice should be run.

ZY]š Z Œ Z šZ ‰}]vš ÁZ Œ Á Z Ç}µvP v Á u u Œ• ÁZ}U (}Œ th]Œ ‰Œ}š š š]u µš Z v[š šZ}µPZš šZŒ}µPZ šZ]u‰ š šZ š You reach a crossroads that says; Z vP }vU / v[š u}‰ šZ]• (9) ‰[[~E Áš}vU îìì ð•

For peer review only - http://bmjopen.bmj.com/site/about/guidelines.xhtml

Such tensions resulted in GPs feeling unsupported, less loyal to their practice and having a decreased likelihood of staying on (6). Practice-level changes, such as peers retiring, could also contribute to decisions to leave:

Ž[À iµ•š Z šZŒ u}Œ Œ š]Œ u vš• •} v ŒoÇ oo šZ ‰ }‰o ÁZ have now gone and been super[illegible] Ç Ç}µvP ŒU]((Œ vš 'W• Y uÇ Á}ŒI • š]•(think a large part of it is because of the changing style of work: the newer doctors work differently. }v[š oI šZ Á [illegible] (GP interviewee in Sansom et al., 2016) (6)

Long-term responsibility

Concerns were evident, of current difficulties of recruiting new partners to a GP practice to replace a retired GP partner (6). However, GPs reported that younger GPs may be reluctant to take on partnerships because of the added risks and responsibilities involved.

Financial investment

GPs reported that concern about the future of general practice meant they may be less likely to invest in buildings and make long term commitments.

Multiple Options and Strategies

Flexible working / Reducing working hours

Five UK semi-structured interview studies (5, 6, 8-10) [illegible] }všŒ] µš š} šZ šZ u ^(o Æ] o Á Œ µ]vP Á}ŒI]vP Z}µŒ•_X

GPs report that while flexible working can bring benefits to individual GPs (young and old), it can increase workload for other GPs if there is difficulty recruiting other partners or locum GPs. This pressure is more keenly felt in smaller practices, with GPs more likely to feel trapped between continuing to work full-time under extreme pressure in order to support colleagues, or to retire completely.

Continue and cope

Four semi-structured interview studies (5, 6, 9, 11) [illegible] }všŒ] µš š} šZ šZ u ^ }vš]vµ v

'W• Œ ‰}Œš šZ [illegible] see their working situation improving and they vary in their ability to cope (5). GPs said resilience to change, or ability to adapt, may be linked to personality type; one GP • Œ] •]vP v Æ‰ Œ] v 'W Á]šZ ^Œ} µ•š_ ‰ Œ•}v o]šÇ v u]v _ ÁZ]o v}šZ Œ š oI• }µš Z À]vP ^ v v}Œu}µ• u}µvš }(Æ‰ ‰ Œ•}v [illegible] (5, 9). Practical coping strategies employed by GPs include looking at work emails from home or in non-work time to try and stay up to date (5), staying late at work, taking work home or changing their appointment times (11). Support given through good working relationships within a GP practice were cited as important for helping GPs cope.

ŽW }‰o Œ Á Œ }(}šZ [illegible] we work together as a group and I think it is a very •µ‰‰}Œš]À ‰Œ š] Y / }v[š šZ]vI /[•š]oo]v šZ E,^](/ Á • probably wou[illegible] Z À o (š Ç Œ• P} šµ oo [illegible] (6) ~^ v•}u š oXU îìíò•

Alternative roles for GPs

All six UK semi-šŒµ šµŒ]vš ŒÀ] Á •šµ] • }všŒ] µš š} šZ šZ u }(^

For peer review only

For peer review only - http://bmjopen.bmj.com/site/about/guidelines.xhtml

New professional roles / extended roles

In one study, two GPs reported completing further training in order to leave general practice; one to become a full-time holistic therapist, while the other intended to work part-time as a complementary therapist (5).

Skills transfer

Alternative job roles mentioned by GPs, that used skills transferable from working as a GP, included appraiser, Clinical Commissioning Group lead, advisory committee member, pharmaceutical consultancy work and working for a medical school.

A medical degree is one of the most wid[illegible] communication, empathy, organisation, managemen[illegible] (5). [illegible]

Professional development / specialisation

One study proposed that for younger GPs, having a medical specialism was thought to provide greater flexibility towards retirement and doctors who already worked part-time in specialist areas outside general practice intended to work entirely in the speciality when they retired (8). Other [illegible] Compensation Appeal Panel Tribunals or DSS (Department for Social Services) Tribunals (9). Others had combined working as a GP with other jobs, such as teaching, to have a more portfolio career (10).

Relocation

Changing jobs (to other medical jobs outside general practice) and relocating abroad were reported in one study to account for some GPs leaving UK general practice (11).

For peer review only

Appendix 6 - Australian case study of part-time working

This section separately presents findings of the only included qualitative interview study that was with GPs outside the UK (12). As well being conducted in a different context to the UK studies, the [illegible] part-time (in this study, six or fewer sessions per week).

Flexible working

[illegible] the changing nature of clinical practice, where they were required to work with more complex patients, often with chronic conditions and associated psychological symptoms. Many of the GPs in this study felt that a mix of clinical, non-clinical and unpaid activities attenuated the tiredness one might otherwise feel when working with such patients and allowed more time for being conscientious e.g. reviewing all the patient records before writing a complex referral and providing [illegible] patients.

Concerns about working flexibly included remuneration, which was considered modest. Also, several GPs found it slightly more difficult to keep up to date clinically.

Continue and cope

The Australian study may offer a different perspective on why some GPs find it easier to cope and continue in the system. One GP suggests that GPs able to adapt to the changing health system may only be able to do so because [illegible] you are doing general practice well clinically, it is quite challenging. I have seen a lot [illegible] (12)

Alternative roles

All of the Australian sessional GPs interviewed were in full-time paid employment in health related areas, including education and training, policy, research and academia and medical specialities. All of [illegible] with flexible work practices.

Doctor-patient relationship

The authors report that the majority of sessional GPs in the study acknowledged increased complexity in treating patients, with a perceived shift away from [illegible] acute and chronic care, towards the management of multiple, chronic diseases.

Patient demands

Australian GPs reported that the prevalence of complex, chronic illness and the increasing need for psychological management meant that consultations were [illegible] might happen if you do take antibiotics? What might happen [illegible] are] the reasons for taking it? [What are] the reasons not for taking it, you know? I think that takes up a lot of time and/ [illegible] (12).

For peer review only - http://bmjopen.bmj.com/site/about/guidelines.xhtml

Lack of support

Lack of perceived support towards GPs from the media appears not to be limited to the UK. Australian media portrayal of sessional GPs was reported to be also critical, suggesting that GP working less than full-time reflected a lack of commitment and that sessional clinical practice is a personal indulgence that disregards the needs of the community.

Job satisfaction

In this study, many of the GPs reported feeling that full-time general practice did not allow them to be the best GP they could be.

[Like] most GPs I want to do a decent job, and I actually always found that if I go beyond a certain vµu Œ }(• ••]}v• / }v[š šZ]vl / u }]vP vš i(12) vÇu}Œ [~ Á v

Wellbeing

Similar dynamics in wellbeing experienced by UK GPs were expressed by sessional GPs in Australia.

The strain of full-š]u o]v] o ‰Œ š] Á • Œ ‰}Œš š} •šŒ}vPoÇ]v(oµ v decisions to work part-time. Sessional clinical practice Á • • v š} }((Œ ^ }Ávš]u _U šZ }‰‰}Œšµv]šÇ š} ^Œ ZŒP Ç}µŒ ššŒ] •_X /š l ‰š šZ u ^(Œ •Z v oo}Á 'W• š} ^u]vš]v P}} u vš o v ‰ZÇ•] o Z ošZ_X dZ Œ that a mix of clinical, non-clinical and unpaid activities attenuated the tiredness one might otherwise feel in full-time clinical practice.

Work-life balance

Cultural influences on work-life balance may be particularly strong. In UK studies, there was no clear gender bias reported for GPs choosing to work less than full-time, with Hutchins et al (8) reporting that GPs of both genders wished to adjust their working hours. However, in this Australian study, the µšZ}Œ• •µPP •š šZ š P v Œ •šŒ}vPoÇ]v(oµ v (u o ‰ Œš]]‰ time. Thirteen female GPs and one male GP had dependent children, but only the man did not mention his children or family during the interview. Three of the mothers commented that their •‰}µ• [• u‰o}Çu vš Œ ‹µ]Œ Z Œ š} Á}Œl sessionally in order to manage the household and caring responsibilities. A further two women with adult children had significant caring responsibilities

For peer review only

For peer review only - http://bmjopen.bmj.com/site/about/guidelines.xhtml

Appendix 7: Summary of patient involvement in thematic analysis and explanatory model

The following Patient Involvement discussion points provided colloquial real world perspectives that contextualised our understanding of our literature-based thematic analysis and associated explanatory model

Flexible Working

While flexible working can bring benefits to individual GPs (young and old) such as freedom from paper work and freedom to pursue other interests, it can increase workload for other practice GPs if they have difficulty recruiting other partner GPs or locums. Discussion with our PPI group suggested that flexible working can have a potentially negative effect on patients who seek appointments with the same GP that they know and have built history and rapport with. If they are consistently inaccessible to them because of their flexible working patterns, patients may experience grief at the loss of the relationship. This could have implications for the NHS as there may be more referrals to secondary care as a consequence. In such circumstances, it is often more acceptable to the patient if the GP retires as this is a predictable and understandable reason for the end of the doctor-patient relationship.

While increasing the availability of locums may relieve pressure on full-time GPs and aid retention of salaried GPs / partners, there was concern from the PPI group that GPs who preferred to travel between GP practices working as locums may choose to do so because it means that they avoid building Doctor - patient relationships. Different personalities may suit different working styles, with permanent salaried GPs / partners having different values and personalities to locums and perhaps valuing the doctor-patient relationship higher.

Continue and Cope

While GPs talk in the semi-structured interviews about strategies that help them to cope with increasing workload and pressures, members of the PPI group note that there is no mention of [illegible]-using alcohol drugs and no mention of GP use of anti-depressants. There is also no reporting of GPs accessing counselling services in the interviews.

Viability of Early Retirement

The PPI group expressed the view that the GP Cultural norm of acceptability of early retirement may be compounded further by GPs expert knowledge about the human body. Because GPs are more able to predict expected deterioration with age, they may be more likely to plan for early retirement when so that they can physically do the things they enjoy.

Ageing

The PPI group noted that holiday entitlement is not mentioned in any of the GP interviews and suggested increased holiday entitlement for aging GPs may help GPs manage their natural fatigue and ultimately improve retention.

Partnership Issues

The qualitative synthesis and explanatory model in this review highlights the importance of good practice relationships for GP retention. When these are not in place, GPs can experience a lack of support which may lead to quitting. The PPI group note that different GPs with different

For peer review only - http://bmjopen.bmj.com/site/about/guidelines.xhtml

personalities / values / working styles may experience conflict when working together in the sam practice. PPI members consider GPs to be naturally competitive and prone to compare themselves to each other. A more sociable patient-focused GP may have a different working style to a more ^ ((]] vš_-focused GP and the target focused GP may comment negatively on such differences.

Commitment and Investment

The qualitative synthesis highlights the uncertainty around future commitment to investing in future GP practice. The PPI group notes that GPs are a risk adverse people who are driven by financial security. The suggest that younger GP coming out of medical school with financial debts may be less inclined to take on the financial risk of becoming a partner especially with the negative media portrayal and general uncertainty. The PPI group note that salaried GPs are better off than partners as they do not have the financial risks associated with being a partner, and the PPI group pose the ‹µ •š]Would all GPs prefer to be salaried }µo šZ]• Á Ç (}ŒÁ Œ M[

The qualitative synthesis highlights concerns about the difficulties of recruiting new partners to a GP practice to replace a retired GP partner. Because GP practices are independent businesses, GP partners are needed. However, younger GPs may be reluctant to take on partnerships because of the added responsibilities involved. The PPI group note that practice environment / demographic may impact on GP recruitment, with smaller practices suffering most. The PPI group also expressed the view that many GPs may not have good business skills or be trained in HR, and consequently may not be skilled in interviewing and recruitment. They may be less likely to take a professional ‰‰Œ} Z š} o P o šZ]vP• XPX •]Pv]vP }všŒ šU Á]šZ •}u ‰Œ (hence deny/hide/ignore commitment issues.

Impact of Organisational Changes

Referrals

Complex referral systems, more specialised hospitals and delays in communication contribute to GPs experience of fragmentation and a depersonalised healthcare system. (Campbell et al. 2015) (5).The PPI group confirm that in their experiences there is poor linking of secondary and primary care. They observe that decisions to change medications / dose are made in secondary care by nurses and pharmacists and that there is much more choice available in secondary care. When patients then comes back under the responsibility of the GP, the GP may not be familiar with the drug(s) prescribed. This responsibility coupled with a lack of knowledge may cause stress. It was noted by the PPI group that GPs were naturally proud and so less able to admit it if they do not know something and this may compound the issue.

Doctor-Patient relationship

The qualitative synthesis indicates that lack of time with patients means the ability to practise patient-centred continuity of care is compromised. This impacts the W•[‰Œ}(••]}v o µš}v}uÇ values, resulting in diminished job satisfaction for GPs and diminished satisfaction for patients. The PPI group noted how important and valued by patients doctor-patient rapport and personalised knowledge was, and how this could sometimes result in increased efficiency with respect to Œ (ŒŒ o•X dZ Ç Æ‰o]v Z}Á 'W ÁZ} lv}Á• ‰ š] vš[• Z]•š}ŒÇ more likely to prescribe a drug / therapy already prescribed that might reduce the need for secondary care. Such GPs may also make appropriate and timely referrals to secondary care based }v ‰ š] vš•[Œ ‹µ •š v šZ]Œ lv}Áo P }(šZ ‰ š] vš[• Z]•š}ŒÇ

For peer review only - http://bmjopen.bmj.com/site/about/guidelines.xhtml

W š] vš•[u v •

The qualitative synthesis indicates that patient demand (increased number and increased expectations) coupled with a shortage of GPs and available appointments is adding to a feeling of increased pressure which is making some GPs consider retiring. Patient demands may be higher if GP practices are situated in areas of higher deprivation with populations with multiple health and social problems and working with elderly populations with multiple comorbidities and social care needs (Campbell et al., 2015) (5).

The PPI group note that patient demands may also be higher in multicultural communities as they may require more skilled communication from the GPs. The PPI group also note that patients are often ill-informed about how a practice works and so may be unknowingly wasting time and adding to GP pressure. They suggest this could be avoided if patients were provide with information about the structure and function of the practice and were guided in how to most efficiently engage with the practice.

Practice Demands

The qualitative synthesis indicates that GPs in smaller practices were more likely to feel trapped between continuing to work full-time under extreme pressure in order to support colleagues, or to retire completely. Difficulty in recruiting locums precluded many form working part-time. In an µv•µ‰‰}Œš]À vÀ]Œ}vu všU Z À]vP š} š l } absence, ill health, or]o]šÇ (early retirement can add to feelings of burden and stress. Whereas, in the more supportive practice, such scenarios are better managed by the team (Campbell et al., 2015) (5).

The PPI group commented on the finding (from the review of survey studies) that GPs working in very small and in large practices (more than 10 partners) are more likely to quit, with medium sized practices more likely to retain GPs. They suggest that this could be down to smaller practices being less able to adapt and being more reactive, while larger practices do not have the strong relationships in place to support the GPs as larger practices may be less able to get everyone together at the same time and there may be less opportunity for communication and relationship building. Consequently, GPs in larg‰Œ š] • u Ç (o ^]vÀ]•] o _U v}š ^‰ Œš }(less loyal.

Professional Culture

Acceptability of early retirement

/v šZ ‹µ o]š š]À •ÇvšZ •]•U 'W• • Œ] ‰ Œu š]vP ^ µooÇ]vP PPI group acknowledge this and confirm a culture of government bullying via NHS England to salaried GPs. The PPI group think that this is one of the reasons why autonomy is so important to GPs. They also note a historical precedence for GPs to be independent and autonomous due to GP o]v] • šŒ]š]}v ooÇ]vP }‰ Œ š (Œ}u 'W[• o]À]vP Œ}}uX dZ practice managers may be strong characters with too much influence over the practice GPs. They suggest that better training in HR and interviewing for GPs may aid recruiting and could potentially avoid such circumstances.

For peer review only
For peer review only - http://bmjopen.bmj.com/site/about/guidelines.xhtml

BMJ Open

Understanding why primary care doctors leave direct patient care - A systematic review of qualitative research

Journal:	*BMJ Open*
Manuscript ID	bmjopen-2019-029846.R1
Article Type:	Original research
Date Submitted by the Author:	09-Jan-2020

Complete List of Authors:	Long, Linda; University of Exeter, ESMI<br>Moore, Darren; University of Exeter,<br>Robinson, Sophie; University of Exeter Medical School, Evidence Synthesis & Modelling for Health Improvement, Institute of Health Research<br>Sansom, Anna; University of Exeter Medical School, Primary Care<br>Aylward, Alex; Patient and Public Involvement Group<br>Fletcher, Emily; University of Exeter Medical School, Primary Care Research Group<br>Welsman, Jo; University of Exeter, Children's Health and Exercise Research centre<br>Dean, Sarah; PenCLAHRC University of Exeter Medical School,<br>Campbell, John; University of Exeter, Primary Care;<br>Anderson, Rob; University of Exeter, ESMI (Evidence Synthesis & Modelling for Health Improvement)

**Primary Subject Heading**:	General practice / Family practice
Secondary Subject Heading:	General practice / Family practice, Health services research
Keywords:	general practitioner, systematic review, job satisfaction, leave, flexible working, burnout

SCHOLARONE™
Manuscripts

I, the Submitting Author has the right to grant and does grant on behalf of all authors of the Work (as defined in the below author licence), an exclusive licence and/or a non-exclusive licence for contributions from authors who are: i) UK Crown employees; ii) where BMJ has agreed a CC-BY licence shall apply, and/or iii) in accordance with the terms applicable for US Federal Government officers or employees acting as part of their official duties; on a worldwide, perpetual, irrevocable, royalty-free basis to BMJ Publishing Group Ltd ("BMJ") its licensees and where the relevant Journal is co-owned by BMJ to the co-owners of the Journal, to publish the Work in this journal and any other BMJ products and to exploit all rights, as set out in our licence.

The Submitting Author accepts and understands that any supply made under these terms is made by BMJ to the Submitting Author unless you are acting as an employee on behalf of your employer or a postgraduate student of an affiliated institution which is paying any applicable article publishing charge ("APC") for Open Access articles. Where the Submitting Author wishes to make the Work available on an Open Access basis (and intends to pay the relevant APC), the terms of reuse of such Open Access shall be governed by a Creative Commons licence – details of these licences and which Creative Commons licence will apply to this Work are set out in our licence referred to above.

Other than as permitted in any relevant BMJ Author's Self Archiving Policies, I confirm this Work has not been accepted for publication elsewhere, is not being considered for publication elsewhere and does not duplicate material already published. I confirm all authors consent to publication of this Work and authorise the granting of this licence.

For peer review only

Understanding why primary care doctors leave direct patient care - A systematic review of qualitative research

Linda Long[1], Darren Moore[2], Sophie Robinson[1], Anna Sansom[3], Alex Aylward, Emily Fletcher[3], Jo Welsman[4], Sarah G Dean[5], John Campbell[3], Rob Anderson[1]

[1] Evidence Synthesis & Modelling for Health Improvement, Institute of Health Research, University of Exeter Medical School

[2] Graduate School of Education, University of Exeter

[3] Primary Care Research Group and University of Exeter Collaboration for Academic Primary Care (APEx), Institute of Health Research, University of Exeter Medical School

[4] Centre for Biomedical Modelling and Analysis, Living Systems Institute, University of Exeter

[5] Psychology Applied to Health, Institute of Health Research, University of Exeter Medical School

Correspondence to Dr Linda Long: L.Long@exeter.ac.uk

Total word count (excluding abstract): 2494 words

How this fits in

The British GP workforce is said to be in ‘crisis’ with between a third and two fifths of UK GPs intending to leave practice permanently within the next 5 years.

Given the scale of the problem, it is important to understand GP leaving behaviour in the UK.

This systematic review provides a deeper understanding of the complex interplay of key factors and contexts affecting UK GPs’ decisions to leave practice.

This understanding can inform the development of UK GP retention initiatives at national, regional, local area/CCG or practice levels.

For peer review only

Abstract

Background: UK General Practitioners (GPs) are leaving direct patient care in significant numbers. We undertook a systematic review of qualitative research to identify factors affecting GPs' leaving behaviour in the workforce as part of a wider mixed methods study (ReGROUP).

Objectives: To identify factors that affect GPs' decisions to leave direct patient care.

Methods: Qualitative interview-based studies were identified and quality assessed. A thematic analysis was performed and an explanatory model constructed providing an overview of factors affecting UK GPs. Non-UK studies were considered separately.

Results: Six UK interview-based studies and one Australian interview-based study were identified. Three central dynamics key to understanding UK GP leaving behaviour were identified - factors associated with low job satisfaction, high job satisfaction, and those linked to the doctor-patient relationship. The importance of contextual influence on job satisfaction emerged. GPs with high job satisfaction described feeling supported by good practice relationships, while GPs with poor job satisfaction described feeling overworked and unsupported with negatively-impacted doctor-patient relationships.

Conclusions: Many GPs report that job satisfaction directly relates to the quality of the doctor-patient relationship. Combined with changing relationships with patients and interfaces with secondary care, and the gradual sense of loss of autonomy within the workplace, many GPs report a reduction in job satisfaction. Once job satisfaction has become negatively impacted, the combined pressures of increased patient demand and workload, together with other stress factors, has left many feeling unsupported and vulnerable to burnout and ill health, and, ultimately, to the decision to leave general practice. (250 words)

Keywords: general practitioner, systematic review, job satisfaction, leave, flexible working, burnout

PROSPERO protocol CRD42016033876

Article Summary

Strengths and Limitations

- Systematic review conducted and written up with reference to PRISMA guidelines.
- Stakeholder engagement took place during the project and GPs on the team of co-investigators were involved in the development of the review protocol.
- Patients were involved through contributing to a Patient and Public Involvement (PPI) workshop where the explanatory model was discussed.
- Only a small number of UK studies identified and limited ability to translate study findings across countries.
- Synthesis of qualitative evidence relates more or less only to NHS General Practice in England. However, it seems likely that many of these factors are generic within primary care in the rest of the UK.

For peer review only - http://bmjopen.bmj.com/site/about/guidelines.xhtml

Introduction

As described in detail previously (1), general practice in the UK is facing a workforce 'crisis', in part due to so many GPs leaving direct patient care, or reducing their hours, and many others intending to do so (2). While this is a problem being experienced in a number of high-income countries, a report by the Commonwealth Fund in 2015 showed the problem for UK general practice is particularly serious, with nearly 30% of GPs planning to leave general practice within five years (3). In other surveys conducted between 2014 and 2016 the proportion of GPs in the UK saying they would leave general practice within five years varied from 29% to 42% in different regions of England (1,4,5). The most recent (2016) UK survey, of GPs in the South West of England, showed that 70% intend to either quit, reduce their work hours or take a career break in the following five years (5). At the same time GPs appear to be more stressed and more dissatisfied than ever before (6), and more so than GPs and primary care practitioners in most other countries (7).

We undertook a synthesis of qualitative research evidence to identify factors that affect GPs' retention in the workforce as part of a wider mixed methods study (ReGROUP) focusing on retention of experienced GPs or supporting their return to work following a career break. Through better understanding the factors that lead GPs - especially experienced GPs in the UK NHS - to leave direct patient care, the wider ReGROUP study (8) ultimately aims to inform policies and strategies to support GPs returning to work after a career break or retain the experienced GP workforce. By identifying and analysing rich qualitative data from a variety of GP interview studies, we sought to gain a deeper understanding of why GPs are leaving UK practice and to identify and understand how factors may act individually or collectively to affect such decisions.

Aims

This systematic review of qualitative evidence aimed to answer the following question:

What are the factors in the UK and other high income countries which affect GPs' decisions to leave direct patient care?

For peer review only - http://bmjopen.bmj.com/site/about/guidelines.xhtml

Methods

We conducted a systematic review of the qualitative literature in line with our published protocol.

Searches

See Figure 1. In January 2016 and March 2016 articles published in English from 1990 onwards were searched in the following databases: Medline, Medline in Process, PsycInfo, HMIC (Healthcare Management Information Consortium), Cochrane, ASSIA (Applied Social Sciences Index of Abstracts) and Web of Science (Appendix 1, Supplementary File). We performed grey literature searching including online searching, reference checking of relevant studies and forward and backward citation searching. Further update searches were performed in May 2017.

Figure 1 - Medline search strategy

For peer review only

Inclusion criteria

We included qualitative or mixed methods studies which either aimed to assess factors associated with GP leaving behaviour, or which are likely to have generated research data about such factors. We included studies with General Practitioners and other primary care-based generalist doctors practising in high-income countries (Appendix 2, Supplementary File). We sought studies which evaluated any reasons for leaving direct patient care (e.g. early retirement, career breaks, moving to hospital specialities, commissioning or public health, working part-time, or never returning to work after paternal/maternal leave).

Exclusion criteria

Sources were excluded if they were not in English language or highly abbreviated source types (e.g. conference abstracts).

Study selection process

Titles and abstracts of search results were screened against the eligibility criteria, with an initial sample being independently screened by two authors (SR and RA) to establish consistent application of the criteria. Titles and abstracts that could not be excluded were sought as full text articles, and the inclusion criteria applied to these (Figure 2).

For peer review only - http://bmjopen.bmj.com/site/about/guidelines.xhtml

Figure 2 - PRISMA flow diagram showing process of study selection

* Papers excluded at full-text stage are listed in Appendix 3, Supplementary File

Data extraction and quality appraisal

One reviewer (LL) data extracted all published manuscripts and 50% were independently checked by a second reviewer (DM), with any discrepancies resolved through discussion. Study quality was assessed using an adapted version of the Wallace checklist (9) by one reviewer (LL) and 50% independently checked by a second reviewer (DM).

Analysis and synthesis

Data analysis and synthesis broadly followed the principles of thematic synthesis (10) and were conducted in three stages which overlapped to some degree: the coding of text 'line-by-line'; the organisation of these 'free codes' into related areas to construct data-driven 'descriptive themes', and the development of theory-driven 'analytical' themes through the application of a higher level theoretical framework. Thematic analysis of textual data involved study authors' descriptions of their findings as well as primary quotations from GPs.

Of the included studies, two recent data-rich UK papers (11, 12) were coded by one reviewer (LL) and the descriptive themes used to create an overall analytical framework consisting of five categories. The same two key papers were independently coded by a second reviewer (DM) and the analytical framework agreed and modified through discussion. This framework was used to code the remaining studies by one reviewer (LL), with a sample checked by a second reviewer (DM) for consistency. Data, in the form of quotations from the GPs themselves, key concepts or succinct summaries of findings, were entered into QSR's NVivo software (version 11)(13) for analysis. Themes emerging from the UK studies were white-boarded and associations considered. It was acknowledged that the

For peer review only

identified themes could be relevant to more than one category and this was represented in a visual 'explanatory model' (Figure 3) in order to answer the review question. The model was created by one reviewer (LL), independently checked by a second reviewer (DM) and modifications incorporated into the model after discussion. The model was presented and assessed in terms of credibility during an involvement workshop (4 patient participants) and through discussion with the wider ReGROUP project research team.

Patient and Public Involvement

Patients were involved through contributing to a Patient and Public Involvement (PPI) workshop where the explanatory model was discussed (Appendix 4, Supplementary File).

For peer review only

For peer review only - http://bmjopen.bmj.com/site/about/guidelines.xhtml

Results

Study Characteristics

Five studies (six publications) based on qualitative semi-structured interviews with practising or retired GPs were found (11, 12, 14-17), all conducted in England. A further qualitative semi-structured interview study conducted in Australia was found (18). The main characteristics of these studies are shown in Table 1.

Two of the papers reporting studies from England report findings from largely the same set of interviews (11, 12) with the later paper including a larger sample of interviewees, after intentionally recruiting more female GPs and more GPs aged 50-55 years (12).

For peer review only

For peer review only - http://bmjopen.bmj.com/site/about/guidelines.xhtml

Table 1 Characteristics of qualitative interview studies and included GPs

Study	Year of survey(s)	Country or Region	Types of GPs responding	Aim of study	No. GPs (interview setting)	Age of GPs	% female
Doran et al 2016(17)	NS	England	Early leavers age <50 years	To explore the reasons why GPs leave general practice early	21 (by phone)	median age-band 32-54 years	66.7%
Hutchins 2005(14)	NS	England (London)	GP principals near retirement age	Considers the reasons why many GPs are wishing to take early retirement, and measures to help retain them.	20 (at surgery)	NS	55%
Newton et al 2004(15)	NS	England (Northern)	Over 45	To describe "Plans, reasons for, and feelings about retirement"	21 (at surgery or GP home, except 2 by phone)	All over 45 years	38%
Sansom et al 2016*(12)	2015	England (South West)	Experienced GPs 50-60 years old (20 still working, 3 retired)	To investigate the reasons behind intentions to quit direct patient care among experienced general practitioners (GPs) aged 50-60 years.	23* (by phone)	Age range 51-60 years	39%
Campbell et al 2015*(11)	2014-15	England (South West)	Experienced GPs 50-60 years old intending to retire in next 5 years (n=14); GPs who took early retirement in last 5 years (n=3); 15 partners, 2 locums	To explore reasons behind GPs' intentions to quit direct patient care	17* (by phone)	Age 51-60 years	23.5%
					42 (by phone)	NR	NR
Ipsos MORI 2015(16)		England	42 GPs seriously considering leaving practice as well as 23 GPs who had left or were in the process of returning to practice	To identify how the experience of appraisal and revalidation might be influencing intentions to leave general practice	23 (by phone)		

For peer review only

For peer review only - http://bmjopen.bmj.com/site/about/guidelines.xhtml

Study	Year of survey(s)	Country or Region	Types of GPs responding	Aim of study	No. GPs (interview setting)	Age of GPs	% female
Dwan et al 2014(18)	2008 - 2009	Australia	GPs working six or fewer clinical sessions per week	To explore the nature and extent of GPs' paid and unpaid work, why some choose to work less than full-time, and whether sessional work reflects a lack of commitment to patient and the profession	26 (at a location determined by GP participant)	Average age: 47 years (females); 58 years (males)	66%

NS = not stated. *these studies were based on largely the same sample of GP interviews. The later study (Sansom et al, 2016) (12) purposively selected more female GPs and more GPs aged 50-55, to increase the variation of age and sex across the sample

For peer review only

Appraisal and Synthesis

The analysis and synthesis presented below is based on five UK interview-based studies reported in six papers/reports (11, 12, 14-17). The findings of the Australian study (18) are presented separately (Appendix 5, Supplementary File) and discussed in relation to UK findings.

Quality Assessment

The quality of the included qualitative research studies and papers, as assessed using the 14 questions of the adapted 'Wallace tool'(9), ranged from low-quality (16), with 4/14 'yes' ratings on quality criteria, through to moderate-quality (14, 15), with 6/14 'yes' ratings on quality criteria, and up to good-quality (11, 12, 17, 18), with 9/14 'yes' ratings on quality criteria or better (Appendix 6, Supplementary File).

Most studies failed to make explicit the theoretical or ideological perspective of the author (Q2). No studies provided evidence of author reflexivity (Q13). Three UK studies (14-16) and one non-UK study (18) had further limitations in relation to two to four other quality criteria.

All of the themes in the synthesis were informed by at least two studies, and there was at least one good quality study informing every theme (Appendix 7, Supplementary File). The low to moderate-quality UK studies alone did not determine any of the themes, but did provide support for them.

Categories and themes

The synthesis consisted of a series of linked themes affecting whether GPs leave direct patient care or reduce their time commitment to patient care, each of which belongs to one of five categories summarized in the analytical framework below (Table 2) and full details given in Appendix 7, Supplementary File.

Table 2 - Analytic framework showing identified categories and themes around GP's decisions to leave direct patient care

**Undoable /unmanageable**<br><br>- Workload<br>- Pressures<br>- Fear of making mistakes<br>- Training and resources<br>- Patient demands<br>- Practice demands	**Morale**<br><br>- Identity / perceived value<br>- Professional culture<br>- Lack of support<br>-Government/political<br>- Wider community<br>- Negative 'media-bashing'<br>- Job satisfaction<br>- Wellbeing<br>- Work/life balance	**Impact of Organisational Changes**<br><br>- Referrals<br>- Targets and assessments<br>- Doctor-patient relationship<br>- Changing role<br>- Autonomy and control<br>- Reaccreditation
**Projected Future**<br><br>- Viability (of early retirement)<br>- Ageing<br>- Investment and commitment	**Multiple Options and Strategies**<br><br>- Flexible working<br>- Continue and cope<br>- Alternative roles	

For peer review only - http://bmjopen.bmj.com/site/about/guidelines.xhtml

These categories from the qualitative synthesis were, firstly, GPs experiencing working as a GP as 'undoable and unmanageable'. Many GPs are experiencing working as a GP as undoable and unmanageable due, among other reasons, to high/increasing administrative workloads, high/increasing patient demand (both number of patients, and their complexity and higher expectations), together with a perceived lack of training and resources to cope with these pressures.

The second category, 'low morale', was seen to be associated with reductions in the perceived value of GP work (with loss of identity) and changed professional culture (more target- and standards-driven rewards system; multi-disciplinary team-based working (yet for some also lone working/isolating culture); a more aggressive top-down managerial culture within the NHS, and more widespread norms and expectations for early retirement). Low morale was seen as associated with a lack of support from both government and political parties, and negative portrayals of GPs by news media. Morale was also seen to be closely linked with job satisfaction (or dissatisfaction), neglect of personal wellbeing/health and feelings about work-life balance.

The third category was the 'impact of organisational changes'. The perceived key factors under this theme were changes in referrals - both restricted opportunities to refer to secondary care, and higher numbers of (and more complex) referrals from secondary care - as well as a greater focus on targets and assessments, and fears about re-accreditation (including evidence that some GPs might retire early in order to avoid re-accreditation). Some of the organisational changes were considered to have imposed increased clinical and non-clinical responsibilities and work on GPs. Together, such changes were believed to have undermined some of the basic tenets and traditional expectations of being a GP, such as the doctor-patient relationship and having autonomy and control over one's clinical work.

The fourth category was how GPs projected their future, which related to aging, the financial viability of reducing hours or retiring early, and to what extent GPs were personally committed and financially invested in their practices. This included problems linked to whether younger GPs wanted to take on the responsibility of becoming practice partners, and also possible tensions between older and younger GP partners (in the way practices are run, in major investment / refurbishment decisions, or in relation to planning for partner's retiring and needing new partners to buy out their share of a practice).

Finally, the fifth category was called 'multiple options and strategies' and referred to the various ways in which GPs either: continue and cope or - perhaps if less committed or less resilient, or if they can simply afford to financially - decide to leave or go part-time. This theme also highlighted the major importance of flexible working i.e. working reduced hours (e.g. by becoming a locum) as a method of coping and regaining work-life balance and job satisfaction. For others, the adoption of alternative work roles outside general practice, often part-time, allowed use and learning of other skills – either as relief and variety from working as a GP, or for some as a potential alternative career. The kinds of alternative roles and options GP interviewees mentioned included becoming complementary therapists, CCG leads, advisory committee members, or working for pharmaceutical consultancies or teaching in medical schools. Like part-time working, for some these might be clear routes for quitting general practice; but for others, such variety of roles and opportunities for job satisfaction may keep them in general practice.

For peer review only - http://bmjopen.bmj.com/site/about/guidelines.xhtml

Explanatory model and narrative summary of key factors influencing UK GPs

Themes were used to construct an explanatory model (Figure 3). This model makes it possible to 'go beyond' the findings of the primary studies and generate additional concepts, understandings and hypotheses relating to factors influencing GPs' decisions to quit general practice. 'Real world' applicability was confirmed following feedback on the model from patients and project stakeholders during face-to-face discussions in a stakeholder meeting.

Above the explanatory model (in grey), the changing nature of general practice over time is presented separately, providing a contextual lens from which to view the main model. The career path and expectations of UK GPs has changed considerably over the last forty years. Today's GP is expected to be a member of a wider multi-disciplinary team commissioned to deliver national standards of care and has a role barely recognisable to the one many experienced GPs practising in the 1990's remember, where GP partners tended to stay in one practice for most of their career and there was less regulation and a high expectation of autonomy. In the contemporary career model, GPs said they are expected to give up autonomy in many areas of their job and are expected to accommodate increasing government regulation and bureaucracy, which increases stress related to workload, particularly 'paperwork'/record-keeping.

Factors associated with job satisfaction (shaded orange in Figure 3) are listed, along with factors associated with high job satisfaction on the right (shaded red) and factors associated with low job satisfaction on the left (shaded blue). Job satisfaction appears pivotal to whether a GP will successfully adapt and remain in practice, or will become overwhelmed by external influences and pressures and leave the profession. GPs said job satisfaction directly relates to the quality of the doctor-patient relationship, with more time available for GPs to spend with their patients being associated with better job satisfaction. GPs with high job satisfaction describe feeling supported by good practice relationships, while GPs with low job satisfaction describe low morale and feeling unsupported.

For peer review only - http://bmjopen.bmj.com/site/about/guidelines.xhtml

Figure 3 - Explanatory Model of key factors associated with GP leaving behaviour

For peer review only

For peer review only - http://bmjopen.bmj.com/site/about/guidelines.xhtml

Some GPs experiencing low job satisfaction report a lack of good practice relationships, and describe working in a 'blame culture' where they fear litigation (17). Others describe a 'bullying culture', feel undervalued and mistrusted by patients and government, in addition to being inadequately trained in IT, under-resourced, and poorly portrayed in the media (17). Older GPs or GPs with a more conscientious personality may find it more difficult to adapt, and some GPs describe physical symptoms of fatigue and loss of stamina, e.g. women experiencing sleeplessness due to the menopause (11). GPs with low job satisfaction appeared more likely to experience reduced feelings of wellbeing, and experience ill-health and burnout (11). They were also less likely to experience feelings of loyalty to the NHS and more likely to quit (retire, change profession or relocate), exacerbated by a cultural norm of early retirement in the profession (11). Financial incentives and pension arrangements appeared to be more important to GPs with low job satisfaction and, for some GPs, financial incentives (intended to help retain GPs) may cause them to retire earlier rather than stay in practice longer (15).

GP shortages (through poor recruitment and retention) and patient demand are creating pressure on full-time GPs, leading some to consider retiring. Patient demands may be higher in areas of higher deprivation and with populations with multiple health and social problems (11). The impact of GP shortages are most keenly felt in smaller practices, with some GPs feeling trapped between continuing to work full-time under extreme pressure or to retire completely as they fear working part-time would shift the burden of responsibility onto colleagues (11). The explanatory model shows how this situation is compounded by pressures from increased workload (Figure 3, shaded green), particularly from increased administration, as well as from secondary care (12). Increased complexity in referral pathways e.g. hospitals providing increasingly specialised services (i.e. shifting more care to primary care) and delays in communication, contribute to GPs' experiencing a depersonalised, fragmented healthcare system (17). Feelings of uncertainty over the future of general practice are prevalent, with GPs less likely to invest in buildings and make long-term commitments (11). Younger GPs may be more reluctant to take on partnerships because of the added responsibilities and risks involved. For some, poor relationships between older and younger doctors and/or opposing views about how a practice should be run result in older GPs feeling unsupported, less loyal to the NHS and more likely to leave (12).

In summary, UK GPs with poor job satisfaction report feeling overworked and unsupported. Combined with changing relationships with patients and interfaces with secondary care, and the gradual sense of loss of control over large parts of the job, many GPs report a reduction in job satisfaction. Lack of time with patients is perceived to compromise the ability to practise patient-centred care and undermines GPs' professional autonomy and values, resulting in further diminished job satisfaction. Once job satisfaction has become negatively impacted, the combined pressures of increased patient demand and workload, together with other stress factors such as poor IT resources, negative media portrayal, poor practice relationships and a 'bullying' or 'blame' culture, has left many feeling unsupported and vulnerable to burnout and ill health, and, ultimately, to the decision to leave general practice.

For peer review only - http://bmjopen.bmj.com/site/about/guidelines.xhtml

Discussion

The thematic analysis of four qualitative interview studies with UK GPs, two from 2015 and 2016, and two older ones from 2004 and 2005, yielded five overarching types of factors related to GPs leaving or intending to leave direct patient care or reduce their hours, together with more specific sub-themes underlying or linked to these five factors. These themes were categorised into a framework and relationships between identified factors summarised in a visual explanatory model that was developed from them (figure 3). All of these qualitative studies were judged to be of reasonable to good quality.

Overall, the rather negative picture portrayed by the four qualitative interview studies was that UK GPs with poor job satisfaction are also those who feel overworked and unsupported. Many feel part of an over-bureaucratised system, and describe being at the front-end of a service unable to deliver what it promises. Combined with changing relationships with patients and changing interfaces with secondary care, and the gradual sense of loss of control over large parts of the job, many GPs report a reduction in job satisfaction over time. Lack of time with patients is perceived to compromise the ability to practice patient-centred care and continuity of care and, with it, the GPs professional autonomy and values resulting in diminished job satisfaction. Once job satisfaction has become negatively impacted, the combined pressures of increased patient demand and workload together with other stress factors such as poor IT resources, negative media portrayal, poor practice relationships and a perceived 'bullying' or 'blame' culture has left many feeling unsupported and vulnerable to burnout and ill health. Ultimately, for some this leads to their decision to leave general practice altogether or to substantially reduce their clinical hours.

Our explanatory model (Figure 3) highlights the pivotal role of administrative support in enabling GP flexible working. Both Hutchins et al (14) and Doran et al (17) support this finding, suggesting that additional administrative assistance could enable more time to see patients. Given that our synthesis indicates that having sufficient time to see patients is a significant driver for GP job satisfaction, and that job satisfaction is strongly associated with GP retention, increased administrative support may offer a simple solution to the problem of GP retention in the UK. However, it is unlikely that this step alone will solve the problem. Our explanatory model also highlights the complexity of the problem and suggests solutions for retention will not be simple. This is supported by Ipsos MORI (16) who state there can be no 'silver bullet' approach to the complex multifactorial issues underlying current disaffection among UK GPs.

Strengths and weaknesses

Strengths: this systematic review has been conducted and written up with reference to PRISMA guidelines. Potential for transferability of findings to UK practices is based on stakeholder engagement during the project. Relevant stakeholders were involved in the review; several GPs on the team of co-investigators were involved in the development of the review protocol.

The author team consists mainly of academic health researchers employed by the University of Exeter, with one of the author's (AA) being a patient representative. One of the academic health researchers (JC) has previously worked in the NHS as a GP, while another (SD) has previously worked in the NHS as a physiotherapist. Two of the included studies were conducted by two of the co-authors of this systematic review (JC and AS) and the principal investigator of the wider ReGROUP study of which this systematic review is a part (JC) (ref). However, it is noted that neither AS or JC had any involvement in the detailed data extraction or quality assessment of their studies or any of the other studies and as such any prejudice in interpretation of the data is likely to be limited.

For peer review only - http://bmjopen.bmj.com/site/about/guidelines.xhtml

Limitations: limitations include identification of a small number of UK studies. Although a single non-UK study was identified (not reported here), we were not able to translate study findings across countries. In addition, the synthesis of qualitative evidence presented here relates more or less only to NHS General Practice in England. However, it seems likely that many of these factors are generic within primary care in the rest of the UK. We acknowledge that there are limitations from conducting a secondary analysis without coding original transcripts from these studies. Also, of the good quality studies that informed the themes in the synthesis, none explicitly provided a theoretical or ideological perspective of the author (or funder) and none of the authors were reflexive and these limitations may influence individual study research findings and hence the themes identified in this synthesis.

For peer review only

For peer review only - http://bmjopen.bmj.com/site/about/guidelines.xhtml

Conclusions

While recognising the complexity of the current situation, and acknowledging there is unlikely to be a 'silver bullet' solution, the synthesis shows an association between flexible working and improved job satisfaction, potentially delaying retirement. GP's views suggest that stress associated with seeing more patients, including more complex patients, but with the same traditional constraints on appointment times, needs to be addressed. Solutions involving alleviating non-clinical administrative burden, e.g. through additional staff resources resulting in more patient-centred care, may be motivating to many GPs.

Funding

The project was funded by the National Institute for Health Research HS&DR programme (project 253 14/196/02). The views and opinions expressed are those of the authors and do not necessarily reflect those of HS&DR programme, the NIHR, the NHS, or the Department of Health.

Contributor Statement

All authors (LL, DM, SR, RA, AS, AA, EF, JW, SGD and JLC) made a substantial contribution to the conception and/or design of the work.

LL, SR, AS, AA and JW contributed to the acquisition, analysis and interpretation of data for the work.

All authors (LL, DM, SR, RA, AS, AA, EF, JW, SGD and JLC) inputted to drafting the work and/or revising it critically and gave final approval of the version to be published. All are accountable for all aspects of the work in ensuring that questions related to the accuracy or integrity of any part of the work are appropriately investigated and resolved.

Competing interests

None, except that two of the included studies were conducted by two of the co-authors of this systematic review (JC and AS) and the principal investigator of the wider ReGROUP study of which this systematic review is a part (JC). Neither AS or JC had any involvement in the detailed data extraction or quality assessment of their studies or any of the other studies. Also, AA has received personal fees from Northern Eastern Western Devon CCG, Devon Local Medical Committee, British Medical Association, University of Exeter, CLAHRC South West Peninsula, and NHS England Medical Directorate (South), outside of this work.

Acknowledgments

We are very grateful to Simon Briscoe and Chris Cooper for their generous support at the earlier stages of planning this review and for supporting the project's information specialist, Sophie Robinson. We thank the rest of the ReGROUP project co-investigators and researchers for their support and useful comments in progress meetings. We would also like to thank all the PPI group members who attended the mid-review PPI workshop and provided valuable feedback and comments on our emerging review findings and explanatory model. SD is also supported by the National Institute for Health Research (NIHR) Collaboration for Leadership in Applied Health.

Data availability

All data relevant to the study are included in the article or uploaded as supplementary information. No additional data available.

For peer review only - http://bmjopen.bmj.com/site/about/guidelines.xhtml

References

1. Campbell JL, Fletcher E, Abel G, et al. Policies and strategies to retain and support the return of experienced GPs in direct patient care: the ReGROUP mixed-methods study. Southampton (UK): NIHR Journals Library; 2019 Apr. (Health Services and Delivery Research, No. 7.14.) Available from: https://www.ncbi.nlm.nih.gov/books/NBK539934/ doi: 10.3310/hsdr07140
2. Owen K, Hopkins T, Shortland T, Dale J. GP retention in the UK: a worsening crisis. Findings from a cross-sectional survey. BMJ Open. 2019;9(e026048).
3. Martin S, Davies E, Gershlick B. Under pressure: What the Commonwealth Fund's 2015 international survey of general practitioners means for the UK. London: The Health Foundation; 2016.
4. Dale J, Potter R, Owen K, Parsons N, Realpe A, Leach J. Retaining the general practitioner workforce in England: what matters to GPs? A cross-sectional study. BMC Fam Pract. 2015;16(1):140.
5. Fletcher E, Abel G, Anderson R, Richards S, Salisbury C, Dean S, et al. Quitting patient care and career break intentions among general practitioners in South West England: findings of a census survey of GPs. BMJ Open. 2017;7(e015853).
6. Gibson J, Checkland K, Coleman A, Hann M, McCall R, Spooner S, et al. Eighth National GP Worklife Survey UK. 2015.
7. Commonwealth Fund, Henry J. Kaiser Family Foundation. Primary Care Providers' Views of Recent Trends in Health Care Delivery and Payment. Findings from the Commonwealth Fund/Kaiser Family Foundation 2015 National Survey of Primary Care Providers. Issue Brief (Commonw Fund). 2015;24:1-13.
8. UoEMS. PCRG. The changing general practitioner workforce: the development of policies and strategies aimed at retaining experienced GPs and those taking a career break in direct patient care: ReGROUP project. 2016.
9. Wallace A, Croucher K, Quilgars D, Baldwin S. Meeting the challenge: developing systematic reviewing in social policy. Policy Polit. 2004;32(4):455-70.
10. Thomas J, Harden A. Methods for the thematic synthesis of qualitative research in systematic reviews. BMC Med Res Methodol. 2008; 8:45.
11. Campbell J, Calitri R, Sansom A. Retaining the experienced GP workforce in Direct Patient Care (ReGROUP) - Final Report for the South West AHSN. Exeter; 2015
12. Sansom A, Calitri R, Carter M, Campbell J. Understanding quit decisions in primary care: a qualitative study of older GPs. BMJ Open. 2016;6(2):e010592.
13. NVivo qualitative data analysis Software. 11 ed: QSR International Pty Ltd. ; 2015.
14. Hutchins A. Influences on GPs' early retirement, and how to keep them. British Journal of Health Care Management, 11, 367-371, 2005.
15. Newton J. Job dissatisfaction and early retirement : a qualitative study of general practitioners in the Northern Deanery. 2004.
16. Ipsos MORI. Looking to the future: the recruitment, retention and return of GPs (Summary and next steps report for NHS England). London: Ipsos MORI Social Research Institute; 2015.
17. Doran N, Fox F, Rodham K, Taylor G, Harris M. Lost to the NHS: a mixed methods study of why GPs leave practice early in England. Brit J Gen Pract. 2016;66(643):E128-E35.
18. Dwan KM, Douglas KA, Forrest LE. Are "part-time" general practitioners workforce idlers or committed professionals? BMC Fam Pract. 2014;15:154.

For peer review only - http://bmjopen.bmj.com/site/about/guidelines.xhtml

1. Family Practice/ or General Practice/
2. physicians, family/ or physicians, primary care/
3. General Practitioners/
4. Primary Health Care/
5. “primary care”.tw.
6. “general practi$”.tw
7. “family doctor$”.tw.
8. “family physician$”.tw.
9. “family medic$”.tw.
10. (GP or GPs).tw.
11. or/1-10
12. (career$ adj3 (interrupt$ or chang$ or pattern$ or decision$ or leav$ or break$)).tw.
13. (retire$ adj3 (decision$ or medical$ or option$ or choice$ or pattern$ or determin$)).tw.
14. (job$ adj3 (chang$ or leav$)).tw.
15. (work$ adj3 (retention or retain$)).tw.
16. (long adj3 (sick$ or absen$ or ill$)).tw.
17. (burnout or “burn out”).tw.
18. Job Satisfaction/
19. Personnel Turnover/
20. Career Choice/
21. Retirement/
22. or/12-21
23. 11 and 22
24. limit 23 to yr="1990 -Current"

Figure 1 - Medline search strategy

99x99mm (300 x 300 DPI)

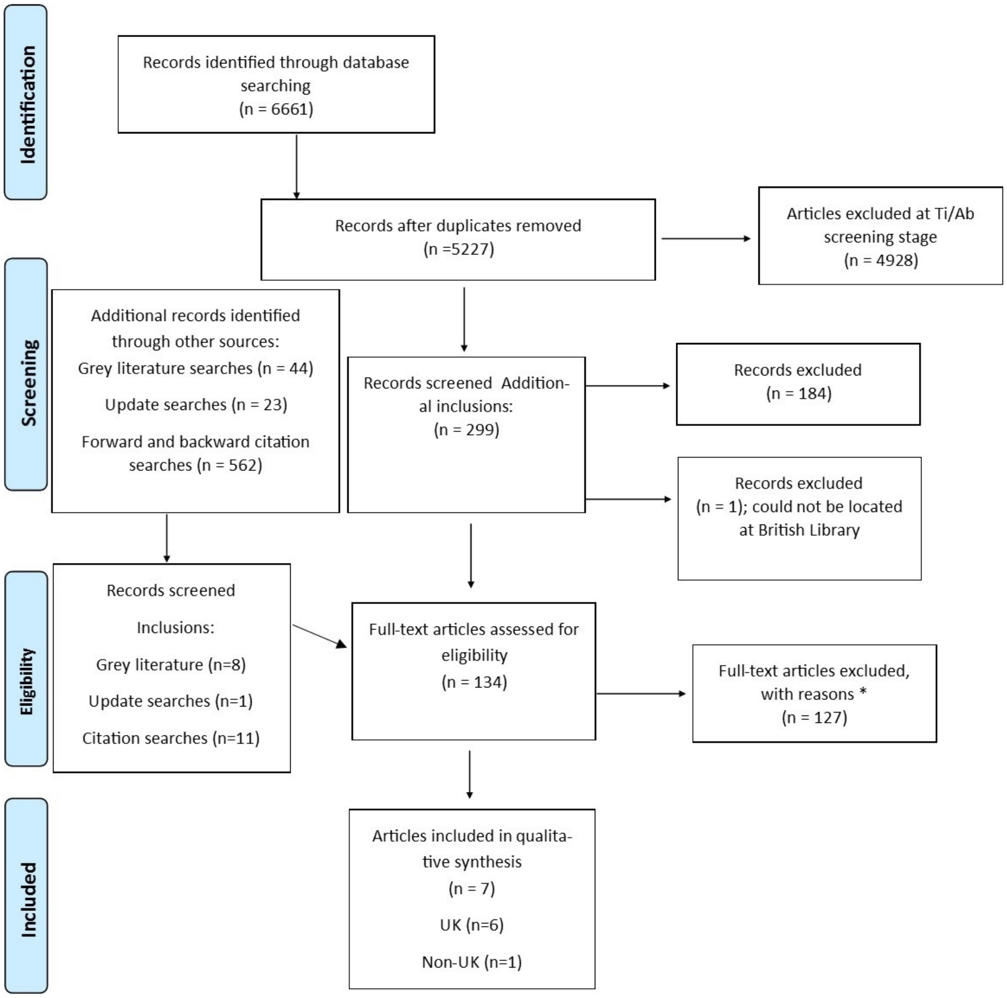

Figure 2 - PRISMA flow diagram showing process of study selection

97x97mm (300 x 300 DPI)

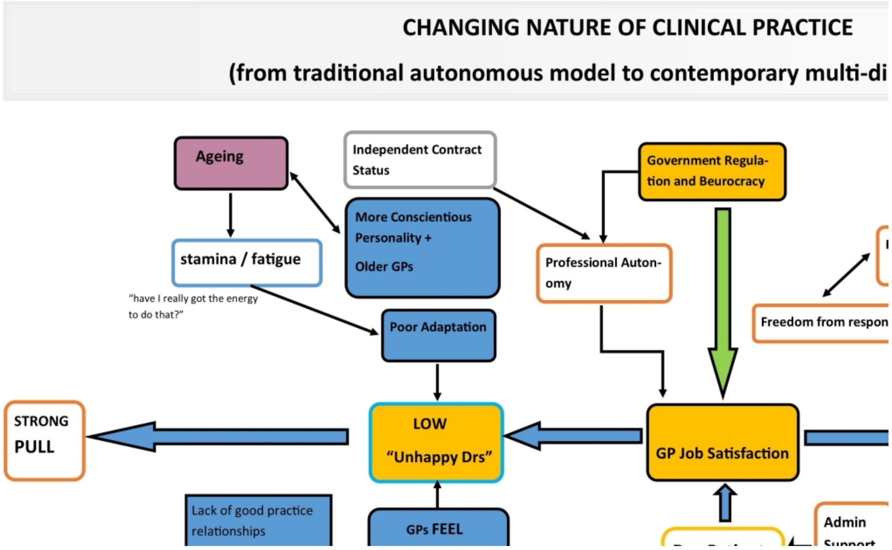

Figure 3 - Explanatory model of GP leaving and quitting behaviour

250x150mm (300 x 300 DPI)

Appendix 1 - Literature search strategies

Database: MEDLINE
Host: Ovid
Data Parameters: 1946 to January Week 3 2016
Date Searched: 29/01/2016
Searcher: SR
Hits: 3655
Strategy:
1. Family Practice/ or General Practice/
2. physicians, family/ or physicians, primary care/
3. General Practitioners/
4. Primary Health Care/
5. "primary care".tw.
6. "general practi$".tw.
7. "family doctor$".tw.
8. "family physician$".tw.
9. "family medic$".tw.
10. (GP or GPs).tw.
11. or/1-10
12. (career$ adj3 (interrupt$ or chang$ or pattern$ or decision$ or leav$ or break$)).tw.
13. (retire$ adj3 (decision$ or medical$ or option$ or choice$ or pattern$ or determin$)).tw.
14. (job$ adj3 (chang$ or leav$)).tw.
15. (work$ adj3 (retention or retain$)).tw.
16. (long adj3 (sick$ or absen$ or ill$)).tw.
17. (burnout or "burn out").tw.
18. Job Satisfaction/
19. Personnel Turnover/
20. Career Choice/
21. Retirement/
22. or/12-21
23. 11 and 22
24. limit 23 to yr="1990 -Current"

Database: MEDLINE(R) In-Process & Other Non-Indexed Citations
Host: Ovid
Data Parameters: 28 January 2016
Date Searched: 28/01/2016
Searcher: SR
Hits: 87
Strategy:
1. "primary care".tw.
2. "general practi$".tw.
3. "family doctor$".tw.
4. "family physician$".tw.
5. "family medic$".tw.
6. (GP or GPs).tw.
7. or/1-6
8. (career$ adj3 (interrupt$ or chang$ or pattern$ or decision$ or leav$ or break$)).tw.
9. (retire$ adj3 (decision$ or medical$ or option$ or choice$ or pattern$ or determin$)).tw.
10. (job$ adj3 (chang$ or leav$)).tw.
11. (work$ adj3 (retention or retain$)).tw.
12. (long adj3 (sick$ or absen$ or ill$)).tw.
13. (burnout or "burn out").tw.

For peer review only
For peer review only - http://bmjopen.bmj.com/site/about/guidelines.xhtml

14. or/8-13
15. 7 and 14

Database: PsycINFO

Host: Ovid
Data Parameters: 1806 to January Week 4 2016
Date Searched: 29/01/2016
Searcher: SR
Hits: 511
Strategy:
1. family medicine/
2. family physicians/
3. general practitioners/
4. primary health care/
5. "primary care".tw.
6. "general practi$".tw.
7. "family doctor$".tw.
8. "family physician$".tw.
9. "family medic$".tw.
10. (GP or GPs).tw.
11. or/1-10
12. (career$ adj3 (interrupt$ or chang$ or pattern$ or decision$ or leav$ or break$)).tw.
13. (retire$ adj3 (decision$ or medical$ or option$ or choice$ or pattern$ or determin$)).tw.
14. (job$ adj3 (chang$ or leav$)).tw.
15. (work$ adj3 (retention or retain$)).tw.
16. (long adj3 (sick$ or absen$ or ill$)).tw.
17. (burnout or "burn out").tw.
18. job satisfaction/
19. employee turnover/
20. occupational choice/
21. retirement/
22. or/12-21
23. 11 and 22
24. limit 23 to yr="1990 -Current"

Database: HMIC (Health Management Information Consortium)

Host: Ovid
Data Parameters: 1979 to November 2015
Date Searched:
Searcher: SR
Hits: 417
Strategy:
1. exp general practice/
2. exp general practitioners/
3. primary care/
4. "primary care".tw.
5. "general practi$".tw.
6. "family doctor$".tw.
7. "family physician$".tw.
8. "family medic$".tw.
9. (GP or GPs).tw.
10. or/1-9

For peer review only - http://bmjopen.bmj.com/site/about/guidelines.xhtml

11. (career$ adj3 (interrupt$ or chang$ or pattern$ or decision$ or leav$ or break$)).tw.
12. (retire$ adj3 (decision$ or medical$ or option$ or choice$ or pattern$ or determin$)).tw.
13. (job$ adj3 (chang$ or leav$)).tw.
14. (work$ adj3 (retention or retain$)).tw.
15. (long adj3 (sick$ or absen$ or ill$)).tw.
16. (burnout or "burn out").tw.
17. job satisfaction/
18. occupational choice/
19. exp retirement/
20. or/11-19
21. 10 and 20
22. limit 21 to yr="1990 -Current"

Database: ASSIA

Host: ProQuest

Data Parameters: n/a

Date Searched: 29/01/2016

Searcher: SR

Hits: 214

Strategy:

1. TI,AB("primary care" OR "general practi*" OR "family doctor*" OR "family physician*" OR "family medic*" OR GP OR GPs) OR SU.EXACT("General practice" OR "General practitioners" OR "Primary health care")
2. TI,AB((career* NEAR/2 (interrupt* OR chang* OR pattern* OR decision* OR leav* OR break*)) OR (retire* NEAR/2 (decision* OR medical* OR option* OR choice* OR pattern* OR determin*)) OR (job* NEAR/2 (chang* OR leav*)) OR (work* NEAR/2 (retention OR retain*)) OR (long NEAR/2 (sick* OR absen* OR ill*) OR (burnout OR "burn out"))) OR SU.EXACT(("Job satisfaction") OR ("Career choice")) OR SU.EXACT.EXPLODE("Early retirement" OR "Mandatory retirement" OR "Retirement")
3. 1 AND 2

Database: Cochrane

Host: Cochrane Collaboration

Data Parameters: CENTRAL: Issue 12 of 12, December 2015; CDSR: Issue 1 of 12, January 2016

Date Searched: 29/01/2016

Searcher: SR

Hits: 75

Strategy:

1 MeSH descriptor: [General Practice] this term only
2 MeSH descriptor: [Family Practice] this term only
3 MeSH descriptor: [Physicians, Family] this term only
4 MeSH descriptor: [Physicians, Primary Care] this term only
5 MeSH descriptor: [General Practitioners] this term only
6 MeSH descriptor: [Primary Health Care] this term only
7 "primary care":ti or "primary care":ab
8 "general practi*":ti or "general practi*":ab
9 "family doctor*":ti or "family doctor*":ab
10 "family physician*":ti or "family physician*":ab

For peer review only

For peer review only - http://bmjopen.bmj.com/site/about/guidelines.xhtml

11 "family medic*":ti or "family medic*":ab
12 (GP or GPs):ti or (GP or GPs):ab
13 (13-#12)
14 (career* near/3 (interrupt* or chang* or pattern* or decision* or leav* or break*)):ti
15 (career* near/3 (interrupt* or chang* or pattern* or decision* or leav* or break*)):ab
16 (retire* near/3 (decision* or medical* or option* or choice* or pattern* or determin*)):ti
17 (retire* near/3 (decision* or medical* or option* or choice* or pattern* or determin*)):ab
18 (job* near/3 (chang* or leav*)):ti
19 (job* near/3 (chang* or leav*)):ab
20 work* near/3 (retention or retain*):ti
21 work* near/3 (retention or retain*):ab
22 long near/3 (sick* or absen* or ill*):ti
23 long near/3 (sick* or absen* or ill*):ab
24 (burnout or "burn out"):ti
25 (burnout or "burn out"):ab
26 MeSH descriptor: [Job Satisfaction] this term only
27 MeSH descriptor: [Personnel Turnover] this term only
28 MeSH descriptor: [Career Choice] this term only
29 MeSH descriptor: [Retirement] this term only
30 (9-#29)
31 #13 and #30

Database: Web of Science
Host: Thomson Reuters
Data Parameters: SCI-EXPANDED and SSCI
Date Searched: 29/01/2016
Searcher: SR
Hits: 1702
Strategy:

1. **TOPIC:** (family (practic* or doctor* or physician* or medic*))
2. **TOPIC:** ("general practi*")
3. **TOPIC:** ("primary care")
4. **TOPIC:** (GP or GPs)
5. 1 OR 2 OR 3 OR 4
6. **TOPIC:** (career near/2 (interrupt* or chang* or pattern* or decision* or leav* or break*))
7. **TOPIC:** (retire* near/2 (decision* or medical* or option* or choice* or pattern* or determin*))
8. **TOPIC:** (job* near/2 (chang* or leav*))
9. **TOPIC:** (work* near/2 (retention or retain*))
10. **TOPIC:** (long near/2 (sick* or absen* or ill*))
11. **TOPIC:** ((burnout or "burn out"))
12. **6 OR 7 OR 8 OR 9 OR 10 OR 11**
13. 5 AND 12
14. Limit to 1990-

For peer review only - http://bmjopen.bmj.com/site/about/guidelines.xhtml

Appendix 2 - List of high-income OECD countries, defined by the World Bank as a country with a gross national income per capita US$12,236 or more in 2016

Australia
Austria
Belgium
Canada
Chile
Czech Republic
Denmark
Estonia
Finland
France
Germany
Greece
Hungary
Iceland
Ireland
Israel
Italy
Japan
Korea, Rep.
Luxembourg
Netherlands
New Zealand
Norway
Poland
Portugal
Slovak Republic
Slovenia
Spain
Sweden
Switzerland
United Kingdom
United States

For peer review only

Appendix 3 - Excluded studies with reasons

	Paper	Reason for exclusion
1	Aseltine RH, Jr., Katz MC. Connecticut physician workforce survey 2008: initial findings on physician perceptions and potential impact on access to medical care. Conn Med. 2008;72(9):539-46.	Not clear whether participants are GPs/PCPs. No examination of factors/associations with/determinants of quitting/intention to quit profession.
2	Aseltine RH, Jr., Katz MC, Geragosian AH. Connecticut physician workforce survey 2009: physician satisfaction, physician supply and	No examination of factors/associations with/determinants of quitting/intention to quit profession.

	patient access to medical care. Conn Med. 2010;74(5):281-91.	
3	Ashworth M., Armstrong D. Sources and implications of dissatisfaction among new GPs in the inner city. Family Practice 1999;16(1):18-22.	No examination of factors/associations with/determinants of quitting/intention to quit profession.<br>Career decisions and progression.
4	Baker, M., J. Williams, and R. Petchey, GPs in principle but not in practice: a study of vocationally trained doctors not currently working as principals. BMJ, 1995. 310(6990): p. 1301-4.	No qualitative data
5	Baker, M., The work commitments of general practitioners: a study of 1986, 1991 and 1996 cohort JCPTGP qualifiers. Monograph series, Nottingham Primary Care Research Unit. 2000, Nottingham: University of Nottingham Division of General Practice. iii,45.	No qualitative data
6	Barnett RC, Gareis KC, Carr PL. Career satisfaction and retention of a sample of women physicians who work reduced hours. Journal of Womens Health. 2005;14(2):146-53.	Not clear whether are GPs/PCPs.
7	Beasley JW, Karsh BT, Sainfort F, Hagenauer ME, Marchand L. Quality of work life of family physicians in Wisconsin's health care organizations: a WReN study. Wisconsin Med Jnl. 2004;103(7):51-5.	No examination of factors/associations with/determinants of quitting/intention to quit profession.<br>Turnover between different employers.
8	Beasley JW, Karsh BT, Hagenauer ME, Marchand L, Sainfort F. Quality of work life of independent vs employed family physicians in Wisconsin: a WreN study. Ann Fam Med. 2005;3(6):500-6.	No examination of factors/associations with/determinants of quitting/intention to quit profession.<br>Turnover between different employers.
9	Brett TD, Arnold-Reed DE, Phan CT, Moorhead RG, Hince DA. Work intentions and opinions of general practice registrars. Medical Journal of Australia, 2009; 191 (2):73-4.	No qualitative data
10	British Medical Association. National survey of GPs: the future of General Practice 2015. BMA. 2015.	No examination of factors/associations with/determinants of quitting/intention to quit profession.
11	Buchbinder SB, Wilson M, Melick CF, Powe NR. Primary care physician job satisfaction and turnover. Am J Manag Care. 2001;7(7):701-13.	<90% are GPs/PCPs and results for GPs not reported separately.<br>No examination of factors/associations with/determinants of quitting/intention to quit profession.<br>Turnover between different employers.
12	Buddeberg-Fischer B, Stamm M, Buddeberg C, Bauer G, Haemmig O, Knecht M, et al. The impact of gender and parenthood on physicians' careers - professional and personal situation seven years after graduation. BMC Health Serv Res. 2010;10:10.	<90% are GPs/PCPs and results for GPs not reported separately.<br>No examination of factors/associations with/determinants of quitting/intention to quit profession.
13	Calitri R, Adams A, Atherton H, Reeve J, Hill NR. Investigating the sustainability of careers in	Not clear whether are GPs/PCPs.

For peer review only - http://bmjopen.bmj.com/site/about/guidelines.xhtml

	academic primary care: a UK survey. BMC Fam Pract. 2014;15:205.	No examination of factors/associations with/determinants of quitting/intention to quit profession.
14	Cameron R, Redman S, Burrow S, Young B. Comparison of career patterns of male and female graduates of one Australian medical school. Teaching and Learning in Medicine. 1995;7(4):218-24.	<90% are GPs/PCPs and results for GPs not reported separately.<br>No examination of factors/associations with/determinants of quitting/intention to quit profession.<br>Career decisions and progression.
15	Carr PL, Gareis KC, Barnett RC. Characteristics and outcomes for women physicians who work reduced hours. Journal of Womens Health & Gender-Based Medicine. 2003;12(4):399-405.	Not clear whether are GPs/PCPs.<br>No examination of factors/associations with/determinants of quitting/intention to quit profession.
16	Chambers M, Colthart I and McKinstry B. Scottish general practitioners' willingness to take part in a post-retirement retention scheme: questionnaire survey. British Medical Journal, 2004. 328(7435): p. 329.	No qualitative data
17	Cheraghi-Sohi S, McDonald R, Harrison S, Sanders C. Experience of contractual change in UK general practice: a qualitative study of salaried GPs. British Journal of General Practice. 2012;62(597):e282-7.	No examination of factors/associations with/determinants of quitting/intention to quit profession.
18	Commonwealth Fund. Primary care providers' views of recent trends in health care delivery and payment: findings from the Commonwealth Fund/Kaiser Family Foundation 2015 national survey of primary care providers. Issue Brief. 2015;24.	<90% are GPs/PCPs and results for GPs not reported separately.
19	Cossman JS. Mississippi's physician labor force: current status and future challenges. J Miss State Med Assoc. 2004;45(1):8-31.	Not clear whether are GPs/PCPs.<br>No examination of factors/associations with/determinants of quitting/intention to quit profession.
20	Crouse BJ. Recruitment and retention of family physicians. Minn Med. 1995;78(10):29-32.	Uses pre-1990 data (from 1982 and 1984).
21	Dale J et al. Retaining the general practitioner workforce in England: what matters to GPs? A cross-sectional study. BMC Family Practice, 2015. 16(1): p. 140.	No qualitative data
22	Davidson JM, Lambert TW, Parkhouse J, Evans J, Goldacre MJ. Retirement intentions of doctors who qualified in the United Kingdom in 1974: Postal questionnaire survey. Journal of Public Health Medicine. 2001;23(4):323-8.	Not clear whether are GPs/PCPs.
23	Degen C, Li J, Angerer P. Physicians' intention to leave direct patient care: An integrative review. Human Resources for Health. 2015;13(1).	Not clear whether are GPs/PCPs.
24	DesRoches CM, Buerhaus P, Dittus RS, Donelan K. Primary care workforce shortages and career recommendations from practicing clinicians. Acad Med. 2015;90(5):671-7.	No examination of factors/associations with/determinants of quitting/intention to quit profession.<br>Career decisions.

For peer review only - http://bmjopen.bmj.com/site/about/guidelines.xhtml

25	Dewa CS, Loong D, Bonato S, Thanh NX, Jacobs P. How does burnout affect physician productivity? A systematic literature review. BMC Health Services Research. 2014;14(1).	Not clear whether are GPs/PCPs.<br>Burnout but not associated with absence from work.
26	Dewa CS, Jacobs P, Xuan Thanh N, Loong D. An estimate of the cost of burnout on early retirement and reduction in clinical hours of practicing physicians in Canada BMC Health Services Research. 2014; 14: 254	No qualitative data
27	Dowell AC, Hamilton S, McLeod DK. Job satisfaction, psychological morbidity and job stress among New Zealand general practitioners. N Z Med J. 2000;113(1113):269-72.	No examination of factors/associations with/determinants of quitting/intention to quit profession.
28	Evans J, Lambert T, and Goldacre M, GP recruitment and retention: a qualitative analysis of doctors' comments about training for and working in general practice. Occasional Paper - Royal College of General Practitioners, 2002(83): p. iii-vi, 1-33.	No qualitative data
29	Farber NJ, Bryson C, Collier VU, Weiner JL, Boyer EG. Work enjoyment, intention to discontinue practice, and burnout in primary care physicians. J Gen Intern Med. 2003;18(Supplement 1):240.	Conference abstract only.
30	French F. General practitioner non-principals benefit from flexible working. 2005.	No qualitative data
31	French F. Why do work patterns differ between men and women GPs? 2006.	No qualitative data
32	Gibson J et al. Eighth National GP Worklife Survey UK. 2015.	No qualitative data
33	Gregory ST, Menser T. Burnout Among Primary Care Physicians: A Test of the Areas of Worklife Model. J Healthc Manag. 2015;60(2):133-48.	No examination of factors/associations with/determinants of quitting/intention to quit profession.<br>Burnout but not associated with absence from work.
34	Hall CB, Brazil K, Wakefield D, Lerer T, Tennen H. Organizational culture, job satisfaction, and clinician turnover in primary care. J. 2010;1(1):29-36.	No examination of factors/associations with/determinants of quitting/intention to quit profession.<br>Turnover between different employers.
35	Hann M, Reeves D, and Sibbald B. Relationships between job satisfaction, intentions to leave family practice and actually leaving among family physicians in England. European Journal of Public Health, 2011. 21(4): p. 499-503.	No qualitative data
36	Heponiemi T, Kouvonen A, Vänskä J, Halila H, Sinervo T, Kivimäki M, et al. Health, psychosocial factors and retirement intentions among Finnish physicians. Occupational Medicine. 2008;58(6):406-12.	Not clear whether are GPs/PCPs.

For peer review only - http://bmjopen.bmj.com/site/about/guidelines.xhtml

37	Heponiemi T, Kouvonen A, Vanska J, Halila H, Sinervo T, Kivimaki M, et al. Effects of active on-call hours on physicians' turnover intentions and well-being. Scandinavian Journal of Work Environment & Health. 2008;34(5):356-63.	<90% are GPs/PCPs and results for GPs not reported separately.<br>No examination of factors/associations with/determinants of quitting/intention to quit profession.<br>Turnover between different employers.
38	Heponiemi T, Kouvonen A, Vänskä J, Halila H, Sinervo T, Kivimäki M, et al. The Association of Distress and Sleeping Problems With Physicians' Intentions To Change Profession: The Moderating Effect of Job Control. Journal of Occupational Health Psychology. 2009;14(4):365-73.	Not clear whether are GPs/PCPs.
39	Heponiemi T, Kouvonen A, Aalto AM, Elovainio M. Psychosocial factors in GP work: the effects of taking a GP position or leaving GP work. Eur J Public Health. 2013;23(3):361-6.	Employment change either *from* or *to* general practice.<br>Turnover between different employers.
40	Heponiemi T, Manderbacka K, Vanska J, Elovainio M. Can organizational justice help the retention of general practitioners? Health Policy. 2013;110(1):22-8.	No examination of factors/associations with/determinants of quitting/intention to quit profession.<br>Turnover between different employers.
41	Heponiemi T, Elovainio M, Presseau J, Eccles MP. General practitioners' psychosocial resources, distress, and sickness absence: a study comparing the UK and Finland. Family Practice. 2014;31(3):319-24.	No examination of factors/associations with/determinants of quitting/intention to quit profession.<br>*All* sickness absence included, not necessarily long-term sickness absence.
42	Hockly A. Could health service reforms make general practitioners ill? Journal of Public Mental Health. 2012;11(2):50-3.	<90% are GPs/PCPs and results for GPs not reported separately.<br>No examination of factors/associations with/determinants of quitting/intention to quit profession.
43	Hojat M, Gonnella JS, Erdmann JB, Veloski JJ, Xu G. Primary care and non-primary care physicians: a longitudinal study of their similarities, differences, and correlates before, during, and after medical school. Acad Med. 1995;70(1 Suppl):S17-28.	No examination of factors/associations with/determinants of quitting/intention to quit profession.<br>Career decisions.
44	Hung DY, Rundall TG, Cohen DJ, Tallia AF, Crabtree BF. Productivity and turnover in PCPs: the role of staff participation in decision-making. Med Care. 2006;44(10):946-51.	<90% are GPs/PCPs and results for GPs not reported separately.<br>No examination of factors/associations with/determinants of quitting/intention to quit profession.<br>Turnover between different employers.
45	Hutchins A. An investigation into the benefits of prolonged study leave undertaken by general practitioners. 2005<br>Hutchins, A., An investigation into the benefits of prolonged study leave undertaken by general practitioners. 2005.	No qualitative data

For peer review only - http://bmjopen.bmj.com/site/about/guidelines.xhtml

46	Jamieson JL, Webber EM, Sivertz KS. Re-entry residency training: opportunities and obstacles. Can Fam Physician. 2010;56(6):e226-32.	Career decisions and progression. Retraining programmes to change speciality and/or retraining as a GP. Balance of focus unclear.
47	Jewett EA, Brotherton SE, Ruch-Ross H. A national survey of 'inactive' physicians in the United States of America: enticements to reentry. Hum Resour Health. 2011;9:7.	<90% are GPs/PCPs and results for GPs not reported separately.
48	Johnson N. General practice careers: changing experience of men and women vocational trainees between 1974 and 1989. British Journal of General Practice. 1993;43(369):141-5.	No examination of factors/associations with/determinants of quitting/intention to quit profession.
49	Jones L, Fisher T. Workforce trends in general practice in the UK: results from a longitudinal study of doctors' careers. British Journal of General Practice. 2006;56(523):134-6.	No examination of factors/associations with/determinants of quitting/intention to quit profession. Career decisions and progression.
50	Joyce CM, Scott A, Jeon SH, Humphreys J, Kalb G, Witt J, et al. The "medicine in Australia: balancing employment and life (MABEL)" longitudinal survey--protocol and baseline data for a prospective cohort study of Australian doctors' workforce participation. BMC Health Serv Res. 2010;10:50.	No examination of factors/associations with/determinants of quitting/intention to quit profession.
51	Joyce CM, Wang WC, McDonald HM. Retirement patterns of Australian doctors aged 65 years and older. Australian Health Review. 2015;39(5):582-7.	<90% are GPs/PCPs and results for GPs not reported separately.
52	Karsh BT, Beasley JW, Brown RL. Employed family physician satisfaction and commitment to their practice, work group, and health care organization. Health Serv Res. 2010;45(2):457-75.	No examination of factors/associations with/determinants of quitting/intention to quit profession.
53	Kelley ML, Kuluski K, Brownlee K, Snow S. Physician satisfaction and practice intentions in Northwestern Ontario. Can J Rural Med. 2008;13(3):129-35.	Not clear whether are GPs/PCPs. Focus on remote rural retention.
54	Kerstein J, Pauly MV, Hillman A. Primary care physician turnover in HMOs. Health Serv Res. 1994;29(1):17-37.	No examination of factors/associations with/determinants of quitting/intention to quit profession. Turnover between different employers.
55	Kilmartin MR, Newell CJ, Line MA. The balancing act: key issues in the lives of women general practitioners in Australia. Med J Aust. 2002;177(2):87-9.	No examination of factors/associations with/determinants of quitting/intention to quit profession.
56	Kirwan M, Armstrong D. Investigation of burnout in a sample of British general practitioners. British Journal of General Practice. 1995;45(394):259-60.	No examination of factors/associations with/determinants of quitting/intention to quit profession. Burnout but not associated with absence from work.

For peer review only - http://bmjopen.bmj.com/site/about/guidelines.xhtml

57	Kuusio H, Heponiemi T, Sinervo T, Elovainio M. Organizational commitment among general practitioners: a cross-sectional study of the role of psychosocial factors. Scand J Prim Health Care. 2010;28(2):108-14.	No examination of factors/associations with/determinants of quitting/intention to quit profession.<br>Turnover between different employers.
58	Kuusio H, Heponiemi T, Vanska J, Aalto AM, Ruskoaho J, Elovainio M. Psychosocial stress factors and intention to leave job: differences between foreign-born and Finnish-born general practitioners. Scand J Public Health. 2013;41(4):405-11.	No examination of factors/associations with/determinants of quitting/intention to quit profession.<br>Turnover between different employers.
59	Langballe EM, Innstrand ST, Aasland OG, Falkum E. The Predictive Value of Individual Factors, Work-Related Factors, and Work-Home Interaction on Burnout in Female and Male Physicians: A Longitudinal Study. Stress and Health. 2011;27(1):73-87.	Not clear whether are GPs/PCPs.<br>No examination of factors/associations with/determinants of quitting/intention to quit profession.<br>Burnout but not associated with absence from work.
60	Lawrence J, Poole P. Career and life experiences of New Zealand women medical graduates. N Z Med J. 2001;114(1145):537-40.	<90% are GPs/PCPs and results for GPs not reported separately.<br>Career decisions and progression.
61	Leese B, Young R, Sibbald B. GP principals leaving practice in the UK. European Jnl Gen Practice. 2002;8(2):62-8.	No examination of factors/associations with/determinants of quitting/intention to quit profession.<br>Examines leaving GP principal job for another GP job, factors for returning.
62	Linzer M, Manwell LB, Williams ES, Bobula JA, Brown RL, Varkey AB, et al. Working conditions in primary care: physician reactions and care quality. Ann Intern Med. 2009;151(1):28-36, W6-9.	<90% are GPs/PCPs and results for GPs not reported separately.<br>No examination of factors/associations with/determinants of quitting/intention to quit profession.
63	Lloyd JR, Leese B. Career intentions and preferences of GP registrars in Yorkshire. Br J GP. April 2006:280-2.	No examination of factors/associations with/determinants of quitting/intention to quit profession.<br>Career decisions and progression.
64	Landon BE, Reschovsky JD, Pham HH, Blumenthal D. Leaving medicine: the consequences of physician dissatisfaction. Med Care. 2006;44(3):234-42.	<90% are GPs/PCPs and results for GPs not reported separately.
65	Lorant V, Geerts C, Duchesnes C, Goedhuys J, Ryssaert L, Remmen R, et al. Attracting and retaining GPs: a stakeholder survey of priorities. British Journal of General Practice. 2011;61(588):e411-8.	<90% are GPs/PCPs and results for GPs not reported separately.<br>No examination of factors/associations with/determinants of quitting/intention to quit profession.<br>Retention and recruitment.
66	Luce A et al. What might encourage later retirement among general practitioners? Journal of Management in Medicine, 2002. 16(4/5): p. 303-310.	No qualitative data
	Martin, S., E. Davies, and B. Gershlick, Under pressure: What the Commonwealth Fund's 2015 international survey of general practitioners	No qualitative data

For peer review only - http://bmjopen.bmj.com/site/about/guidelines.xhtml

	means for the UK. 2016, The Health Foundation: London. p. 37.	
67	Mayorova T, Stevens F, Scherpbier A, van der Velden L, van der Zee J. Gender-related differences in general practice preferences: longitudinal evidence from the Netherlands 1982-2001. Health Policy. 2005;72(1):73-80.	No examination of factors/associations with/determinants of quitting/intention to quit profession.<br>Career decisions.
68	McComb ED. Which psycho-demographic factors predict a doctor's intention to leave New Zealand general practice? New Zealand Medical Journal, 2008. 121 (1273): p.25-36	No qualitative data
69	McKinstry B et al. The feminization of the medical work force, implications for Scottish primary care: A survey of Scottish general practitioners. BMC Health Services Research, 2006. 6.	No qualitative data
70	Misra-Hebert AD, Kay R, Stoller JK. A review of physician turnover: Rates, causes, and consequences. American Journal of Medical Quality. 2004;19(2):56-66.	Not clear whether are GPs/PCPs.<br>No examination of factors/associations with/determinants of quitting/intention to quit profession.<br>Turnover between different employers.
71	Miedema B, Easley J, Fortin P, Hamilton R, Tatemichi S. Crossing boundaries: family physicians' struggles to protect their private lives. Can Fam Physician. 2009;55(3):286-7.e5.	No examination of factors/associations with/determinants of quitting/intention to quit profession.
72	Miedema B, Hamilton R, Fortin P, Easley J, Tatemichi S. The challenges and rewards of rural family practice in New Brunswick, Canada: lessons for retention. Rural Remote Health. 2009;9(2):1141.	No examination of factors/associations with/determinants of quitting/intention to quit profession.<br>Focus on remote rural retention.
73	Moreno-Jiménez B, Gálvez-Herrer M, Rodríguez-Carvajal R, Vergel AIS. A study of physicians' intention to quit: The role of burnout, commitment and difficult doctor-patient interactions. Psicothema. 2012;24(2):263-70.	Not clear whether are GPs/PCPs.
74	Myhre DL, Konkin J, Woloschuk W, Szafran O, Hansen C, Crutcher R. Locum practice by recent family medicine graduates. Can Fam Physician. 2010;56(5):e183-90.	No examination of factors/associations with/determinants of quitting/intention to quit profession.<br>Turnover between different employers.
75	Norman P, Fitter M, Wall T. General practitioners' subjective experience of surgery workload. Social Science and Medicine. 1991 33(2). P.161-6	No qualitative data
76	Nugent A, Black N, Parsons B, Smith S, Murphy AW. A national census of Irish general practice training programme graduates 1990-1996. Irish Medical Journal. 2003. 96 (1) p 10-12	No qualitative data
77	Odom Walker K, Ryan G, Ramey R, Nunez FL, Beltran R, Splawn RG, et al. Recruiting and retaining primary care physicians in urban underserved communities: the importance of	<90% are GPs/PCPs and results for GPs not reported separately.

For peer review only - http://bmjopen.bmj.com/site/about/guidelines.xhtml

	having a mission to serve. Am J Public Health. 2010;100(11):2168-75.	No examination of factors/associations with/determinants of quitting/intention to quit profession.<br>Career decisions and progression.
78	O'Kelly F, O'Kelly M, Ni Shuilleabhain A, O'Dowd T. A National Census of irish General Practice Training Programme Graduates 1997-2003	No qualitative data
79	Pathman DE, Konrad TR, Williams ES, Scheckler WE, Linzer M, Douglas J, et al. Physician job satisfaction, dissatisfaction, and turnover. J. Fam Practice. 2002;51(7):593.	Not clear whether are GPs/PCPs.<br>Turnover between different employers.
80	Pedersen AF, Andersen CM, Olesen F, Vedsted P. Risk of Burnout in Danish GPs and Exploration of Factors Associated with Development of Burnout: A Two-Wave Panel Study. Int Jnl Fam Med. 2013;2013:603713.	No examination of factors/associations with/determinants of quitting/intention to quit profession.<br>Burnout but not associated with absence from work.
81	Plomondon ME, Magid DJ, Steiner JF, MaWhinney S, Gifford BD, Shih SC, et al. Primary care provider turnover and quality in managed care organizations. Am J Manag Care. 2007;13(8):465-72.	Not clear whether are GPs/PCPs.<br>No examination of factors/associations with/determinants of quitting/intention to quit profession.<br>Turnover between different employers.
82	Pit S and Hansen V. Factors influencing early retirement intentions in Australian rural general practitioners. Occupational Medicine, 2014;64, 297-304.	No qualitative data
83	Presseau J, Johnston M, Johnston DW, Elovainio M, Hrisos S, Steen N, et al. Environmental and individual correlates of distress: Testing Karasek's Demand-Control model in 99 primary care clinical environments. British Journal of Health Psychology. 2014;19(2):292-310.	<90% are GPs/PCPs and results for GPs not reported separately.
84	Putnik K, Houkes I. Work related characteristics, work-home and home-work interference and burnout among primary healthcare physicians: a gender perspective in a Serbian context. BMC Public Health. 2011;11:716.	No examination of factors/associations with/determinants of quitting/intention to quit<br>Burnout but not associated with absence from work.
85	Qidwai W, Beasley JW, Gomez-Clavelina FJ. The present status and future role of family doctors : a perspective from the International Federation of Primary Care Research Networks. 2008.	No examination of factors/associations with/determinants of quitting/intention to quit profession.
86	Rabatin J, Williams E, Baier Manwell L, Schwartz MD, Brown RL, Linzer M. Predictors and Outcomes of Burnout in Primary Care Physicians. J Primary Care Community Health. 2016;7(1):41-3.	No examination of factors/associations with/determinants of quitting/intention to quit profession.<br>Burnout but not associated with absence from work.
87	Rittenhouse DR, Mertz E, Keane D, Grumbach K. No exit: An evaluation of measures of physician attrition. Health Services Research. 2004;39(5):1571-88.	Not clear whether are GPs/PCPs.

For peer review only - http://bmjopen.bmj.com/site/about/guidelines.xhtml

88	Ruhe M, Gotler RS, Goodwin MA, Stange KC. Physician and staff turnover in community primary care practice. J Ambulatory Care Manage. 2004;27(3):242-8.	No examination of factors/associations with/determinants of quitting/intention to quit profession.<br>Turnover between different employers.
89	Savageau JA, Ferguson WJ, Bohlke JL, Cragin LJ, O'Connell E. Recruitment and retention of primary care physicians at community health centers: a survey of Massachusetts physicians. J Health Care Poor Underserved. 2011;22(3):817-35.	<90% are GPs/PCPs and results for GPs not reported separately.<br>No examination of factors/associations with/determinants of quitting/intention to quit profession.<br>Turnover between different employers.
90	Schattner PL, Coman GJ. The stress of metropolitan general practice. Med J Aust. 1998;169(3):133-7.	No examination of factors/associations with/determinants of quitting/intention to quit profession.
91	Schofield DJ, Beard JR. Baby boomer doctors and nurses: demographic change and transitions to retirement. Med J Aust. 2005;183(2):80-3.	Not clear whether are GPs/PCPs.<br>No examination of factors/associations with/determinants of quitting/intention to quit profession.
92	Schofield DJ, Fletcher SL, Callander EJ. Ageing medical workforce in Australia--where will the medical educators come from? Hum Resour Health. 2009;7:82.	No examination of factors/associations with/determinants of quitting/intention to quit profession.<br>Workforce planning data.
93	Scott A, Gravelle H, Simoens S, Bojke C, Sibbald B. Job satisfaction and quitting intentions: A structural model of British general practitioners. British Journal of Industrial Relations. Vol 44, Issue 3, p.519-540	No qualitative data
94	Shaw S, Goplen G, Houston DS. Career changes among Saskatchewan physicians. Can Med Assoc Jnl. 1996;154(7):1035-8.	Not clear whether are GPs/PCPs.<br>No examination of factors/associations with/determinants of quitting/intention to quit profession.<br>Career decisions and progression.
95	Shorer Y, Biderman A, Rabin S, Karni A, Levi A, Matalon A. Voluntary departure of family physicians from their workplace: A reflective outlook. Israel Journal of Psychiatry and Related Sciences. 2015;52(2):137-44.	Not clear whether each of four cases described involved leaving general practice. One is about returning to direct patient care. GP emotions around leaving examined not determinants for quitting.
96	Sibbald, B., C. Bojke, and H. Gravelle, National survey of job satisfaction and retirement intentions among general practitioners in England. BMJ, 2003. 326(7379): p. 22.	No qualitative data
97	Shrestha D, Joyce CM. Aspects of work-life balance of Australian general practitioners: determinants and possible consequences. Australian Journal of primary Health. Vol 17, Issue 1, p.40-47	No qualitative data
98	Simoens, S., A. Scott, and B. Sibbald, Job satisfaction, work-related stress and intentions to quit of Scottish GPS. Scottish Medical Journal, 2002. 47(4): p. 80-6.	No qualitative data

For peer review only - http://bmjopen.bmj.com/site/about/guidelines.xhtml

99	Simoens S. Job satisfaction, intentions to quit, and the retention of GPs in England and Scotland. 2002.	No qualitative data
100	Simon AB, Alonzo AA. The demography, career pattern, and motivation of locum tenens physicians in the United States. J Healthc Manag. 2004;49(6):363-75; discussion 75-6.	Not clear whether are GPs/PCPs.<br>No examination of factors/associations with/determinants of quitting/intention to quit profession.<br>Career decisions and progression.
101	Solberg IB, Ro KI, Aasland O, Gude T, Moum T, Vaglum P, et al. The impact of change in a doctor's job position: a five-year cohort study of job satisfaction among Norwegian doctors. BMC Health Serv Res. 2012;12:41.	<90% are GPs/PCPs and results for GPs not reported separately.<br>No examination of factors/associations with/determinants of quitting/intention to quit profession.<br>Career decisions and progression.
102	Solberg IB, Tómasson K, Aasland O, Tyssen R. The impact of economic factors on migration considerations among Icelandic specialist doctors: A cross-sectional study. BMC Health Services Research. 2013;13(1).	<90% are GPs/PCPs and results for GPs not reported separately.<br>No examination of factors/associations with/determinants of quitting/intention to quit profession.
103	Soler JK, Yaman H, Esteva M, Dobbs F, Asenova RS, Katic M, et al. Burnout in European family doctors: the EGPRN study. Family Practice. 2008;25(4):245-65.	No examination of factors/associations with/determinants of quitting/intention to quit profession.<br>Burnout but not associated with absence from work.
104	Statistical Bulletin. Statistics for general medical practitioners in England: 1994-2004. Department of Health Publications. 2005/02.	No examination of factors/associations with/determinants of quitting/intention to quit profession.
105	Stearns J, Everard KM, Gjerde CL, Stearns M, Shore W. Understanding the needs and concerns of senior faculty in academic medicine: building strategies to maintain this critical resource. Acad Med. 2013;88(12):1927-33.	Not clear whether are GPs/PCPs.<br>Academic medicine.
106	Stevenson AD, Phillips CB, Anderson KJ. Resilience among doctors who work in challenging areas: a qualitative study. British Journal of General Practice. 2011;61(588):e404-10.	No examination of factors/associations with/determinants of quitting/intention to quit profession.
107	Sumanen M, Aine T, Halila H, Heikkila T, Hyppola H, Kujala S, Vanska J, Virjo I, Mattila K. Where have all the good GPs gone – where will they go? Study of Finnish GPs. BMC Family Practice, Vol 13, pp 121	No qualitative data
108	Taylor DH, Quayle JA, and Roberts C. Retention of young general practitioners entering the NHS from 1991-1992. British Journal of General Practice, 1999. 49(441): p. 277-280.	No qualitative data
109	Taylor DH, Jr., Leese B. Recruitment, retention, and time commitment change of general practitioners in England and Wales, 1990-4: a retrospective study. BMJ. 1997;314(7097):1806-10.	No examination of factors/associations with/determinants of quitting/intention to quit profession.

For peer review only - http://bmjopen.bmj.com/site/about/guidelines.xhtml

110	Taylor DH, Jr., Leese B. General practitioner turnover and migration in England 1990-94. British Journal of General Practice. 1998;48(428):1070-2.	No examination of factors/associations with/determinants of quitting/intention to quit profession.<br>Turnover between different employers.
111	Taylor DH, Esmail A. Retrospective analysis of census data on general practitioners who qualified in South Asia: who will replace them as they retire? BMJ. 1999;318:306-10.	No examination of factors/associations with/determinants of quitting/intention to quit profession.<br>Workforce planning.
112	Taylor K, Lambert T, and Goldacre M, Future career plans of a cohort of senior doctors working in the National Health Service. Journal of the Royal Society of Medicine, 2008. 101(4): p. 182-190.	No qualitative data
113	Taylor K, Lambert T, Goldacre M. Future career plans of a cohort of senior doctors working in the National Health Service. Journal of the Royal Society of Medicine. 2008;101(4):182-90.	Not clear whether are GPs/PCPs.<br>Career decisions and progression.
114	Taylor KS, Lambert TW, Goldacre MJ. Career progression and destinations, comparing men and women in the NHS: postal questionnaire surveys. BMJ. 2009;338:b1735.	No examination of factors/associations with/determinants of quitting/intention to quit profession.<br>Career decisions and progression.
115	Taylor K, Lambert T, Goldacre M. Career destinations, views and future plans of the UK medical qualifiers of 1988. Journal of the Royal Society of Medicine. 2010;103(1):21-30.	<90% are GPs/PCPs and results for GPs not reported separately.<br>No examination of factors/associations with/determinants of quitting/intention to quit profession.<br>Career decisions and progression.
116	The Royal New Zealand College of General Practitioners. 2015 Workforce Survey	No qualitative data
117	Thommasen HV, Lavanchy M, Connelly I, Berkowitz J, Grzybowski S. Mental health, job satisfaction, and intention to relocate. Opinions of physicians in rural British Columbia. Can Fam Physician. 2001;47:737-44.	Not clear whether are GPs/PCPs.<br>Focus on remote rural retention.<br>Burnout but not associated with absence from work.
118	Thornett A, Cobb S, Chambers R, Mohanna K. Accessing careers support in primary care. Education for Primary Care. 2005;16(1);66-73.	Not clear whether are GPs/PCPs.<br>Career decisions and progression.
119	Toyry S, Kalimo R, Aarimaa M, Juntunen J, Seuril M, Rasanen K. Children and work-related stress among physicians. Stress and Health. 2004;20(4):213-21.	Not clear whether are GPs/PCPs.<br>No examination of factors/associations with/determinants of quitting/intention to quit profession.
120	Van Greuningen M, Heiligers PJ, Van der Velden LF. Motives for early retirement of self-employed GPs in the Netherlands: a comparison of two time periods. BMC Health Services Research. Vol 12, p.467	No qualitative data
121	Virtanen P, Oksanen T, Kivimaki M, Virtanen M, Pentti J, Vahtera J. Work stress and health in primary health care physicians and hospital physicians. Occup Environ Med. 2008;65(5):364-6.	No examination of factors/associations with/determinants of quitting/intention to quit profession.

For peer review only - http://bmjopen.bmj.com/site/about/guidelines.xhtml

		Examines differences between GPs and consultants not factors leading to long term sickness.
122	Wainer J. Work of female rural doctors. Aust J Rural Health. 2004;12(2):49-53.	No examination of factors/associations with/determinants of quitting/intention to quit profession.
123	Woodward CA, Ferrier B, Cohen M, Brown J. Professional activity. How is family physician's work time changing? Canadian Family Physician. Volume 47, p.1414-21	No qualitative data
124	Wordsworth S, Skatun D, Scott A, French F. Preferences for general practice jobs: a survey of principals and sessional GPs. British Journal of General Practice. 2004;54(507):740-6.	No examination of factors/associations with/determinants of quitting/intention to quit profession.<br>Career decisions and progression.
125	Xu G, Veloski JJ, Hojat M, Fields SK. Physicians' intention to stay in or leave primary care specialties and variables associated with such intention. Eval Health Prof. 1995;18(1):92-102.	<90% are GPs/PCPs and results for GPs not reported separately.<br>No examination of factors/associations with/determinants of quitting/intention to quit profession.
126	Young, R., B. Leese, and B. Sibbald, Imbalances in the GP labour market in the UK: Evidence from a postal survey and interviews with GP leavers. Work, Employment and Society, 2001. 15(4): p. 699-719.	No qualitative data
127	Croft, M. (2016). "First 5's in Cornwall - what are their intentions and what influences their career choices?" (Unpublished)	Unpublished (Survey conducted by a GP Academic Trainee research project)

For peer review only - http://bmjopen.bmj.com/site/about/guidelines.xhtml

Appendix 4 – Summary of patient involvement in thematic analysis and explanatory model

The following Patient Involvement discussion points provided colloquial real world perspectives that contextualised our understanding of our literature-based thematic analysis and associated explanatory model.

Flexible Working

While flexible working can bring benefits to individual GPs (young and old) such as freedom from paper work and freedom to pursue other interests, it can increase workload for other practice GPs if they have difficulty recruiting other partner GPs or locums. Discussion with our PPI group suggested that flexible working can have a potentially negative effect on patients who seek appointments with the same GP that they know and have built history and rapport with. If they are consistently inaccessible to them because of their flexible working patterns, patients may experience grief at the loss of the relationship. This could have implications for the NHS as there may be more referrals to secondary care as a consequence. In such circumstances, it is often more acceptable to the patient if the GP retires as this is a predictable and understandable reason for the end of the doctor-patient relationship.

While increasing the availability of locums may relieve pressure on full-time GPs and aid retention of salaried GPs / partners, there was concern from the PPI group that GPs who preferred to travel between GP practices working as locums may choose to do so because it means that they avoid building Doctor - patient relationships. Different personalities may suit different working styles, with permanent salaried GPs / partners having different values and personalities to locums and perhaps valuing the doctor-patient relationship higher.

Continue and Cope

While GPs talk in the semi-structured interviews about strategies that help them to cope with increasing workload and pressures, members of the PPI group note that there is no mention of destructive “coping strategies” such as mis-using alcohol or drugs and no mention of GP use of anti-depressants. There is also no reporting of GPs accessing counselling services in the interviews.

Viability of Early Retirement

The PPI group expressed the view that the GP Cultural norm of acceptability of early retirement may be compounded further by GPs expert knowledge about the human body. Because GPs are more able to predict expected deterioration with age, they may be more likely to plan for early retirement so that they can physically do the things they enjoy.

Ageing

The PPI group noted that holiday entitlement is not mentioned in any of the GP interviews and suggested increased holiday entitlement for aging GPs may help GPs manage their natural fatigue and ultimately improve retention.

Partnership Issues

The qualitative synthesis and explanatory model in this review highlights the importance of good practice relationships for GP retention. When these are not in place, GPs can experience a lack of support which may lead to quitting. The PPI group note that different GPs with different

For peer review only - http://bmjopen.bmj.com/site/about/guidelines.xhtml

personalities / values / working styles may experience conflict when working together in the same practice. PPI members consider GPs to be naturally competitive and prone to compare themselves to each other. A more sociable patient-focused GP may have a different working style to a more "efficient" target-focused GP and the target focused GP may comment negatively on such differences.

Commitment and Investment

The qualitative synthesis highlights the uncertainty around future commitment to investing in future GP practice. The PPI group notes that GPs are a risk adverse people who are driven by financial security. The suggest that younger GP coming out of medical school with financial debts may be less inclined to take on the financial risk of becoming a partner especially with the negative media portrayal and general uncertainty. The PPI group note that salaried GPs are better off than partners as they do not have the financial risks associated with being a partner, and the PPI group pose the question 'Would all GPs prefer to be salaried? Could this be a way forward?'

The qualitative synthesis highlights concerns about the difficulties of recruiting new partners to a GP practice to replace a retired GP partner. Because GP practices are independent businesses, GP partners are needed. However, younger GPs may be reluctant to take on partnerships because of the added responsibilities involved. The PPI group note that practice environment / demographic may impact on GP recruitment, with smaller practices suffering most. The PPI group also expressed the view that many GPs may not have good business skills or be trained in HR, and consequently may not be skilled in interviewing and recruitment. They may be less likely to take a professional approach to legal things e.g. signing contracts, with some preferring to do things "on trust" and hence deny/hide/ignore commitment issues.

Impact of Organisational Changes

Referrals

Complex referral systems, more specialised hospitals and delays in communication contribute to GPs experience of fragmentation and a depersonalised healthcare system. (Campbell et al. 2015) (11). The PPI group confirm that in their experiences there is poor linking of secondary and primary care. They observe that decisions to change medications / dose are made in secondary care by nurses and pharmacists and that there is much more choice available in secondary care. When patients then comes back under the responsibility of the GP, the GP may not be familiar with the drug(s) prescribed. This responsibility coupled with a lack of knowledge may cause stress. It was noted by the PPI group that GPs were naturally proud and so less able to admit it if they do not know something and this may compound the issue.

Doctor-Patient relationship

The qualitative synthesis indicates that lack of time with patients means the ability to practise patient-centred continuity of care is compromised. This impacts the GPs' professional autonomy and values, resulting in diminished job satisfaction for GPs and diminished satisfaction for patients. The PPI group noted how important and valued by patients doctor-patient rapport and personalised knowledge was, and how this could sometimes result in increased efficiency with respect to referrals. They explain how a GP who knows a patient's history and who has a good rapport may be more likely to prescribe a drug / therapy already prescribed that might reduce the need for secondary care. Such GPs may also make appropriate and timely referrals to secondary care based on a patients' request and their knowledge of the patient's history.

For peer review only - http://bmjopen.bmj.com/site/about/guidelines.xhtml

Patients' Demands

The qualitative synthesis indicates that patient demand (increased number and increased expectations) coupled with a shortage of GPs and available appointments is adding to a feeling of increased pressure which is making some GPs consider retiring. Patient demands may be higher if GP practices are situated in areas of higher deprivation with populations with multiple health and social problems and working with elderly populations with multiple comorbidities and social care needs (Campbell et al., 2015) (11).

The PPI group note that patient demands may also be higher in multicultural communities as they may require more skilled communication from the GPs. The PPI group also note that patients are often ill-informed about how a practice works and so may be unknowingly wasting time and adding to GP pressure. They suggest this could be avoided if patients were provide with information about the structure and function of the practice and were guided in how to most efficiently engage with the practice.

Practice Demands

The qualitative synthesis indicates that GPs in smaller practices were more likely to feel trapped between continuing to work full-time under extreme pressure in order to support colleagues, or to retire completely. Difficulty in recruiting locums precluded many from working part-time. In an unsupportive environment, having to take on the responsibility for a partner's absence, ill health, or early retirement can add to feelings of burden and stress. Whereas, in the more supportive practice, such scenarios are better managed by the team (Campbell et al., 2015) (11).

The PPI group commented on the finding (from the review of survey studies) that GPs working in very small and in large practices (more than 10 partners) are more likely to quit, with medium sized practices more likely to retain GPs. They suggest that this could be down to smaller practices being less able to adapt and being more reactive, while larger practices do not have the strong relationships in place to support the GPs as larger practices may be less able to get everyone together at the same time and there may be less opportunity for communication and relationship building. Consequently, GPs in large practices may feel "invisible", not "part of something" and so less loyal.

Professional Culture

Acceptability of early retirement

In the qualitative synthesis, GPs describe a permeating "bullying culture" from the top down and the PPI group acknowledge this and confirm a culture of government bullying via NHS England to salaried GPs. The PPI group think that this is one of the reasons why autonomy is so important to GPs. They also note a historical precedence for GPs to be independent and autonomous due to GP clinics traditionally being operated from a GP's living room. The PPI group describe how sometimes practice managers may be strong characters with too much influence over the practice GPs. They suggest that better training in HR and interviewing for GPs may aid recruiting and could potentially avoid such circumstances.

For peer review only - http://bmjopen.bmj.com/site/about/guidelines.xhtml

Appendix 5 - Australian case study of part-time working

This section separately presents findings of the only included qualitative interview study that was with GPs outside the UK (18). As well being conducted in a different context to the UK studies, the narrower specific focus of this study was on the reasons that Australian GPs preferred to 'work sessionally' – that is part-time (in this study, six or fewer sessions per week).

Flexible working

In this study, many of the GPs "working sessionally" in Australia said that they did so in response to the changing nature of clinical practice, where they were required to work with more complex patients, often with chronic conditions and associated psychological symptoms. Many of the GPs in this study felt that a mix of clinical, non-clinical and unpaid activities attenuated the tiredness one might otherwise feel when working with such patients and allowed more time for being conscientious e.g. reviewing all the patient records before writing a complex referral and providing lots of information. Sessional GPs working in Australia reported that they recognised that "inner resources" were central to providing good quality care, especially when working with complex patients.

Concerns about working flexibly included remuneration, which was considered modest. Also, several GPs found it slightly more difficult to keep up to date clinically.

Continue and cope

The Australian study may offer a different perspective on why some GPs find it easier to cope and continue in the system. One GP suggests that GPs able to adapt to the changing health system may only be able to do so because they are less conscientious. 'If you are doing general practice well clinically, it is quite challenging. I have seen a lot of lazy GPs that palm things off' (Dwan et al., 2014) (18).

Alternative roles

All of the Australian sessional GPs interviewed were in full-time paid employment in health related areas, including education and training, policy, research and academia and medical specialities. All of the interviewed GPs stated that "life's less boring" and "more clinically sustainable and interesting" with flexible work practices.

Doctor-patient relationship

The authors report that the majority of sessional GPs in the study acknowledged increased complexity in treating patients, with a perceived shift away from traditional "disassociated problem solving" involving a mix of semi-acute and chronic care, towards the management of multiple, chronic diseases.

Patient demands

Australian GPs reported that the prevalence of complex, chronic illness and the increasing need for psychological management meant that consultations were time consuming and exhausting. 'Most of my patients … wouldn't be happy if you just printed out a script and handed it to them … What might happen if you do take antibiotics? What might happen if you don't take the antibiotics? [What are] the reasons for taking it? [What are] the reasons not for taking it, you know? I think that takes up a lot of time and I think that's quite exhausting' (Dwan et al., 2014) (18).

For peer review only - http://bmjopen.bmj.com/site/about/guidelines.xhtml

Lack of support

Lack of perceived support towards GPs from the media appears not to be limited to the UK. Australian media portrayal of sessional GPs was reported to be also critical, suggesting that GPs working less than full-time reflected a lack of commitment and that sessional clinical practice is a personal indulgence that disregards the needs of the community.

Job satisfaction

In this study, many of the GPs reported feeling that full-time general practice did not allow them to be the best GP they could be.

'[Like] most GPs I want to do a decent job, and I actually always found that if I go beyond a certain number of sessions I don't think I am doing a decent job anymore' (Dwan et al., 2014) (18)

Wellbeing

Similar dynamics in wellbeing experienced by UK GPs were expressed by sessional GPs in Australia.

The strain of full-time clinical practice was reported to strongly influence many Australian GPs' decisions to work part-time. Sessional clinical practice was seen to offer "downtime", the opportunity to "recharge your batteries". It kept them "fresh," provided time to "catch your breath", and allowed GPs to "maintain good mental and physical health". Therefore, many of the GPs felt that a mix of clinical, non-clinical and unpaid activities attenuated the tiredness one might otherwise feel in full-time clinical practice.

Work-life balance

Cultural influences on work-life balance may be particularly strong. In UK studies, there was no clear gender bias reported for GPs choosing to work less than full-time, with Hutchins et al (14) reporting that GPs of both genders wished to adjust their working hours. However, in this Australian study, the authors suggest that gender strongly influenced female participant's decisions to work less than full-time. Thirteen female GPs and one male GP had dependent children, but only the man did not mention his children or family during the interview. Three of the mothers commented that their spouse's employment required them to work sessionally in order to manage the household and caring responsibilities. A further two women with adult children had significant caring responsibilities

For peer review only

For peer review only - http://bmjopen.bmj.com/site/about/guidelines.xhtml

Appendix 6 - Results of quality assessment

	Newton 2004	Hutchins 2005	Campbell 2015	Sansom 2016	Doran 2016	Dwan 2014	Ipsos MORI, 2015
1) Is the research question clear?	Y	Y	Y	Y	Y	Y	Y
2) Is the theoretical or ideological perspective of the author (or funder) explicit?	N	N	N	N	N	Y	N
2b) Has this influenced the study design, methods or research findings?	CT	CT	CT	CT	CT	N	CT
3) Is the study design appropriate to answer the question?	Y	Y	Y	Y	Y	Y	Y
4) Is the context or setting adequately described?	N	N	Y	Y	Y	Y	N
5) Is the sample adequate to explore the range of subjects and settings, and has it been drawn from an appropriate population?	CT	Y	Y	Y	Y	Y	Y
6) Was the data collection adequately described?	Y	N	Y	Y	Y	N	N
7) Was data collection rigorously conducted to ensure confidence in the findings?	CT	CT	Y	Y	Y	Y	CT
8) Was there evidence that the data analysis was rigorously conducted to ensure confidence in the findings?	Y	Y	Y	Y	Y	Y	N
9) Are the findings substantiated by the data?	Y	Y	Y	Y	Y	Y	CT
10) Has consideration been given to any limitations of the methods or data that may have affected the results?	N	Y	Y	Y	Y	Y	N
11) Do any claims to generalisability follow logically and theoretically from the data?	Y	N	Y	Y	Y	Y	CT
12) Have ethical issues been addressed and confidentiality respected?	CT	Y	Y	Y	Y	Y	Y
13) Is/are the author/s reflexive?	N	N	N	N	N	N	N

Key: Y = Yes, N = No, CT = can't tell. Questions are from tool originally published by Wallace et al (9)

For peer review only

Appendix 7 - Textual thematic analysis

Undoable / Unmanageable

Workload (administration)

All six UK semi-structured interview studies contributed to the theme "workload"

GPs in one study describe often working 12 or more hours per day, and that this was having a significant impact on their ability to do their role and live their lives (16). GPs describe increased administration, both non-clinical and associated with secondary care, preparing for Care Quality Commission (CQC) visits, management targets, regulations and guidelines (11). This caused stress and reduced job satisfaction and was a factor in GPs decisions to leave practice early. Many GPs who had continued in practice beyond the age of sixty had done so because they had been able to delegate paperwork. Alleviation of administration emerged as a high priority for GPs (14).

Pressures

All six UK semi-structured interview studies contributed to the theme of "pressures".

Fear of making mistakes

Time pressure and conflicting priorities meant that some interviewed GPs felt that the care they were giving was sub-standard, leading to disillusionment and a raised anxiety about the risk of making a mistake.

Patient demands

In one study, GPs said demand for patient care was outstripping supply. Contributing factors cited included unrealistic patient expectations arising from patient access to online information about their symptoms while simultaneously being less willing to treat themselves (16). Others describe an increase in the number of patient contacts without a corresponding increase in the number of GPs; and additional workload from secondary care (12).

The pace and complexity of work was felt to be difficult to maintain. GPs felt patient demands may be higher if GP practices were situated in areas of higher deprivation where populations may have many have multiple health and social problems, or in areas with elderly populations with multiple morbidities and social care needs (11) or in areas with high numbers of asylum seekers (14).

GPs report how lack of time with patients corresponded to decreased job satisfaction. "I think what's not so enjoyable now is that actually you are not able to meet people's demands" (Hutchinson 2005) (14).

Practice demands (GP shortages and others working reduced hours)

GPs in smaller practices were reported to be more likely to feel trapped between continuing to work full-time under extreme pressure in order to support colleagues, or to retire completely. However, difficulty in recruiting locums precluded many from working part-time. In an unsupportive practice environment, it was felt that having to take on the responsibility for a practice partner's absence, ill health, or early retirement contributed to feelings of burden and stress. In contrast, in more supportive practices, it was felt that such scenarios are better managed by the team (11).

Training and resources

For peer review only - http://bmjopen.bmj.com/site/about/guidelines.xhtml

GPs report feeling placed in a stressful situation of trying to meet raised patient expectations with insufficient resources and with increased workload being compounded by inadequate training and information technology resources, and thought this may particularly impact older GPs experiencing reductions in stamina and physical limitations. Deteriorating eyesight was noted by three GPs in one study (11), however, computer systems seemed unable to accommodate accessibility issues such as the need for a larger font or fewer icons on the screen.

Morale

Identity / Perceived value of GP work

Five UK semi-structured interview studies (11, 12, 14, 16, 17) contributed to the theme "identity / perceived value"

GPs describe feeling unvalued by both patients and politicians. One GP described feeling how increased patient demand coupled with GP shortages resulted in the perception of the NHS as a "failing brand" in the eyes of the public (Campbell et al 2015) (11).

Professional Culture

Five UK semi-structured interview studies (11, 12, 14, 16, 17) contributed to the theme "professional culture".

Acceptability of early retirement

GPs report feeling that it is common and acceptable amongst their peers to consider and financially plan to take early retirement and, with this in mind, many GPs have made long-term financial plans to make this happen.

Cultural shift

Authors of one study (15) describe GPs trained for a traditional model of general practice who may struggle to adapt to the current one which sees the GP as one member of a multidisciplinary team commissioned to deliver national standards of care. The introduction of payment-related government targets was reported to have impacted on the "moral values" fundamental to general practice of some GPs: 'The government has bred a conniving species of GP ... To an extent you do care about your patients, and you do do your best for them because it's your job ... but you've no longer got any incentive to do anything more than that' (Hutchins 2005) (14).

Bullying top-down culture

GPs describe a permeating "bullying culture"

'There is a really aggressive, vicious, bullying culture that permeates management in the NHS. That then flows all the way down to whoever your locality middle-managers are' (Doran et al., 2016) (17).

Lone working

GPs said that an unintended consequence of having longer and more intense working days was the limited contact with colleagues and sense of isolation that this could cause. This impacted on practice culture of family practices that had traditionally generated positive and supportive work environments (16). GPs said that where practice level support isn't evident, or the GP doesn't feel supported, it can make for an 'everyone for themselves' culture where the decisions about when to leave are based more on self-survival than what is best for the practice.

For peer review only - http://bmjopen.bmj.com/site/about/guidelines.xhtml

Lack of support

Five UK semi-structured interview studies (11, 12, 15-17) contributed to the theme “lack of support”

Government / political

GPs thought more is expected of GPs with lower financial resources and less support in place. Some GPs described being “at the front end of a service unable to deliver what it promises” (Newton, 2004) (15). GPs describe organisational changes resulting in a clash of values and diminishing professional autonomy, as health care became more centralised, standardised, and depersonalised.

Negative media portrayal

Some GPs felt misrepresented by the media and felt frustrated that the more positive aspects of their hard work and professionalism went largely unreported. Being the subject of an ongoing and negative media campaign left many feeling undermined and demoralised:

‘We were targeted in a completely unsympathetic light […] without any recognition of what as a profession we gave to the public really and it did, over time, become very wearing’ (Doran et al., 2016) (17).

Job Satisfaction

Five UK semi-structured interview articles contributed to the theme of “job satisfaction” (11, 12, 14, 15, 17).

Job satisfaction was stated to be a major factor in determining the retirement plans of GPs.

Doran et al report GPs in their study, particularly those with 10 years or more practice experience, feeling their job was not meeting their expectations and there was a loss of intellectual challenge. Many GPs felt the level of satisfaction they were able to derive from general practice had declined considerably as a result of increased government regulation and bureaucratic pressure.

In some cases, GPs describe how they grew to hate their job, or hated “everything around their job”. One former GP described:

‘Passionately adoring my work and my patients, I mean, really I can’t tell you how much I miss them. Absolutely loved the actual job, but everything around the job I hated’ (Doran et al., 2016) (17).

Wellbeing

All six UK semi-structured interview articles contributed to the theme of “wellbeing”.

Many GPs describe themselves as being near burnout (16). Feelings of being overwhelmed, stressed, and losing confidence were also mentioned. One GP described the vicious circle of doctors getting sick, this placing increased pressure on the remaining doctors, who then themselves get sick (11). Time pressure was cited as a factor for GPs not addressing their own health needs:

‘looking after their own well-being was ‘just one more thing to fit in’, and GPs were reluctant to visit their own doctor due to not wanting to be a ‘nuisance patient’ and an awareness that ‘they’re going through the same suffering as you are’ (Sansom et al., 2016) (12).

GP burnout also has implications for the quality of patient care, as described by a GP appraiser:

For peer review only - http://bmjopen.bmj.com/site/about/guidelines.xhtml

‘As GPs got more and more exhausted and burnt out, there was this “I don’t want to know”, there was this disassociation, there was this lack of will to fight to get what patients needed’ (Doran et al., 2016) (17).

Such impacts on the quality of care and the experience of providing care may in turn reinforce patient dissatisfaction and further lower job satisfaction.

Work-life balance

Five interview studies with UK GPs contributed to the theme of “work-life balance” (11, 12, 14, 15, 17).

Issues relating to quality of working life, rather than increased remuneration, emerged as one of the most important factors influencing retention. GPs of both genders wished to adjust their working hours and planned retirement to spend more time with partners and family in the UK. Many stated that the provision of part-time work within their practices was important to enable retention beyond retirement to reduce the pressure of work for that individual, and to enable them to pursue interests they enjoyed. GPs with high job satisfaction said that although they like their job, they felt it encroached on their lives outside work and that they wanted to enjoy hobbies and other interests whilst they were young enough to do so.

Impact of Organisational Changes

Referral volume and complexity

Five UK semi-structured interview studies (11, 12, 14, 16, 17) contributed to the theme “referrals”.

GPs report changes to referral systems resulting in a shift in work load from hospital to primary care, combined with changes in patient demographics and demand. Patient pathways are perceived to be more complex and time-consuming due to “unrealistic expectations of patients” and “hospital doctors lacking resources”. Complex referral systems, with hospitals that focus increasingly on specialised medical needs and delays in communication contribute to GPs’ experience of fragmentation and a depersonalised healthcare system (11).

Targets and assessments

Five UK semi-structured interview studies (11, 12, 14, 16, 17) contributed to the theme “targets and assessments”.

GPs report feeling that management targets, regulations and guidelines increased workload burden (paperwork and bureaucracy) and contribute to stress and loss of job satisfaction. Introduction of the Quality and Outcomes Framework (QOF) was felt by some to be a “tick box exercise” which impacted adversely on the doctor-patient relationship.

‘You spent more time ticking boxes than you did talking to the patients sometimes […] that put more stress on me and I felt it affected my rapport with the patients’ (Doran et al., 2016) (17).

Such monitoring and targets were reported by some older GPs as reflecting a lack of trust and amounting to “micromanagement” from the government.

Doctor-patient relationship

All six UK semi-structured interview studies contributed to the theme of “doctor-patient relationship.”

For peer review only - http://bmjopen.bmj.com/site/about/guidelines.xhtml

GPs reported feeling that the pressures introduced by “impossible targets” and “unrealistic appointment times” had changed the very hallmark of general practice: the doctor-patient relationship. Lack of time with patients meant the ability to practise patient-centred care and continuity of care was perceived to be compromised. As a result, GPs’ professional autonomy and values were felt to be undermined, resulting in diminished job satisfaction for GPs and diminished satisfaction for patients.

Changing role

All six UK semi-structured interview studies contributed to the theme of “changing role”.

Responsibility

GPs reported feeling that an increase in responsibility alongside organisational changes had occurred: ‘Cases were getting more complicated, more was being transferred from the responsibility of the hospital to the responsibility of GPs […], I was spending more and more time doing administrative things and less and less time being able to devote my mental attention to the patients in front of me’ (Doran et al., 2016) (17).

Non-clinical work

Many felt undervalued and their role had been diminished to a ‘government clerk’ or a ‘data clerk for public health and for management’ (17). The GMS contract (2004) was seen to have exacerbated this diminution in role. GPs who continued to practice beyond retirement age had often done so because they had been able to delegate their paperwork, leaving more time for patient consultation, the aspect of general practice they enjoyed.

Rate of change

“Change” was cited as a reason by many GPs for wanting to leave general practice.

Many GPs describe becoming progressively worn down by change over a time period, which several of them said had started in 1990 (15) and that this contributed to low morale. Moreover, difficulties were experienced with perceiving the value of changes, many of which were felt to have been made with no long term vision and for “little health gain”. One GP suggested that more conscientious GPs and older GPs might be less able to adapt and cope with change, and that tolerance to change diminished the longer a GP has been in practice.

Autonomy and Control

Five UK semi-structured interview studies contributed to the theme “autonomy and control”.

GPs described how increased government regulation and bureaucratic pressure has led many GPs to feel an erosion of autonomy and professional control, impacting job satisfaction.

Reaccreditation

Two UK semi-structured interview studies (one ten years older than the other) (12,14) contributed to the theme “reaccreditation”. GPs expressed mixed views about the appraisal and revalidation system. Some found appraisals valuable and helpful and highlighted areas to strengthen through professional development, while others felt they were an additional burden and ineffective (12). Some GPs felt strongly that they should not be exempt from re-accreditation if they continue to work beyond retirement age to ensure competence. However, other GPs mentioned that they would schedule their retirement earlier to avoid their next revalidation.

For peer review only - http://bmjopen.bmj.com/site/about/guidelines.xhtml

Projected Future

Viability of early retirement

Three UK semi-structured interview studies (11, 12, 15) contributed to the theme "viability of early retirement".

Cultural norms of early retirement coupled with good pension provision appear to encourage part-time working and early retirement for GPs in the UK. The 1995 section of the NHS Pension Scheme and so-called '24-hour retirement' were cited by GPs as a way to achieve early retirement (and/or reducing hours) whilst still receiving an adequate income.

GPs with low job satisfaction reported being more likely to plan to leave as soon as they were financially able. For this dissatisfied group, no manner of practical incentives or inducements would keep them at work:

'the more money you gave me the quicker I would be able to retire' (Newton 2004) (15).

Ageing

Four UK semi-structured interview studies (11, 12, 14, 17) contributed to the theme of "ageing".

Cognitive deterioration and fear of incompetency

Some GPs described how cognitive and physical limitations (e.g. deteriorating eye sight) experienced as they got older gave rise to feelings of anxiety and lack of confidence as they feared "unconscious incompetence". Some were concerned that their poorer memory could mean they would be unable to keep up to date. Some GPs recognised their memory and capacity for learning was declining, and said that they would not want to continue in practice if their capacities were inadequate (14).

Resilience

GPs describe feeling that as you get older and stamina decreases, the length of the day is very exhausting and this can impact on GPs' confidence and ability and, consequently, their perceived capacity to continue working in direct patient care.

'There seems to be something that happens when you reach about 55: you start to get feelings of struggling with the work and 60 feels an awful long way away.'(GP interviewee in Campbell et al., 2015) (11).

Feelings of tiredness may be compounded for some female GPs who may experience sleep disturbance during the menopause (12).

Investment and commitment

Five UK semi-structured interview studies contributed to the theme of "investment and commitment" (11, 12, 14, 15, 17).

Partnership issues

GPs reported that poor relationships between older and younger partners arising from differences in values or perspectives could lead to opposing views about how the practice should be run.

For peer review only - http://bmjopen.bmj.com/site/about/guidelines.xhtml

'… it had reached the point where we had young new members who, for their own reasons needed their protected time but hadn't thought through the impact that can have on the rest of the team. You reach a crossroads that says: 'Hang on, I can't mop this up'' (Newton, 2004) (15).

Such tensions resulted in GPs feeling unsupported, less loyal to their practice and having a decreased likelihood of staying on (12). Practice-level changes, such as peers retiring, could also contribute to decisions to leave:

'We've just had three more retirements so nearly all the people who were around when I started have now gone and been superseded by younger, different GPs … my work satisfaction is less and I think a large part of it is because of the changing style of work: the newer doctors work differently. I don't like the way they do it' (GP interviewee in Sansom et al., 2016) (12).

Long-term responsibility

Concerns were evident, of current difficulties of recruiting new partners to a GP practice to replace a retired GP partner (12). However, GPs reported that younger GPs may be reluctant to take on partnerships because of the added risks and responsibilities involved.

Financial investment

GPs reported that concern about the future of general practice meant they may be less likely to invest in buildings and make long term commitments.

Multiple Options and Strategies

Flexible working / Reducing working hours

Five UK semi-structured interview studies (11, 12, 14-16) contributed to the theme "flexible working / reducing working hours".

GPs report that while flexible working can bring benefits to individual GPs (young and old), it can increase workload for other GPs if there is difficulty recruiting other partners or locum GPs. This pressure is more keenly felt in smaller practices, with GPs more likely to feel trapped between continuing to work full-time under extreme pressure in order to support colleagues, or to retire completely.

Continue and cope

Four semi-structured interview studies (11,12,15,17) contributed to the theme "continue and cope".

GPs report that they don't foresee their working situation improving and they vary in their ability to cope (11). GPs said resilience to change, or ability to adapt, may be linked to personality type; one GP describes being an experienced GP with a "robust" personality and "cultivating particular frames of mind" while another talks about having "an enormous amount of experience" and "the right type of personality"(11, 15). Practical coping strategies employed by GPs include looking at work emails from home or in non-work time to try and stay up to date (11), staying late at work, taking work home or changing their appointment times (17). Support given through good working relationships within a GP practice were cited as important for helping GPs cope.

'People are aware of other people's needs and we work together as a group and I think it is a very supportive practice… I don't think I'd still be in the NHS if I was working in another practice, I probably would have left years ago actually' (Sansom et al., 2016) (12).

For peer review only - http://bmjopen.bmj.com/site/about/guidelines.xhtml

Alternative roles for GPs

All six UK semi-structured interview studies contributed to the theme of “alternative roles”.

New professional roles / extended roles

In one study, two GPs reported completing further training in order to leave general practice; one to become a full-time holistic therapist, while the other intended to work part-time as a complementary therapist (11).

Skills transfer

Alternative job roles mentioned by GPs, that used skills transferable from working as a GP, included appraiser, Clinical Commissioning Group lead, advisory committee member, pharmaceutical consultancy work and working for a medical school.

‘A medical degree is one of the most wide-ranging degrees there is: it’s about science, research, communication, empathy, organisation, management - we’re pretty skilled people… Other people want me to do other stuff now; they’ll pay me good money and treat me very differently to what is currently happening to GPs.’ (Campbell et al., 2015) (11).

Professional development / specialisation

One study proposed that for younger GPs, having a medical specialism was thought to provide greater flexibility towards retirement and doctors who already worked part-time in specialist areas outside general practice intended to work entirely in the speciality when they retired (14). Other ‘retired’ GPs undertook locums, or work outside general practice such as Criminal Injuries Compensation Appeal Panel Tribunals or DSS (Department for Social Services) Tribunals (15). Others had combined working as a GP with other jobs, such as teaching, to have a more portfolio career (16).

Relocation

Changing jobs (to other medical jobs outside general practice) and relocating abroad were reported in one study to account for some GPs leaving UK general practice (17).

For peer review only

For peer review only - http://bmjopen.bmj.com/site/about/guidelines.xhtml
